# Transcriptional response to petiole heat girdling in cassava

**DOI:** 10.1038/srep08414

**Published:** 2015-02-12

**Authors:** Yang Zhang, Zehong Ding, Fangfang Ma, Raj Deepika Chauhan, Doug K. Allen, Thomas P. Brutnell, Wenquan Wang, Ming Peng, Pinghua Li

**Affiliations:** 1 The Institute of Tropical Bioscience and Biotechnology (ITBB), Chinese Academy of Tropical Agricultural Sciences (CATAS), Haikou, Hainan 571101, China; 2 Donald Danforth Plant Science Center, St. Louis, Missouri 63132, USA; 3 United States Department of Agriculture, Agricultural Research Service, Plant Genetic Research Unit, St. Louis, Missouri 63132, USA; 4 College of Agronomic Sciences, Shandong Agricultural University, Tai'an, Shandong 271018, China

## Abstract

To examine the interactions of starch and sugar metabolism on photosynthesis in cassava, a heat-girdling treatment was applied to petioles of cassava leaves at the end of the light cycle to inhibit starch remobilization during the night. The inhibition of starch remobilization caused significant starch accumulation at the beginning of the light cycle, inhibited photosynthesis, and affected intracellular sugar levels. RNA-seq analysis of heat-treated and control plants revealed significantly decreased expression of genes related to photosynthesis, as well as N-metabolism and chlorophyll biosynthesis. However, expression of genes encoding TCA cycle enzymes and mitochondria electron transport components, and flavonoid biosynthetic pathway enzymes were induced. These studies reveal a dynamic transcriptional response to perturbation of sink demand in a single leaf, and provide useful information for understanding the regulations of cassava under sink or source limitation.

Cassava (*Manihotesculenta* Crantz), a tropical plant, is the most important root crop globally and provides food for over 800 million people. Since the storage roots of cassava are enriched in starch^1^ and well-adapted to barren soils under drought conditions that are common in the tropical world, cassava is considered an important source of carbohydrates to minimize global food scarcity^2^. Developing countries rely heavily on cassava for daily nutrition, comprising 51% (Africa) 29% (Asia) and 20% (South America) of the total diet on these continents^3^. Cassava tubers consist of 0.24% ash, 0.13% fat, 0.49% protein, 0.15% crude fiber and 98.4% starch on a dry mass basis^4^ and therefore may also be well-suited to bioenergy and biomaterial production.

One reason for cassava's elevated productivity relative to other crops is due to its unusually high rate of photosynthesis. Cassava has a carbon assimilation rate of approximately 43 μmol CO_2_/m^2^/s, that is optimal at high temperature (30–35°C)^5^,^6^,^7^ and is similar or exceeds rates observed in highly productive C_4_ crops including maize, sugarcane and sorghum^8^. As a C_3_ plant, cassava lacks C_4_-Kranz anatomy, however it has a low CO_2_ compensation point, low photorespiration, and highly active phosphoenolpyruvate carboxylase (PEPC, 15–35% of that in maize, a C_4_ plant)^9^,^10^, characteristics that are consistent with efficient carbon assimilation. Field measurements of single-leaf photosynthesis among a wide range of cultivars collected in CIAT (International Center for Tropical Agriculture) have indicated significant positive correlations between leaf photosynthesis, total biomass and root yield^9^,^10^. Thus high-yield cultivars have been developed by exploiting genetic variations in leaf histology and biochemistry that enhanced photosynthesis efficiency and productivity^9^. Given the importance of leaf photosynthesis to starch production in cassava, a number of studies have focused on physiological measurements of photosynthetic parameters but few have assessed correlations with the transcriptome. As a result the molecular mechanisms that regulate photosynthesis and starch accumulation in the species remain unclear. The sequencing of the cassava genome (http://www.phytozome.net/), as well as the rapid improvement of second-generation sequencing techniques—such as Illumina sequencing—now provides an opportunity for detailed exploration of the complex regulatory networks that control photosynthesis in cassava.

The phloem of higher plants is responsible for the transport of metabolic products and for the recycling of mineral nutrients from the shoot to the root or within the shoot from mature leaves to younger non-photosynthetic tissues^11^. Girdling, a technique using cold or ice treatment as well as the application of heat, vibration, electric or osmotic treatment^12^,^13^,^14^,^15^,^16^,^17^,^18^, can inhibit the transport of assimilates in the phloem, resulting in a decreased sink demand. This strategy is commonly used to study the mechanisms of source-sink balance. In a comprehensive study of multiple species, Goldschmidt and Huber^19^ developed a hot-wax-girdling method to prevent the export of assimilates from leaves of soybean (*Glycine max L. Merr. cv Ransom*), cotton (*Gossypiumhirsutum L.*), cucumber (*Cucumissativus L.*), tomato (*Lycopersiconesculentum Mill.*), broad bean (*Viciafaba L.*), sunflower (*Helianthus annuus L.*), and bean (*Phaseolus vulgaris L.*). They reported different degrees of inhibition of photosynthesis that were species dependent and grouped results on the basis of leaf carbohydrate partitioning attributes (i.e. starch or sucrose stores). Using cold girdling, Krapp and Stitt^20^ evaluated the direct and indirect mechanisms underlying the “sink-regulation” of photosynthesis in spinach (*Spinaciaoleracea L.*), and found that the inhibition of phloem export caused by cold girdling led to rapid changes in metabolism, as well as an almost simultaneous change in gene expression. Jeannette *et al.*^21^ also confirmed that carbohydrate accumulation feedback inhibited photosynthesis on gene expression levels, though the signals evoking the inhibition response remain less clear.

The “end-product inhibition of photosynthesis” hypothesis, as proposed ca. 150 years ago by Boussingault^22^ stated that the accumulation of assimilates in an illuminated leaf may be responsible for the decrease in net photosynthesis rate. In most plant species, sucrose is the principal form of carbohydrate translocated through the veins from carbon-exporting source leaves to carbon-importing sink tissues^23^. To investigate transcriptional changes associated with photosynthesis and the genes affected by sucrose transport, and to identify genes that play a key role in balancing source supply and sink demand, we blocked phloem export by a modified heat treatment (first used in stems) for petioles on cassava leaves. RNA-Seq libraries were generated from phloem-blocked leaves and non-blocked controls to examine the transcriptional network response of photosynthesis in individual leaves. The data were assessed bioinformatically to identify transcriptional changes in genes that may play key roles in balancing source supply and sink demand in cassava. Other physiological and metabolic measurements were made to further link changes in the transcriptome with plant function.

## Results

### Effects of heat girdling on starch metabolism and photosynthesis

**Figure 1:**
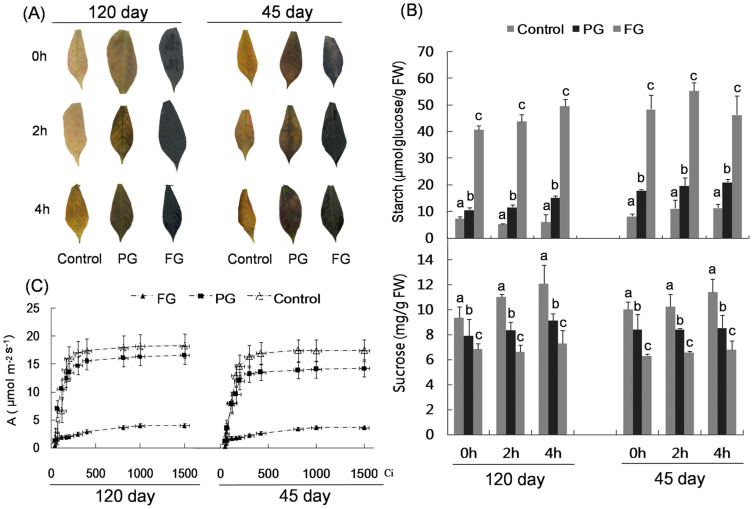
Physiological analysis of the control, partial girdled (PG) and fully girdled (FG) leaves. (A) An IKI stain showing starch accumulation in control, PG and FG cassava leaf lobes. Leaves were collected from 120- and 45-day-old plants at 0, 2, and 4 h after the lights came on, cleared of photosynthetic pigments, and stained with IKI. (B) Starch and sucrose measurements for control, PG and FG leaves. All leaves were harvested and processed concurrently. Letters above the standard error bars indicate whether the treatment had a significant influence within leaves from plants of a given age (P < 0.05) based on analysis of variance (ANOVA) followed by Tukey's honestly significant difference (HSD) test. (C) A/Ci curve. Pn (A) vs intercellular CO_2_ concentration (Ci; μmolmol^−1^). Measurements were performed at an average ambient relative humidity of 46.6 ± 3.4% and an irradiance of 1200 μmol m^−2^s^−1^.

Heat-girdling was performed at the end of the day on the petiole of 120-day-old cassava plants that were developing storage roots (Supplemental Fig. 1, roots in pot). To minimize the effects of wounding and heat stress, a second girdling regime was utilized in which only a partial area of the petiole was heat girdled (partial girdling, PG). The fully girdled leaves (full girdling, FG) had significant amount of accumulated starch at the beginning of the light cycle; however, very little starch remained in the control. Since partially girdled leaves incompletely blocked the phloem transportation, starch accumulation was considerably lower relative to fully girdled plants after a night cycle, but remained 1.5- to 2-fold higher than the non-treated control (Fig. 1a and 1b). The sucrose content was modestly depressed in treated leaves and glucose and fructose contents did not differ significantly from the control (Supplemental Table S1). The development of storage roots may alter the source-sink balance of cassava; thus, we performed an identical treatment on the leaves of cassava seedlings (45 days old), and similar results were obtained (Fig. 1). Together the data indicate heat girdling is an effective way to alter carbon translocation from the leaf.

An A/Ci curve was generated after heat girdling to examine the influence of treatments on the photosynthetic assimilation rate. As shown in Fig. 1c, heat girdling inhibited starch remobilization at night and significantly repressed CO_2_ assimilation during the light cycle. This repression was not influenced by the development of the storage root. At the same CO_2_ concentration, the assimilation rate was considerably lower in heat-girdled leaves than in the control, and this repression was more severe in FG than in PG. Net photosynthesis rates (Pn), stomata conductance (Gs), and intercellular CO_2_ concentration (Ci) were all inhibited by the treatment (Supplemental Fig. 2).

### Mapping the RNA-Seq reads to the cassava reference genome

To identify the genes and pathways that responded to starch accumulation caused by heat girdling, we constructed RNA-Seq libraries from control and heat-girdled leaves from plants in the seedling and storage-root-development stages at 0, 2, and 4 h after light exposure. After examining the effectiveness of heat girdling on starch levels by IKI staining of individual leaf lobes, four leaves from each effective treatment were pooled for library construction and two biological replicates were performed (see Methods). A total of 36 libraries were sequenced using Hi-seq2500. Since the libraries from PG 4 h at the seedling stage were of low quality, we removed this time point from further analysis. A total of 272 million raw reads was generated from the leftover 30 samples. After trimming adapters and filtering out low-quality reads, ~238 million reads (~87.5%) were uniquely aligned to the cassava genome, and ~1.7% of reads aligned to multiple locations. For the unique reads, 74.7% were mapped to exon/protein-coding regions, 14.6% were mapped to the splicing junctions, and others were mapped to intron (3.1%) and intergenic (7.6%) regions. We defined 6,462 novel splicing junctions, which may be used to identify novel transcripts from the cassava genome.

We identified a total of 22,284 genes expressed in the cassava leaves. Among these, 6,704 were considered significantly differentially expressed between treatment and control based on a cuffdiff pairwise analysis with the FDR controlled at 0.001. To confirm the accuracy and reproducibility of the Illumina RNA-Seq results, specific genes showing increased, decreased or unchanged expression after treatments were subjected to quantitative real-time (qRT)-PCR confirmation (Supplemental Table S2). As shown in Supplemental Fig. 3, the qRT-PCR expression trends of these genes showed significant similarities to the RNA-Seq data (r^2^ = 0.85), which independently validated the gene expression analyses by RNA-seq.

### Functional category enrichment analysis reveals pathway changes after heat girdling

**Figure 2:**
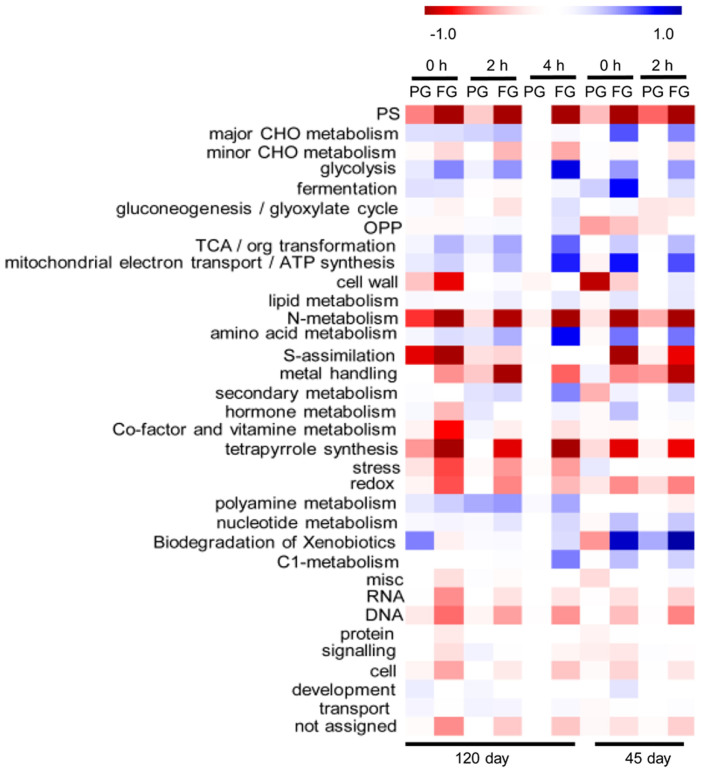
A heat map showing under and over represented functional classifications among genes which were differentially regulated in partial girdled (PG) and fully girdled (FG) cassava leaves. This analysis was performed using the Pageman software package http://mapman.gabipd.org/web^43^. Red boxes indicate that genes in a category were generally down-regulated in the corresponding set of samples relative to controls while blue boxes indicate that genes in a category were generally up-regulated in the corresponding set of samples. Fisher's exact test was applied to test for enrichment of functional category and FDR was controlled for by Benjamini and Hochberg's procedure at P = 0.01.

The log_2_ fold changes of 6,704 significantly differentially expressed genes at each time point were entered in the Mapman and Pageman software for pathway analysis. Functional category enrichment was calculated using Fisher's exact test with FDR controlled by Benjamini and Hochberg's procedure at P = 0.01. As presented in Fig. 2, blocking starch remobilization by heat girdling significantly influenced photosynthesis. The photosynthesis pathways were significantly down regulated compared to the control. This effect was more dramatic in fully girdled plants. Similar trends were observed for the N metabolism and tetrapyrrole synthetic pathways. In contrast, the major CHO (carbohydrate) catabolic pathways including: glycolysis, TCA and mitochondrial electron transport pathways were significantly up-regulated in the heat-girdled leaves; and most dramatic in fully girdled leaves. The GO term enrichment assay gave similar results (Supplemental Table S3). To identify individual genes that responded to pathway changes, we used heat maps to quantify the expression of DE genes that were enriched in each functional category.

### Pathways repressed due to girdling

**Figure 3:**
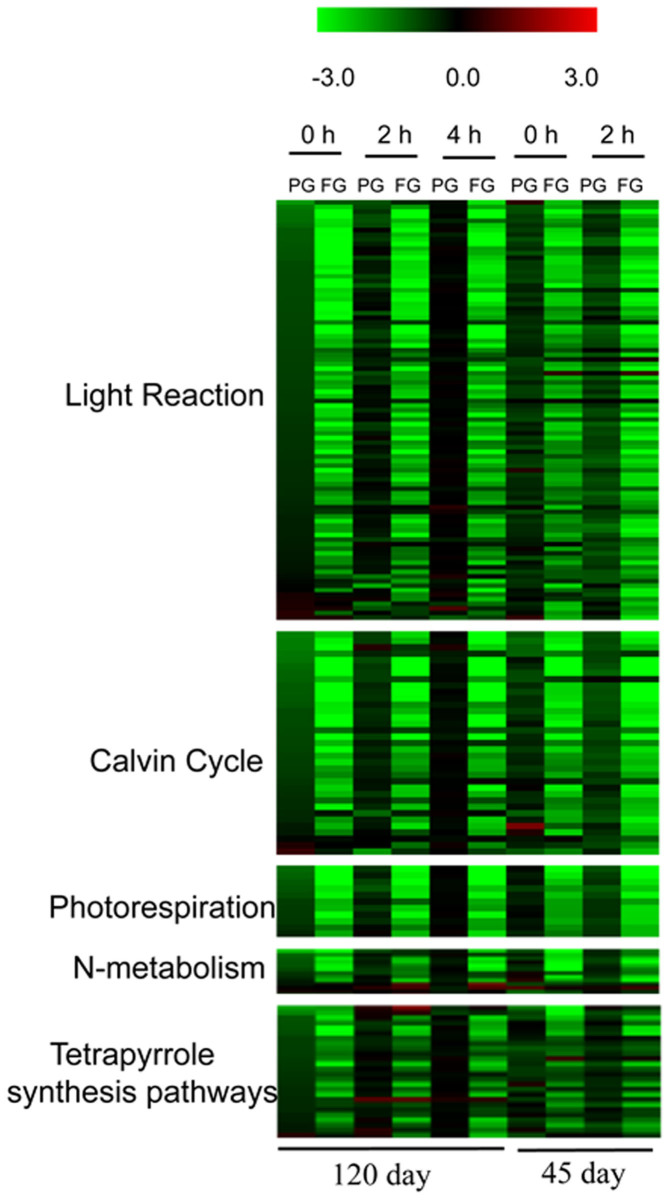
Effects of girdling on the expression of genes associated with photosynthesis, N metabolism and tetrapyrrole synthesis pathways. The values in red and green indicate log_2_ fold increases and decreases, respectively, in expression of individual genes within each category in treated leaves relative to control leaves.

As shown in Fig. 3 (Supplemental Table S4), all photosynthesis-related pathways—including the light reactions, carbon assimilation and photorespiration were repressed significantly by the heat-girdling treatment. Since PG blocked starch remobilization only partially, the decrease in gene expression in PG was not as severe as in FG (Fig. 3, Supplemental Table S4). In addition, the gene expression patterns were similar at two different developmental stages of cassava plants. Girdling resulted in gene expression differences in the light harvesting components including PSI polypeptide subunits, such as PSA-D, PSA-F, PSA-L and PSA-N. PSII-related genes were also affected including chlorophyll a-b binding protein, PSII polypeptide subunits (e.g., PSBO2; PSBQ), NADH DH (NADH: plastoquinone dehydrogenase) complex, NDH-M and NDH-N, and ATP synthase complexes. Calvin-cycle-related gene expression was reduced after heat-girdling treatments including the expression of aldolase, FBPase (fructose-1,6-bisphosphatase), GAPA (glyceraldehyde-3-phosphate dehydrogenase A), phosphoglycerate kinase, PRK (phosphoribulokinase) and RPE (ribulose-phosphate 3-epimerase). In addition, the expression of RCA (Rubiscoactivase), which activates Rubisco, was decreased 1.4–1.8-fold by PG treatment and 7.4–12.2-fold by FG treatment. Consistent with RCA, expression of the RuBisCO small subunit, which is involved in net photosynthetic CO_2_ assimilation and photorespiratory carbon oxidation^24^, was decreased by 1.4–1.8- and 6.1–8.5-fold by the PG and FG treatments, respectively (Supplemental Table S4).

The expression of genes encoding photorespiratory steps also decreased. Glycolate oxidase, alanine-glyoxylate transaminase, serine hydroxymethyltransferase, hydroxypyruvatereductase, and glycerate kinase were all expressed at lower levels in PG and FG samples (Supplemental Table S4). Likewise, Fd-GOGAT and glutamine synthetase 2 (GS2), the chloroplastic enzymes responsible for the reassimilation of photorespiratory ammonia, were also significantly reduced. Other genes that pertain to nitrogen metabolism were affected including NIR1 (nitrite reductase 1) and NIA1 (nitrate reductase 1), key enzymes in nitrate metabolism, and a putative glutamate receptor perceived to be involved in nitrogen transduction (Fig. 3, Supplemental Table S4).

Inspection of tetrapyrrole biosynthesis genes (Fig. 3, Supplemental Table S4) that are tied to light harvesting through chlorophyll biosynthesis also produced a number changes in expression. Chlorophyll biosynthesis was repressed by PG and FG treatments, including magnesium chelatase GUN5; magnesium-protoporphyrin IX monomethyl ester (oxidative) cyclase required for the biosynthesis of chlorophyll; GSA2 (glutamate-1-semialdehyde 2,1-aminomutase), an upstream control point in chlorophyll biosynthesis; HO (hemeoxygenase), necessary for the biosynthesis of the phytochromechromophore; and GUN4, which functions as a regulator of chlorophyll synthesis and intracellular signaling.

### Pathways induced by girdling treatment

**Figure 4:**
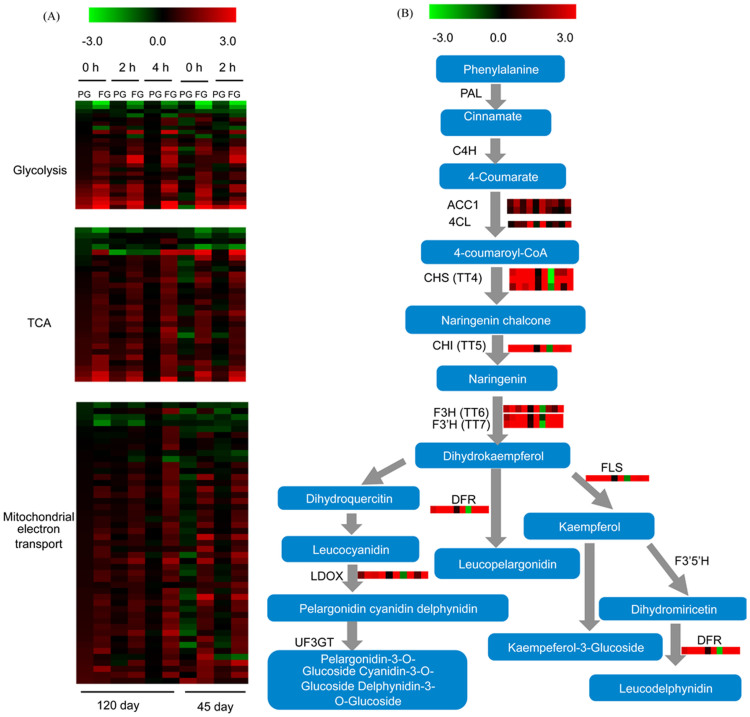
Effects of girdling on the expression of genes associated with (A) Glycolysis, TCA cycle, and mitochondrial electron transport pathways and (B) flavonoid biosynthetic pathway. The values in red and green indicate log_2_ fold increases and decreases, respectively, in the expression of specific genes in partial girdled (PG) and fully girdled (FG) leaves compared to control leaves. Experimental conditions 1–6 (left to right) are leaves from 120-day-old plants that were developing storage roots: (1) 0 h PG leaves, (2) 0 h FG leaves, (3) 2 h PG leaves, (4) 2 h FG leaves, (5) 4 h PG leaves, and (6) 4 h FG leaves; conditions 7–10 (left to right) are leaves from 45-day-old seedlings that have no storage roots developed: (7) 0 h PG leaves, (8) 0 h FG leaves, (9) 2 h PG leaves, and (10) 2 h FG leaves.

Genes involved in catabolic pathways including: glycolysis, the TCA cycle and mitochondrial electron transport changed due to girdling (Fig. 4A, Supplemental Table S5). The affected genes encoding enzymes related to glycolysis included glyceraldehyde-3-phosphate dehydrogenase, which catalyzes 3-glycerol phosphate aldehyde into 1,3 -2 glyceric acid phosphate and pyruvate kinase that produces pyruvate from phosphoenolpyruvate as well.

Within the tricarboyxlic acid cycle (TCA) pyruvate dehydrogenase E1 and E2, citrate synthase, succinate dehydrogenase, succinyl-CoA ligase, and isocitrate dehydrogenase were all expressed at higher levels after heat girdling (Supplemental Table S5). The increase was more significant in FG compared with PG. Similar to TCA, most mitochondrial electron transport/ATP synthesis-related genes were induced after heat girdling (Supplemental Table S5), including cytochrome c, cytochrome c oxidase, cytochrome c reductase, and the genes encoding the NADH dehydrogenase complex and F-ATPase subunits. Likewise, metabolite transporters for the mitochondrial membrane, including ATP: ADP antiporters, and mitochondrial dicarboxylate carriers were also upregulated, possibly reflecting changes in the demand for ATP to maintain homeostasis.

Girdling leaves exhibited a response to stress leading to enhanced gene expression in the flavonoid biosynthetic pathway that produces anthocyanins were induced. As shown in Fig. 4b (Supplemental Table S5), the genes encoding ACC1 and 4CL3, which act in the general phenylpropanoid pathways; CHS (TT4); CHI (TT5), F3H (TT6), and F3'H (TT7), the early steps upstream of the branch points of flavonoid biosynthesis pathways; DFR (TT3), which is important for anthocyanidin biosynthesis; and FLS, which is required for biosynthesis of flavonols, were significantly induced. As flavonoids play central roles in protection against harmful UV-light and excess visible light, the induction of this pathway may be indicative of imbalance of light energy in the starch accumulated leaves after treatment. This may also explain observed changes in cytochrome b5 family member (Supplemental Table S5), which expression were increased by >100-fold after FG compared to the non-treatment control. Cb5 is an electron transfer protein localized mainly in the ER membrane, and may be involved in multiple biochemical activities in cells as an electron donor^25^.

### Change in starch and sucrose metabolism

**Figure 5:**
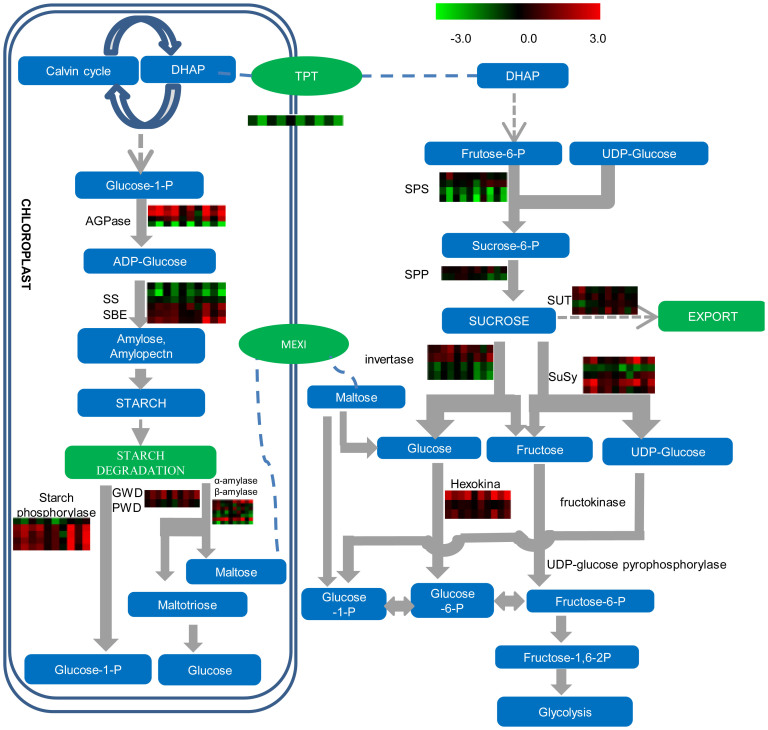
Effects of girdling on the expression of genes associated with starch and sucrose metabolism pathways. The values in red and green indicate log_2_ fold increases and decreases, respectively, in expression in partial girdled (PG) and fully girdled (FG) leaves compared to control leaves. Each row represents the expression pattern of a specific gene. Each column represents a different experimental condition. Experimental conditions 1–6 (left to right) are leaves from 120-day-old plants that were developing storage roots: (1) 0 h PG leaves, (2) 0 h FG leaves, (3) 2 h PG leaves, (4) 2 h FG leaves, (5) 4 h PG leaves, and (6) 4 h FG leaves; conditions 7–10 (left to right) are leaves from 45-day-old seedlings that have no storage roots developed: (7) 0 h PG leaves, (8) 0 h FG leaves, (9) 2 h PG leaves, and (10) 2 h FG leaves.

Heat-girdling resulted in presence of starch during the light cycle the following day (Fig. 1) and influenced the expression of starch and sucrose metabolism-related genes (Fig. 5, Supplemental Table S6). Some starch biosynthetic genes, such as starch synthase, were expressed at lower levels after treatment; however, other genes, including APL3, which encodes the large subunit of ADP-glucose pyrophosphorylase and catalyzes the first and rate-limiting step in starch biosynthesis; and APS1, which is the major small subunit isoform required for large subunit stability and starch-branching enzymes, such as SBE2 were induced. Genes encoding enzymes involved in starch breakdown including starch phosphorylase (PHS2), and several sucrose-biosynthesis-related genes, including SPS (sucrose phosphate synthase) and SPP (sucrose-phosphatase), were significantly altered. These changes may indicate a reduction in sucrose production because of the imposed limitation on export and translocation and possibly some futile production and degrading of starch as a mechanism to initially cope metabolically with the unanticipated presence of starch at dawn. Generally, cell wall invertases (vacuolar invertases) were expressed at lower levels after heat-girdling treatment though one neutral invertase was less affected and SuSy, a key enzyme in sucrose breakdown in leaves, was induced by the treatments. Sugar sensors and transporters including: HXK1 and its homolog HKL1, SUT and STP13 were induced by girdling. STP13 is a hexose-specific/H^+^ symporter that improves plant growth and nitrogen use and is involved in programmed cell death (PCD)^26^.

### Responses of transcription factors to the heat girdling treatment

**Figure 6:**
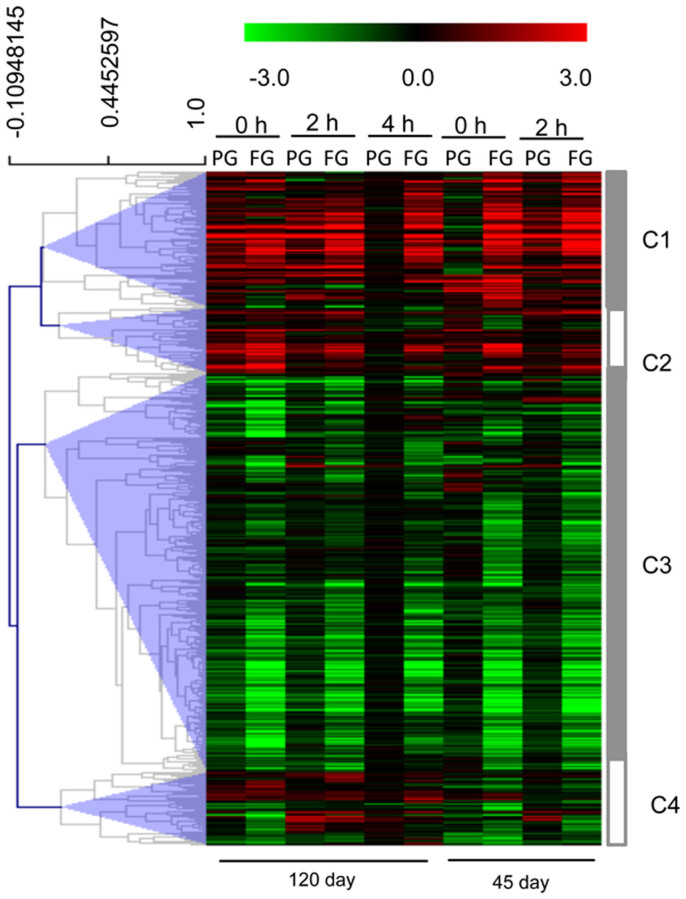
Effects of girdling on the expression of transcription factors. For each row, the values in red and green indicate log_2_ fold increases and decreases, respectively, in expression of a specific gene in partial girdled (PG) and fully girdled (FG) leaves compared to the control.

After manual curation, we identified 431 putative TF genes that showed differential expression due to the girdling treatment. Hierarchical clustering segregated genes into four major groups according to their expression patterns (Fig. 6, Supplemental Table S7). The C1 and C2 clusters included genes expressed at higher levels by the treatment, and C3 included genes decreased in expression by the treatment. The 47 genes in C4 showed no clear expression trends. There were 87 genes in the C1 and 42 in the C2 clusters. Among them, several transcription factor family members that likely play a role in anthocyanin biosynthesis were identified, including those of the bHLH family TT8, MYB family (MYB111 and MYB4), and MADS family (AGL19 and AGL20) homologs. Apart from anthocyanins, a number of genes that respond to stress were noted. C3H zinc finger; C2H2 zinc finger (STZ), AP2/EREBP; and GRAP; and genes that respond to nutritional status, such as bHLH (cassava4.1_015506m.g), which responds to iron ion starvation; and MYB62, which responds to phosphate starvation, were included in the C1 and C2 clusters. A total of 255 genes were included in C3. Many TFs that are important to light signaling were included in this cluster, e.g. PAP2.4, CIB1, PIL1, PIL5 and ZFN1; as well as TFs that regulate photosynthesis activity, such as CIA2 (chloroplast import apparatus 2), which is important for protein chloroplast targeting; and GPRI1, which regulates photosystem II assembly and chlorophyll biosynthetic processes. Corresponding to the change in TFs associated with light signaling, we found down regulation of the expression of the blue-light photoreceptor CRY1 and PAS/LOV PROTEIN B, and genes involved in red/far-red signaling, such as FRS10 (FAR1-related sequence 10), and SPA family protein (SUPPRESSOR OF *PHYA-105*). However, the expression of phytochrome B (cassava4.1_000531m.g, Supplemental Table S7) was induced significantly by the treatments.

## Discussion

Examining the coordination between source and sink demand in cassava is important to improve our understanding of regulatory aspects that govern photosynthesis and carbohydrate partitioning and facilitate breeding and genetic engineering efforts aimed at increasing yield. By overexpression of a modified bacterial AGPase in the storage roots of cassava, Ihemere^27^ reported a significantly increased AGPase activities and biomass in both storage roots and above-ground tissues, which demonstrated that increasing source strength maybe an effective way to increase yield in sink tissues. However, the impact of reduced sink on source metabolism remains unclear. Heat-girdling of the petiole at the end of the light cycle during developing (120 days) and non-developing (45 days) storage root stages of cassava was used to perturb a single leaf sink demand. Others have used heat^18^,^20^,^28^ or cold girdling^12^,^13^,^14^ to explore source to sink relationships though the timing of the girdling process and length of studies varies extensively depending upon specific scientific questions. We were most interested in examining the photosynthetic response that occurs when girdling prevented the turnover of starch which would mimic a change in sink demand in a single leaf. The girdling process presumably inhibited starch remobilization at night based on the extensive starch accumulation and lower sucrose contents in the heat-girdled leaves at the beginning of the light cycle (Fig. 1). The extensive starch accumulation during the light cycle in leaves may also interference photosynthesis, as photosynthetic assimilation was significantly decreased (Fig. 1c).

Understanding the direct cause of changes in photosynthetic metabolism has remained challenging. Though the correlation between starch accumulation and photosynthetic inhibition in our study are consistent with other girdling studies at various times^18^,^19^,^29^,^30^; the direct linkage between the two remains questionable. Starch stores generally show an acute response to girdling that includes starch production and photosynthetic decline; however past studies have indicated starchless lines can also show a similar effect^18^. Using cold-girdling treatment of the low-starch-accumulating plant spinach, Krapp and Stitt^19^ concluded that girdling inhibited photosynthesis only minimally; in contrast, in plants that accumulate greater amounts of starch such as maize, a significant decrease in photosynthesis occurred after heat girdling^20^. Thus the qualitative observation that the girdling process varies with species and starch accumulation also highlights the complexity of carbohydrate partitioning and photosynthesis. During the seedling stage, the majority of C_3_ cassava plants accumulate starch in the leaves during the day, which is broken down at night to maintain growth^31^. Following their development, storage roots represent a strong sink that can drive assimilation; however, the phenotype resulting from inhibition of starch remobilization at night in a single leaf was similar in heat-girdled leaves irrespective of the presence of storage roots implying that the plant developmental state did not significantly impact the results. Though heat-girdling appeared to inhibit the remobilization of starch at night; it did not increase sucrose content indicating that sucrose production and starch breakdown were coordinated^31^.

We observed a significant decrease in the expression of genes associated with the light reactions, the carbon assimilation, and photorespiration (Fig. 2, Supplemental Table S4) suggesting that the increased levels of starch may be inhibiting photosynthetic metabolism and resulting less starch and sucrose production. A 10-fold reduction in rubisco activase and rubisco small subunit expression in FG, and a 2–3-fold reduction in PG, implied a possible decrease in carbon fixation capacity, which may influence sugar metabolism in the cells, as reported previously in sugarcane^33^. Additionally, the expression of the major sugar sensors, HXK1 and HKL1 were induced and the cytochrome b5 family member was increased by >100-fold after FG treatment (Supplemental Table S5, S6), which has been linked to sugar starvation by interacting with sucrose and sorbitol transporters to regulate their affinity for substrate sugars^32^.

Carbon and nitrogen metabolism are tied through amino acid metabolism biosynthesis and the production and consumption of energy and reduced nucleotide cofactors^34^. Transcripts including Fd-GOGAT, GS2, and NIR1, were reduced by the girdling treatment (Fig. 3) and may reflect the coordination with carbon and redox. Nitrate assimilation to ammonium and subsequent production of organic nitrogen in the leaf is an intensive process that requires a significant number of electrons supplied through photosynthetic metabolism. The altered capacity to carry out photosynthetic functions would result in an imbalance in carbon, energy, and redox and lead to diminished organic nitrogen biosynthesis^35^.

A series of changes that are likely linked to stress metabolism were also observed including anthocyanin and flavonoid biosynthetic pathway genes. Similar to heat-girdled maize and cold-girdled sugarcane^20^,^33^, anthocyanin gene expression was elevated in girdled leaves. Arabidopsis pho3 mutants that accumulate soluble sugars and starch due to the lack of a phloem-loading Suc-proton symporter also exhibit increased anthocyanin accumulation^36^. Transcription factors including TT8, MYB111 and MYB4 (Figure 6, Supplemental Table S7) that regulate anthocyanin production were induced as were genes, e.g. FLS, involved in other flavonoid biosynthetic pathways. Anthocyanins and other non-pigmented flavonoids serve to absorb UV light that protects plant DNA from UV damage^37^ and their induced expression may suggest a stress response when photosynthesis was repressed. The accumulation of starch in leaves would presumably lead to an imbalance between light harvesting and carbon assimilating steps such that light stress occurred possibly resulting in a transcriptional response paralleling the exposure to UV. Nonetheless other antioxidants, such as catalase, SOD and peroxiredoxin, were principally not induced after girdling treatment (Supplemental table S8), suggesting that the leaves with extensive starch accumulation did not exhibit significant free radical generation after 2 to 4 hrs light exposure. Furthermore, photorespiratory genes that could partially mitigate the generation of free radicals through redistribution of reducing equivalents were repressed.

Our data suggest that heat girdling in a single leaf inhibits starch remobilization at night, which causes starch accumulation and inhibits photosynthesis during the light cycle. These changes strongly influence plant primary metabolism, including starch, sucrose metabolism, and nitrogen metabolism and secondary metabolism through the flavonoid and anthocyanin biosynthetic pathways. Changes at the transcriptional level were consistent with maintaining coordination between source and sink, and provide candidate genes to facilitate genetic engineering efforts that increase cassava yield.

## Methods

### Plant material and steam girdling of the petiole of leaves in intact cassava plants

The stem cutting of cassava (*Manihotesculenta* Crantz.), Nigerian TME 7 variety (also known as Oko-iyawo) collected from farms in Nigeria, Africa were established and maintained under tissue culture conditions at Donald Danforth Plant Science Center, USA^38^. The nodes containing an axillary bud were excised from the stems within about 5 mm of each node and collected in a sterile flask. The nodes were then surface sterilized with 15% (v/v) sodium hypochlorite solution containing 2–4 drops of Tween 20 (Sigma Aldrich) for 30 minutes with constant shaking at 150 rpm followed by rinsing with sterile water for 3–4 times. The nodes containing axillary buds cultured on MS (Murashige and Skoog 1962) medium containing 20 g/l sucrose and 2.5 g/l phytogel after trimming off the dead and bleached tissues. The cultures were incubated at 28°C for 3 weeks under 16 h photo period at 75 μmol/m^−2^s^−1^. The shoots emerging from the nodes were excised and maintained by sub culturing every 4 to 6 weeks on fresh MS basal media. About 1 to 1.5 cm of shoots from the in vitro cultures were transferred to MS media containing 20 g/l sucrose and 2.5 g/l phytogel for 3 to 4 weeks. The plantlets with well-developed roots were transplanted to Fafard 51 growing mixture, Professional Formula (60–65% composted bark, Canadian sphagnum peatmoss, perlite, and dolomitic limestone) under a 14-h light/10-h dark photoperiod (250 to 500 μmol s^−1^ m^−2^ irradiance), 29°C day/25°C night temperature cycles with a relative humidity of ~50% in the greenhouse at the Donald Danforth Plant Science Center, USA.

The first fully expanded leaves of cassava plants at two developmental stages, 45 days (without storage roots) and 120 days (with storage roots), were subjected to steam-heating of the petioles at the end of the day. To control for heating stress, partial girdling (PG) by steam treatment on the upper side of the petiole, and full girdling (FG)—which involved treatment of the full circle of the petiole—were performed. The treated and non-treated controls of the first fully expanded leaves were collected at 0, 2, and 4 h after exposure to light on the morning of the following day. One leaf lobe from each treated leaf was cut for starch staining to examine treatment effectiveness; the others were immediately individually frozen in liquid nitrogen. A total of four leaves from each treatment were pooled, and two biological replicates were harvested. The same samples were also subjected to carbohydrate quantification and RNA-Seq sequencing.

### Net photosynthesis rate (Pn) and A-Ci curve determinations

Net photosynthesis (Pn) and CO_2_ assimilation rates in steam-treated greenhouse plants were measured the following day at 0, 2, and 4 h after exposure to light, as described above, using the Li-6400 portable photosystem unit at light saturating conditions, irradiance of 1200 μmol m^−2^ s^−1^ (Li-Cor Biosciences Inc., Lincoln, NB, USA). The A-Ci Curve was determined accordingly.

### Starch stain and carbohydrate quantification

To determine the effectiveness of heat girdling on blocking sucrose transportation in phloem, leaf lobes were subjected to iodine potassium iodide (IKI) staining to visualize starch accumulation according to Ruzin^39^. Treated leaves were pooled and ground into a fine powder in liquid nitrogen. Methods of extraction and quantification of sugars were modified according to Lisec^40^. Samples were run on a Thermo Scientific ISQ single quadrupole GC-MS system coupled with a DB5 silica column (30 m length, 0.25 mm diameter, 0.25 m film thickness) for sucrose, glucose, and fructose quantification. After extraction for GC/MS analysis, the tissue residue was re-extracted three times with 80% (v/v) ethanol at 80°C to remove any soluble sugars, and the pellet was dried down. The residue was digested to glucose equivalents using the total starch assay kit (Megazyme, Wickow, Ireland) and spectrophotometrically quantified at 510 nm. The experiment was repeated three times and statistical analysis was performed using the SPSS software.

### Total RNA, poly(A) RNA isolation, and RNA-Seq library construction

Total RNA from the leaf tissues was extracted using RNA plant reagent kits (Tiangen Company) and purified using the TURBO DNA-free™ Kit (Ambion). The integrity and quality of the total RNA were examined using a NanoDrop 1000 spectrophotometer and formaldehyde-agarose gel electrophoresis. The poly(A) RNA was isolated from purified total RNA using poly(T) oligonucleotide-attached magnetic beads (Invitrogen). Following purification, the mRNA was fragmented into small pieces using divalent cations under elevated temperatures, and the cleaved RNA fragments were reverse-transcribed into first-strand cDNA using reverse transcriptase and random primers. Second-strand cDNA synthesis was performed using DNA polymerase I and RNaseH, and the cDNA fragments were processed for end repair, a single “A” base was added, and sequences were ligated to the adapters. These products were then purified and enriched by PCR to create the final cDNA libraries and sequenced on the Illumina Hi-Seq 2500 with 51-bp lengths according to the manufacturer's recommendations (Illumina).

### Mapping of Illumina reads and data analysis

Adapters were removed from raw sequence reads using FASTX-toolkit pipeline, version 0.0.13 (http://hannonlab.cshl.edu/fastx_toolkit/). Sequence quality was examined using FastQC (http://www.bioinformatics.babraham.ac.uk/projects/fastqc/). Reads were mapped to the cassava genome (version 4.1) obtained from the phytozome website (ftp://ftp.jgi-psf.org/pub/compgen/phytozome/v9.0/Mesculenta/) using Tophat v. 2.0.10 (http://tophat.cbcb.umd.edu/)^41^. Differentially expressed genes (DEGs) were identified using Cuffdiff (http://cufflinks.cbcb.umd.edu/)^42^ based on a comparison of the treatment and control with FDR < 0.001.

### Pathway and clustering analysis

Gene expression changes (log_2_ fold change of treatment versus non-treated control) were imported into the MapMan and Pageman software (http://mapman.gabipd.org/web/guest) for pathway and/or functional category analysis^43^, and FDR from Pageman enrichment assays were controlled using Benjamini and Hochberg's procedure^44^. HCL (hierarchical clustering) clustering embedded in the MEV software (http://www.tm4.org/mev.html) was used to perform clustering analysis.

### Quantitative RT-PCR analysis

To verify the RNA-Seq results, quantitative RT-PCR was conducted using SYBR-green (TaKaRa Biotechnology Co., Ltd, Dalian, China) and ABI PRISM/TaqMan 7900 Sequence Detection System (Applied Biosystems), as described previously. We used the Primer Premier v. 5.0 and Vector NIT v.11.0 software (Applied Biosystems) to design primers based on the sequences of key genes of interest identified in our library. The cassava actin gene was used as an internal control. The sequences of primers used in this study are provided in Supplemental Table S1. qRT-PCR reactions were repeated four times for each sample and the relative mRNA level was calculated as 2^−ΔΔCT^. Correlation and significance analyses were performed using Microsoft Office Excel 2007.

## Author Contributions

P.L. and Y.Z. conceived the project, designed the studies and drafted the manuscript; Y.Z. and F.M. performed experiments. Y.Z. and Z.D. performed transcriptome analyses; R.C. performed tissue culture of cassava and provided seedlings; D.A., T.B., W.W., M.P. and P.L. edit the manuscript. All authors reviewed the manuscript.

## Supplementary Material

Supplementary Information Supplemental figures and tables

## Notes

The raw data of RNA-seq libraries was submitted to Sequence Read Archive with accession ID of PRJNA262651.
