# Supplementary material for: Transcriptional response to petiole heat girdling in cassava
Source: Sci Rep. 2015 Feb 12;5:8414. doi: 10.1038/srep08414 (PMC4325323; doi:10.1038/srep08414)
Supplement: Supplementary Information — Supplemental figures and tables [file srep08414-s1.pdf]

Supplemental Information

Title: Transcriptional response to petiole heat girdling in cassava

Authors:

Yang Zhang
The Institute of Tropical Bioscience and Biotechnology (ITBB)
Chinese Academy of Tropical Agricultural Sciences (CATAS)
Haikou, Hainan 571101, China

Zehong Ding (Co-first Author)
The Institute of Tropical Bioscience and Biotechnology (ITBB)
Chinese Academy of Tropical Agricultural Sciences (CATAS)
Haikou, Hainan 571101, China

Fangfang Ma
Donald Danforth Plant Science Center
975 North Warson Road, St. Louis, Missouri 63132

Raj Deepika Chauhan
Donald Danforth Plant Science Center
975 North Warson Road, St. Louis, Missouri 63132

Doug K. Allen
USDA-ARS/Donald Danforth Plant Science Center
975 North Warson Road, St. Louis, Missouri 63132
St. Louis, Missouri 63132, USA

Thomas P. Brutnell
Donald Danforth Plant Science Center
975 North Warson Road, St. Louis, Missouri 63132

Wenquan Wang
The Institute of Tropical Bioscience and Biotechnology (ITBB)
Chinese Academy of Tropical Agricultural Sciences (CATAS)
Haikou, Hainan 571101, China

Ming Peng
The Institute of Tropical Bioscience and Biotechnology (ITBB)
Chinese Academy of Tropical Agricultural Sciences (CATAS)
Haikou, Hainan 571101, China

Pinghua Li (Corresponding Author)
The Institute of Tropical Bioscience and Biotechnology (ITBB)
Chinese Academy of Tropical Agricultural Sciences (CATAS)
Haikou, Hainan 571101, China
Shandong Agricultural University
Tai'an, Shandong 271018, China

Email: Pinghuali@sdau.edu.cn

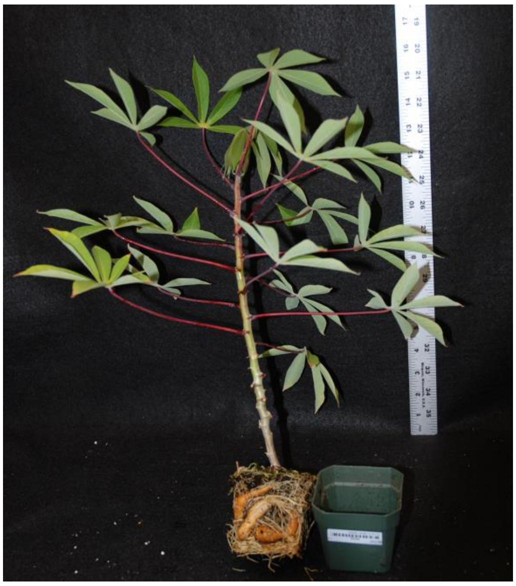

Supplemental Figure 1| Cassava plant with developing storage root in pot.

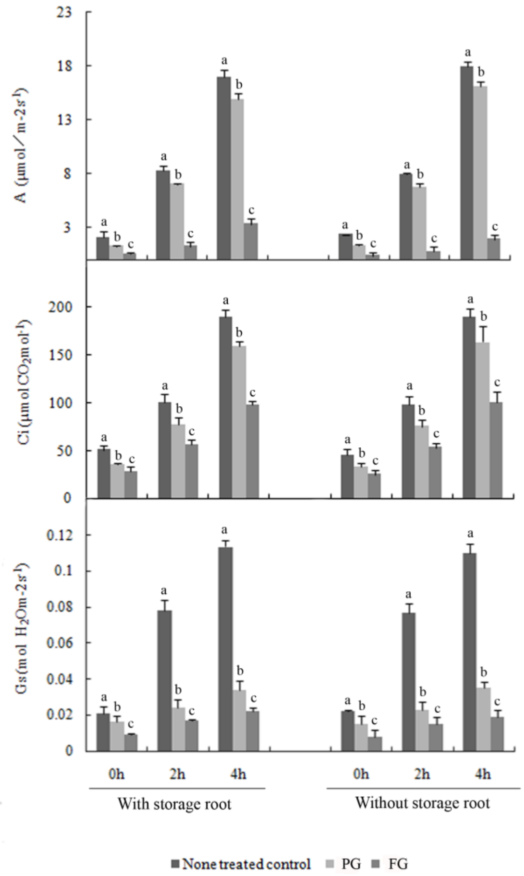

Supplemental Figure 2| Photosynthetic parameters of control, PG and FG Leaves of cassava plants. Measurements were made at an average ambient relative humidity of 46.6 ± 3.4% and an irradiance of 2000 µmol m^{-2} s^{-1}. A: net photosynthesis rates (Pn); Gs: stomata conductance, Ci: intercellular CO_2 concentration.

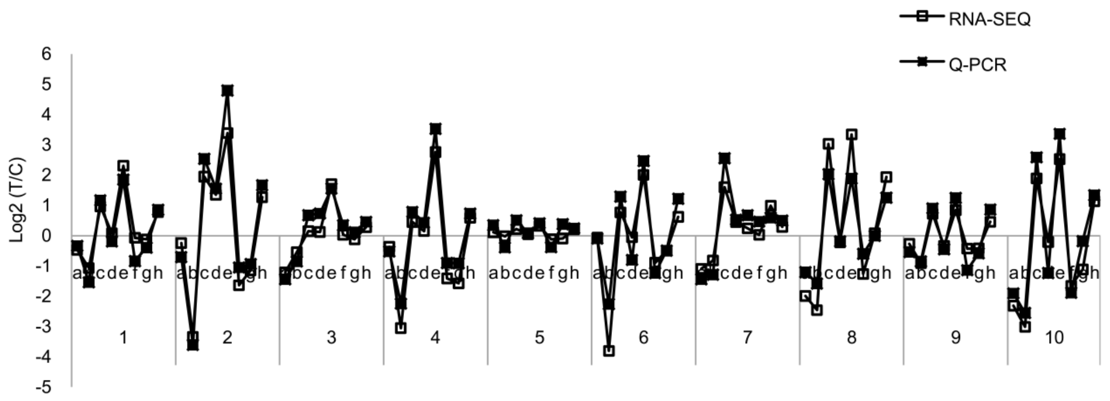

Supplemental Figure 3| Verification of microarray results by real-time quantitative RT-PCR. Log$_2$ (treat/control) values for genes (a–k) in the six experimental conditions. Genes: (a) cassava4.1_017330m.g ; (b) cassava4.1_008668m.g; (c) cassava4.1_001840m.g ; (d) cassava4.1_000732m.g ; (e) cassava4.1_005409m.g; (f) cassava4.1_002278m.g ; (g) cassava4.1_004345m.g ; (h) cassava4.1_006138m.g . Experimental conditions (1) 0-h PG leaves, (2) 0-h FG leaves, (3) 2-h PG leaves, (4) 2-h FG leaves, (5) 4-h PG leaves, (6) 4-h FG leaves; conditions 7–10 (left to right) lack storage roots: (7) 0-h PG leaves, (8) 0-h FG leaves, (9) 2-h PG leaves, (10) 2-h FG leaves. Primers are listed in Supplemental Table S1.

Supplemental Table S1. Glucose and fructose measurements for control, PG and FG leaves. All leaves were harvested and processed concurrently

		Glucose (mg/g FW)			Fructose (mg/g FW)		
		Control	PG	FG	Control	PG	FG
120day	0h	1.80±0.40	0.49±0.45	1.48±0.16	0.51±0.02	0.12±0.04	0.20±0.01
	2h	1.15±0.25	0.92±0.06	0.35±0.08	0.36±0.02	0.12±0.01	0.02±0.01
	4h	0.67±0.14	0.43±0.07	0.58±0.20	0.21±0.01	0.09±0.02	0.04±0.01
45 day	0h	3.78±0.42	2.08±0.16	3.00±0.24	0.21±0.04	0.08±0.02	0.41±0.03
	2h	3.91±0.38	3.47±0.21	2.00±0.29	0.29±0.04	0.19±0.01	0.09±0.04
	4h	3.73±0.37	2.66±0.25	3.86±0.34	0.19±0.01	0.11±0.01	0.31±0.03

Supplemental Table S2. Primer sequences used for Real-time PCR .

Maize ID	Class	Forward (5'-3')	Reverse (5'-3')
cassava4.1_017330m.g	rubisco	CTCATACCTTCCACCACTCTCA	TGGCAGCTTCCACATTGTC
cassava4.1_008668m.g	FBPase	GGAGTGTTTGCCTTCACC	CCACTTCCGTTTGTGAGG
cassava4.1_001840m.g	Susy	CATGGCTCCCTCGGAGAT	GTTTGCCAACCTTTGCTACC
cassava4.1_000732m.g	SPS	AGTGGGGCGGGTGCTATTGA	CTTCGCTCGCGGGTGTTG
cassava4.1_005409m.g	AGPase	CAGAGCCAAGGCAAAGGTCA	CCAGAGGTCATTAGCATGAGA
cassava4.1_002278m.g	starch synthase	GTGGAGAATTATTACTGGATGTG	TCCTTGAGCAGCCTTGAC
cassava4.1_004345m.g	beta amylase	CATCAGCCAAGGCCATCC	AGGTAACCGTCCCGAAAT
cassava4.1_006138m.g	hexokinase	GTAACTTTCGGTCCTCACA	GTAACTTTCGGTCCTCACA

Supplemental Table S3. GO term enrichment of DE genes.

GO.ID	GO_class	Term	Annotated	Significant	Expected	Fisher_pvalue	Group
GO:1901607	BP	alpha-amino acid biosynthetic process	63	20	10.48	0.00226	0FG_0CK
GO:1901607	BP	alpha-amino acid biosynthetic process	63	19	9.34	0.00142	120d_0FG_120d_0CK
GO:1901605	BP	alpha-amino acid metabolic process	96	30	15.97	0.00028	0FG_0CK
GO:1901605	BP	alpha-amino acid metabolic process	96	24	11.71	0.0004	120d_4FG_120d_4CK
GO:1901605	BP	alpha-amino acid metabolic process	96	27	14.23	0.00055	120d_0FG_120d_0CK
GO:1901605	BP	alpha-amino acid metabolic process	96	22	11.08	0.00114	120d_2FG_120d_2CK
GO:0042886	BP	amide transport	66	16	3.26	8.50E-08	120d_0PG_120d_0CK
GO:0042886	BP	amide transport	66	11	2.09	6.20E-06	120d_2PG_120d_2CK
GO:0043038	BP	amino acid activation	60	18	9.98	0.00712	0FG_0CK
GO:0043038	BP	amino acid activation	60	17	8.48	0.00308	2FG_2CK
GO:0009073	BP	aromatic amino acid family biosynthetic ...	18	9	2.54	0.00032	2FG_2CK
GO:0009073	BP	aromatic amino acid family biosynthetic ...	18	8	2.2	0.00068	120d_4FG_120d_4CK
GO:0009073	BP	aromatic amino acid family biosynthetic ...	18	9	2.08	6.50E-05	120d_2FG_120d_2CK
GO:0009072	BP	aromatic amino acid family metabolic pro...	24	10	3.39	0.00091	2FG_2CK
GO:0009072	BP	aromatic amino acid family metabolic pro...	24	10	2.93	0.00027	120d_4FG_120d_4CK
GO:0009072	BP	aromatic amino acid family metabolic pro...	24	10	3.56	0.00133	120d_0FG_120d_0CK
GO:0009072	BP	aromatic amino acid family metabolic pro...	24	10	2.77	0.00017	120d_2FG_120d_2CK
GO:0046034	BP	ATP metabolic process	51	14	6.22	0.00247	120d_4FG_120d_4CK
GO:0015986	BP	ATP synthesis coupled proton transport	23	10	2.81	0.00018	120d_4FG_120d_4CK
GO:0009058	BP	biosynthetic process	2697	518	448.52	2.50E-05	0FG_0CK
GO:0009058	BP	biosynthetic process	2697	430	381.01	0.00112	2FG_2CK
GO:0009058	BP	biosynthetic process	2697	395	328.96	6.30E-06	120d_4FG_120d_4CK
GO:0016052	BP	carbohydrate catabolic process	89	28	14.8	0.0004	0FG_0CK
GO:0016052	BP	carbohydrate catabolic process	89	29	12.57	7.20E-06	2FG_2CK
GO:0016052	BP	carbohydrate catabolic process	89	29	10.86	3.50E-07	120d_4FG_120d_4CK
GO:0016052	BP	carbohydrate catabolic process	89	29	13.19	1.80E-05	120d_0FG_120d_0CK
GO:0016052	BP	carbohydrate catabolic process	89	8	2.82	0.00711	120d_2PG_120d_2CK
GO:0016052	BP	carbohydrate catabolic process	89	30	10.27	2.70E-08	120d_2FG_120d_2CK
GO:0005975	BP	carbohydrate metabolic process	778	40	25.21	0.00239	0PG_0CK
GO:0005975	BP	carbohydrate metabolic process	778	139	109.91	0.00148	2FG_2CK
GO:0015977	BP	carbon fixation	10	6	1.66	0.0024	0FG_0CK
GO:0046394	BP	carboxylic acid biosynthetic process	194	49	32.26	0.00132	0FG_0CK
GO:0046394	BP	carboxylic acid biosynthetic process	194	41	27.41	0.0047	2FG_2CK
GO:0046394	BP	carboxylic acid biosynthetic process	194	44	23.66	2.80E-05	120d_4FG_120d_4CK
GO:0046394	BP	carboxylic acid biosynthetic process	194	55	28.75	7.60E-07	120d_0FG_120d_0CK
GO:0046394	BP	carboxylic acid biosynthetic process	194	44	22.39	7.00E-06	120d_2FG_120d_2CK
GO:0019752	BP	carboxylic acid metabolic process	361	94	60.04	2.70E-06	0FG_0CK
GO:0019752	BP	carboxylic acid metabolic process	361	82	51	5.40E-06	2FG_2CK
GO:0019752	BP	carboxylic acid metabolic process	361	78	44.03	2.20E-07	120d_4FG_120d_4CK
GO:0019752	BP	carboxylic acid metabolic process	361	90	53.5	2.10E-07	120d_0FG_120d_0CK
GO:0019752	BP	carboxylic acid metabolic process	361	71	41.67	3.70E-06	120d_2FG_120d_2CK

GO:0009056	BP	catabolic process	355	75	52.61	0.00073	120d_0FG_120d_0CK
GO:0008652	BP	cellular amino acid biosynthetic process	111	36	18.46	3.00E-05	0FG_0CK
GO:0008652	BP	cellular amino acid biosynthetic process	111	30	15.68	0.00025	2FG_2CK
GO:0008652	BP	cellular amino acid biosynthetic process	111	28	13.54	0.00012	120d_4FG_120d_4CK
GO:0008652	BP	cellular amino acid biosynthetic process	111	32	16.45	0.00011	120d_0FG_120d_0CK
GO:0008652	BP	cellular amino acid biosynthetic process	111	24	12.81	0.00165	120d_2FG_120d_2CK
GO:0006520	BP	cellular amino acid metabolic process	233	69	38.75	4.50E-07	0FG_0CK
GO:0006520	BP	cellular amino acid metabolic process	233	60	32.92	1.60E-06	2FG_2CK
GO:0006520	BP	cellular amino acid metabolic process	233	47	28.42	0.00031	120d_4FG_120d_4CK
GO:0006520	BP	cellular amino acid metabolic process	233	57	34.53	6.20E-05	120d_0FG_120d_0CK
GO:0006520	BP	cellular amino acid metabolic process	233	40	26.89	0.00637	120d_2FG_120d_2CK
GO:0044249	BP	cellular biosynthetic process	2498	460	415.42	0.00376	0FG_0CK
GO:0044249	BP	cellular biosynthetic process	2498	353	304.69	0.00052	120d_4FG_120d_4CK
GO:0044262	BP	cellular carbohydrate metabolic process	152	13	4.93	0.00132	0PG_0CK
GO:0044262	BP	cellular carbohydrate metabolic process	152	11	4.81	0.00894	120d_2PG_120d_2CK
GO:0044255	BP	cellular lipid metabolic process	210	39	24.24	0.00172	120d_2FG_120d_2CK
GO:0044264	BP	cellular polysaccharide metabolic proces...	91	8	2.95	0.00923	0PG_0CK
GO:0043623	BP	cellular protein complex assembly	40	6	1.3	0.00168	0PG_0CK
GO:0006732	BP	coenzyme metabolic process	74	7	2.34	0.0088	120d_2PG_120d_2CK
GO:0051188	BP	cofactor biosynthetic process	62	19	9.19	0.00115	120d_0FG_120d_0CK
GO:0051186	BP	cofactor metabolic process	92	29	15.3	0.0003	0FG_0CK
GO:0051186	BP	cofactor metabolic process	92	5	1.27	0.0089	2PG_2CK
GO:0051186	BP	cofactor metabolic process	92	25	13	0.00074	2FG_2CK
GO:0051186	BP	cofactor metabolic process	92	32	13.63	1.40E-06	120d_0FG_120d_0CK
GO:0051186	BP	cofactor metabolic process	92	8	2.91	0.00863	120d_2PG_120d_2CK
GO:0005984	BP	disaccharide metabolic process	37	13	6.15	0.00493	0FG_0CK
GO:0005984	BP	disaccharide metabolic process	37	12	5.23	0.00367	2FG_2CK
GO:0005984	BP	disaccharide metabolic process	37	6	1.83	0.00884	120d_0PG_120d_0CK
GO:0015985	BP	energy coupled proton transport, down el...	23	10	2.81	0.00018	120d_4FG_120d_4CK
GO:0006633	BP	fatty acid biosynthetic process	76	22	11.26	0.00114	120d_0FG_120d_0CK
GO:0006633	BP	fatty acid biosynthetic process	76	20	8.77	0.00029	120d_2FG_120d_2CK
GO:0006631	BP	fatty acid metabolic process	91	8	2.95	0.00923	0PG_0CK
GO:0006631	BP	fatty acid metabolic process	91	27	13.49	0.00021	120d_0FG_120d_0CK
GO:0006631	BP	fatty acid metabolic process	91	25	10.5	2.40E-05	120d_2FG_120d_2CK
GO:0006091	BP	generation of precursor metabolites and ...	99	35	16.46	4.60E-06	0FG_0CK
GO:0006091	BP	generation of precursor metabolites and ...	99	36	13.99	2.50E-08	2FG_2CK
GO:0006091	BP	generation of precursor metabolites and ...	99	32	12.08	1.10E-07	120d_4FG_120d_4CK
GO:0006091	BP	generation of precursor metabolites and ...	99	29	14.67	0.00016	120d_0FG_120d_0CK
GO:0006091	BP	generation of precursor metabolites and ...	99	35	11.43	4.30E-10	120d_2FG_120d_2CK
GO:0006007	BP	glucose catabolic process	76	25	12.64	0.00037	0FG_0CK
GO:0006007	BP	glucose catabolic process	76	26	10.74	7.90E-06	2FG_2CK
GO:0006007	BP	glucose catabolic process	76	27	9.27	1.20E-07	120d_4FG_120d_4CK
GO:0006007	BP	glucose catabolic process	76	23	11.26	0.00045	120d_0FG_120d_0CK
GO:0006007	BP	glucose catabolic process	76	25	8.77	6.50E-07	120d_2FG_120d_2CK

GO:0006006	BP	glucose metabolic process	86	30	14.3	2.90E-05	0FG_0CK
GO:0006006	BP	glucose metabolic process	86	29	12.15	3.30E-06	2FG_2CK
GO:0006006	BP	glucose metabolic process	86	30	10.49	3.90E-08	120d_4FG_120d_4CK
GO:0006006	BP	glucose metabolic process	86	25	12.74	0.00051	120d_0FG_120d_0CK
GO:0006006	BP	glucose metabolic process	86	27	9.93	6.70E-07	120d_2FG_120d_2CK
GO:0009084	BP	glutamine family amino acid biosynthetic...	12	6	1.7	0.00339	2FG_2CK
GO:0009084	BP	glutamine family amino acid biosynthetic...	12	6	1.46	0.00156	120d_4FG_120d_4CK
GO:0009064	BP	glutamine family amino acid metabolic pr...	21	8	2.97	0.00572	2FG_2CK
GO:0009064	BP	glutamine family amino acid metabolic pr...	21	8	2.56	0.00225	120d_4FG_120d_4CK
GO:0006096	BP	glycolysis	58	19	9.65	0.00192	0FG_0CK
GO:0006096	BP	glycolysis	58	19	8.19	0.00025	2FG_2CK
GO:0006096	BP	glycolysis	58	20	7.07	8.70E-06	120d_4FG_120d_4CK
GO:0006096	BP	glycolysis	58	19	6.69	1.50E-05	120d_2FG_120d_2CK
GO:0019320	BP	hexose catabolic process	76	25	12.64	0.00037	0FG_0CK
GO:0019320	BP	hexose catabolic process	76	26	10.74	7.90E-06	2FG_2CK
GO:0019320	BP	hexose catabolic process	76	27	9.27	1.20E-07	120d_4FG_120d_4CK
GO:0019320	BP	hexose catabolic process	76	23	11.26	0.00045	120d_0FG_120d_0CK
GO:0019320	BP	hexose catabolic process	76	25	8.77	6.50E-07	120d_2FG_120d_2CK
GO:0019318	BP	hexose metabolic process	105	34	17.46	5.20E-05	0FG_0CK
GO:0019318	BP	hexose metabolic process	105	35	14.83	4.60E-07	2FG_2CK
GO:0019318	BP	hexose metabolic process	105	32	12.81	4.90E-07	120d_4FG_120d_4CK
GO:0019318	BP	hexose metabolic process	105	29	15.56	0.00048	120d_0FG_120d_0CK
GO:0019318	BP	hexose metabolic process	105	28	12.12	1.50E-05	120d_2FG_120d_2CK
GO:1902600	BP	hydrogen ion transmembrane transport	60	16	7.32	0.00174	120d_4FG_120d_4CK
GO:0006818	BP	hydrogen transport	71	18	8.66	0.00173	120d_4FG_120d_4CK
GO:0008299	BP	isoprenoid biosynthetic process	33	12	4.66	0.00121	2FG_2CK
GO:0008299	BP	isoprenoid biosynthetic process	33	10	3.81	0.00309	120d_2FG_120d_2CK
GO:0006720	BP	isoprenoid metabolic process	33	12	4.66	0.00121	2FG_2CK
GO:0006720	BP	isoprenoid metabolic process	33	10	3.81	0.00309	120d_2FG_120d_2CK
GO:0008610	BP	lipid biosynthetic process	170	42	25.19	0.00044	120d_0FG_120d_0CK
GO:0008610	BP	lipid biosynthetic process	170	37	19.62	9.30E-05	120d_2FG_120d_2CK
GO:0006563	BP	L-serine metabolic process	17	11	2.83	1.20E-05	0FG_0CK
GO:0006563	BP	L-serine metabolic process	17	7	2.4	0.00589	2FG_2CK
GO:0006563	BP	L-serine metabolic process	17	5	0.84	0.00108	120d_0PG_120d_0CK
GO:0006563	BP	L-serine metabolic process	17	8	1.96	0.00029	120d_2FG_120d_2CK
GO:0008152	BP	metabolic process	8536	1481	1419.56	7.10E-05	0FG_0CK
GO:0008152	BP	metabolic process	8536	1268	1205.89	1.80E-05	2FG_2CK
GO:0008152	BP	metabolic process	8536	1102	1041.17	7.90E-06	120d_4FG_120d_4CK
GO:0008152	BP	metabolic process	8536	1330	1264.94	1.10E-05	120d_0FG_120d_0CK
GO:0008152	BP	metabolic process	8536	1046	985.22	4.80E-06	120d_2FG_120d_2CK
GO:0072330	BP	monocarboxylic acid biosynthetic process	83	23	12.3	0.0017	120d_0FG_120d_0CK
GO:0072330	BP	monocarboxylic acid biosynthetic process	83	20	9.58	0.00097	120d_2FG_120d_2CK
GO:0032787	BP	monocarboxylic acid metabolic process	102	28	15.12	0.00067	120d_0FG_120d_0CK
GO:0032787	BP	monocarboxylic acid metabolic process	102	25	11.77	0.00018	120d_2FG_120d_2CK

GO:0046365	BP	monosaccharide catabolic process	76	25	12.64	0.00037	0FG_0CK
GO:0046365	BP	monosaccharide catabolic process	76	26	10.74	7.90E-06	2FG_2CK
GO:0046365	BP	monosaccharide catabolic process	76	27	9.27	1.20E-07	120d_4FG_120d_4CK
GO:0046365	BP	monosaccharide catabolic process	76	23	11.26	0.00045	120d_0FG_120d_0CK
GO:0046365	BP	monosaccharide catabolic process	76	25	8.77	6.50E-07	120d_2FG_120d_2CK
GO:0005996	BP	monosaccharide metabolic process	106	34	17.63	6.40E-05	0FG_0CK
GO:0005996	BP	monosaccharide metabolic process	106	36	14.97	1.90E-07	2FG_2CK
GO:0005996	BP	monosaccharide metabolic process	106	32	12.93	6.20E-07	120d_4FG_120d_4CK
GO:0005996	BP	monosaccharide metabolic process	106	29	15.71	0.00057	120d_0FG_120d_0CK
GO:0005996	BP	monosaccharide metabolic process	106	28	12.23	1.80E-05	120d_2FG_120d_2CK
GO:0043086	BP	negative regulation of catalytic activit...	66	7	2.14	0.00533	0PG_0CK
GO:0044092	BP	negative regulation of molecular functio...	66	7	2.14	0.00533	0PG_0CK
GO:0046496	BP	nicotinamide nucleotide metabolic proces...	21	8	2.97	0.00572	2FG_2CK
GO:0071705	BP	nitrogen compound transport	103	16	5.08	4.10E-05	120d_0PG_120d_0CK
GO:0071705	BP	nitrogen compound transport	103	11	3.26	0.00041	120d_2PG_120d_2CK
GO:0033865	BP	nucleoside bisphosphate metabolic proces...	17	9	2.83	0.00065	0FG_0CK
GO:0006753	BP	nucleoside phosphate metabolic process	193	14	6.25	0.00407	0PG_0CK
GO:0009117	BP	nucleotide metabolic process	187	14	6.06	0.00306	0PG_0CK
GO:0006857	BP	oligopeptide transport	66	16	3.26	8.50E-08	120d_0PG_120d_0CK
GO:0006857	BP	oligopeptide transport	66	11	2.09	6.20E-06	120d_2PG_120d_2CK
GO:0006857	BP	oligopeptide transport	66	15	7.62	0.00718	120d_2FG_120d_2CK
GO:0016053	BP	organic acid biosynthetic process	194	49	32.26	0.00132	0FG_0CK
GO:0016053	BP	organic acid biosynthetic process	194	41	27.41	0.0047	2FG_2CK
GO:0016053	BP	organic acid biosynthetic process	194	44	23.66	2.80E-05	120d_4FG_120d_4CK
GO:0016053	BP	organic acid biosynthetic process	194	55	28.75	7.60E-07	120d_0FG_120d_0CK
GO:0016053	BP	organic acid biosynthetic process	194	44	22.39	7.00E-06	120d_2FG_120d_2CK
GO:0006082	BP	organic acid metabolic process	362	94	60.2	3.10E-06	0FG_0CK
GO:0006082	BP	organic acid metabolic process	362	83	51.14	3.20E-06	2FG_2CK
GO:0006082	BP	organic acid metabolic process	362	78	44.15	2.50E-07	120d_4FG_120d_4CK
GO:0006082	BP	organic acid metabolic process	362	91	53.64	1.20E-07	120d_0FG_120d_0CK
GO:0006082	BP	organic acid metabolic process	362	72	41.78	2.10E-06	120d_2FG_120d_2CK
GO:1901576	BP	organic substance biosynthetic process	2535	468	421.58	0.00281	0FG_0CK
GO:1901576	BP	organic substance biosynthetic process	2535	360	309.2	0.0003	120d_4FG_120d_4CK
GO:1901575	BP	organic substance catabolic process	326	73	48.31	0.00014	120d_0FG_120d_0CK
GO:1901575	BP	organic substance catabolic process	326	53	37.63	0.006	120d_2FG_120d_2CK
GO:1901566	BP	organonitrogen compound biosynthetic pro...	279	74	46.4	1.60E-05	0FG_0CK
GO:1901566	BP	organonitrogen compound biosynthetic pro...	279	65	39.41	2.20E-05	2FG_2CK
GO:1901566	BP	organonitrogen compound biosynthetic pro...	279	56	34.03	9.90E-05	120d_4FG_120d_4CK
GO:1901566	BP	organonitrogen compound biosynthetic pro...	279	67	41.34	2.80E-05	120d_0FG_120d_0CK
GO:1901564	BP	organonitrogen compound metabolic	576	138	95.79	2.30E-06	0FG_0CK

		proces...					
GO:1901564	BP	organonitrogen compound metabolic proces...	576	124	81.37	4.80E-07	2FG_2CK
GO:1901564	BP	organonitrogen compound metabolic proces...	576	98	70.26	0.00032	120d_4FG_120d_4CK
GO:1901564	BP	organonitrogen compound metabolic proces...	576	126	85.36	2.10E-06	120d_0FG_120d_0CK
GO:0055114	BP	oxidation-reduction process	1345	274	223.68	6.90E-05	0FG_0CK
GO:0055114	BP	oxidation-reduction process	1345	37	18.61	2.60E-05	2PG_2CK
GO:0055114	BP	oxidation-reduction process	1345	244	190.01	7.00E-06	2FG_2CK
GO:0055114	BP	oxidation-reduction process	1345	235	164.05	1.00E-09	120d_4FG_120d_4CK
GO:0055114	BP	oxidation-reduction process	1345	101	66.36	7.10E-06	120d_0PG_120d_0CK
GO:0055114	BP	oxidation-reduction process	1345	261	199.31	5.90E-07	120d_0FG_120d_0CK
GO:0055114	BP	oxidation-reduction process	1345	68	42.61	5.10E-05	120d_2PG_120d_2CK
GO:0055114	BP	oxidation-reduction process	1345	231	155.24	3.50E-11	120d_2FG_120d_2CK
GO:0043436	BP	oxoacid metabolic process	361	94	60.04	2.70E-06	0FG_0CK
GO:0043436	BP	oxoacid metabolic process	361	82	51	5.40E-06	2FG_2CK
GO:0043436	BP	oxoacid metabolic process	361	78	44.03	2.20E-07	120d_4FG_120d_4CK
GO:0043436	BP	oxoacid metabolic process	361	90	53.5	2.10E-07	120d_0FG_120d_0CK
GO:0043436	BP	oxoacid metabolic process	361	71	41.67	3.70E-06	120d_2FG_120d_2CK
GO:0015833	BP	peptide transport	66	16	3.26	8.50E-08	120d_0PG_120d_0CK
GO:0015833	BP	peptide transport	66	11	2.09	6.20E-06	120d_2PG_120d_2CK
GO:0015833	BP	peptide transport	66	15	7.62	0.00718	120d_2FG_120d_2CK
GO:0015979	BP	photosynthesis	72	48	11.97	2.90E-21	0FG_0CK
GO:0015979	BP	photosynthesis	72	6	1	0.00047	2PG_2CK
GO:0015979	BP	photosynthesis	72	51	10.17	8.20E-28	2FG_2CK
GO:0015979	BP	photosynthesis	72	47	8.78	3.90E-26	120d_4FG_120d_4CK
GO:0015979	BP	photosynthesis	72	51	10.67	8.20E-27	120d_0FG_120d_0CK
GO:0015979	BP	photosynthesis	72	48	8.31	2.20E-28	120d_2FG_120d_2CK
GO:0009765	BP	photosynthesis, light harvesting	12	9	2	1.30E-05	0FG_0CK
GO:0009765	BP	photosynthesis, light harvesting	12	9	1.7	3.20E-06	2FG_2CK
GO:0009765	BP	photosynthesis, light harvesting	12	9	1.46	9.10E-07	120d_4FG_120d_4CK
GO:0009765	BP	photosynthesis, light harvesting	12	10	1.78	2.50E-07	120d_0FG_120d_0CK
GO:0009765	BP	photosynthesis, light harvesting	12	10	1.39	2.20E-08	120d_2FG_120d_2CK
GO:0019684	BP	photosynthesis, light reaction	20	12	3.33	1.50E-05	0FG_0CK
GO:0019684	BP	photosynthesis, light reaction	20	13	2.83	2.50E-07	2FG_2CK
GO:0019684	BP	photosynthesis, light reaction	20	11	2.44	5.00E-06	120d_4FG_120d_4CK
GO:0019684	BP	photosynthesis, light reaction	20	12	2.96	4.30E-06	120d_0FG_120d_0CK
GO:0019684	BP	photosynthesis, light reaction	20	12	2.31	2.80E-07	120d_2FG_120d_2CK
GO:0046148	BP	pigment biosynthetic process	14	7	2.33	0.00403	0FG_0CK
GO:0046148	BP	pigment biosynthetic process	14	7	1.98	0.00153	2FG_2CK
GO:0046148	BP	pigment biosynthetic process	14	9	1.71	6.60E-06	120d_4FG_120d_4CK
GO:0046148	BP	pigment biosynthetic process	14	10	2.07	2.80E-06	120d_0FG_120d_0CK
GO:0046148	BP	pigment biosynthetic process	14	7	1.62	0.00044	120d_2FG_120d_2CK

GO:0042440	BP	pigment metabolic process	19	9	3.16	0.00179	0FG_0CK
GO:0042440	BP	pigment metabolic process	19	9	2.68	0.00053	2FG_2CK
GO:0042440	BP	pigment metabolic process	19	10	2.32	2.30E-05	120d_4FG_120d_4CK
GO:0042440	BP	pigment metabolic process	19	12	2.82	2.00E-06	120d_0FG_120d_0CK
GO:0042440	BP	pigment metabolic process	19	9	2.19	0.00011	120d_2FG_120d_2CK
GO:0006779	BP	porphyrin-containing compound biosynthet...	11	6	1.34	0.00087	120d_4FG_120d_4CK
GO:0006779	BP	porphyrin-containing compound biosynthet...	11	8	1.63	2.50E-05	120d_0FG_120d_0CK
GO:0006779	BP	porphyrin-containing compound biosynthet...	11	5	1.27	0.00516	120d_2FG_120d_2CK
GO:0006778	BP	porphyrin-containing compound metabolic ...	16	7	1.95	0.00166	120d_4FG_120d_4CK
GO:0006778	BP	porphyrin-containing compound metabolic ...	16	10	2.37	1.70E-05	120d_0FG_120d_0CK
GO:0006778	BP	porphyrin-containing compound metabolic ...	16	7	1.85	0.00119	120d_2FG_120d_2CK
GO:0006457	BP	protein folding	131	12	4.25	0.00111	0PG_0CK
GO:0051258	BP	protein polymerization	33	6	1.07	0.00059	0PG_0CK
GO:0015992	BP	proton transport	71	18	8.66	0.00173	120d_4FG_120d_4CK
GO:0034032	BP	purine nucleoside bisphosphate metabolic...	17	9	2.83	0.00065	0FG_0CK
GO:0019362	BP	pyridine nucleotide metabolic process	21	8	2.97	0.00572	2FG_2CK
GO:0019362	BP	pyridine nucleotide metabolic process	21	9	3.11	0.00183	120d_0FG_120d_0CK
GO:0072524	BP	pyridine-containing compound metabolic p...	27	10	3.81	0.00262	2FG_2CK
GO:0072524	BP	pyridine-containing compound metabolic p...	27	11	4	0.00096	120d_0FG_120d_0CK
GO:0033875	BP	ribonucleoside bisphosphate metabolic pr...	17	9	2.83	0.00065	0FG_0CK
GO:0009070	BP	serine family amino acid biosynthetic pr...	10	6	1.66	0.0024	0FG_0CK
GO:0009070	BP	serine family amino acid biosynthetic pr...	10	6	1.48	0.00129	120d_0FG_120d_0CK
GO:0009069	BP	serine family amino acid metabolic proce...	26	14	4.32	1.60E-05	0FG_0CK
GO:0009069	BP	serine family amino acid metabolic proce...	26	6	1.28	0.00139	120d_0PG_120d_0CK
GO:0009069	BP	serine family amino acid metabolic proce...	26	10	3	0.00038	120d_2FG_120d_2CK
GO:0044711	BP	single-organism biosynthetic process	514	106	72.61	2.40E-05	2FG_2CK
GO:0044711	BP	single-organism biosynthetic process	514	92	62.69	7.80E-05	120d_4FG_120d_4CK
GO:0044711	BP	single-organism biosynthetic process	514	114	76.17	3.30E-06	120d_0FG_120d_0CK
GO:0044711	BP	single-organism biosynthetic process	514	92	59.33	9.10E-06	120d_2FG_120d_2CK
GO:0044724	BP	single-organism carbohydrate catabolic p...	79	25	13.14	0.00072	0FG_0CK
GO:0044724	BP	single-organism carbohydrate catabolic p...	79	26	11.16	1.70E-05	2FG_2CK
GO:0044724	BP	single-organism carbohydrate catabolic p...	79	29	9.64	1.80E-08	120d_4FG_120d_4CK
GO:0044724	BP	single-organism carbohydrate catabolic p...	79	25	11.71	0.00012	120d_0FG_120d_0CK
GO:0044724	BP	single-organism carbohydrate catabolic p...	79	8	2.5	0.00344	120d_2PG_120d_2CK
GO:0044724	BP	single-organism carbohydrate catabolic p...	79	27	9.12	9.50E-08	120d_2FG_120d_2CK
GO:0044723	BP	single-organism carbohydrate metabolic p...	290	73	40.97	3.20E-07	2FG_2CK

GO:0044723	BP	single-organism carbohydrate metabolic p...	290	53	33.47	0.00043	120d_2FG_120d_2CK
GO:0044712	BP	single-organism catabolic process	168	39	20.49	4.60E-05	120d_4FG_120d_4CK
GO:0044712	BP	single-organism catabolic process	168	43	24.9	0.00017	120d_0FG_120d_0CK
GO:0044712	BP	single-organism catabolic process	168	12	5.32	0.00715	120d_2PG_120d_2CK
GO:0044712	BP	single-organism catabolic process	168	34	19.39	0.00072	120d_2FG_120d_2CK
GO:0044710	BP	single-organism metabolic process	2620	508	435.71	1.00E-05	0FG_0CK
GO:0044710	BP	single-organism metabolic process	2620	467	370.13	6.70E-10	2FG_2CK
GO:0044710	BP	single-organism metabolic process	2620	414	319.57	1.80E-10	120d_4FG_120d_4CK
GO:0044710	BP	single-organism metabolic process	2620	165	129.26	0.00019	120d_0PG_120d_0CK
GO:0044710	BP	single-organism metabolic process	2620	486	388.25	9.60E-10	120d_0FG_120d_0CK
GO:0044710	BP	single-organism metabolic process	2620	108	82.99	0.00113	120d_2PG_120d_2CK
GO:0044710	BP	single-organism metabolic process	2620	406	302.4	1.10E-12	120d_2FG_120d_2CK
GO:0044699	BP	single-organism process	4479	694	632.75	0.00037	2FG_2CK
GO:0044699	BP	single-organism process	4479	597	546.32	0.0015	120d_4FG_120d_4CK
GO:0044699	BP	single-organism process	4479	259	220.97	0.00041	120d_0PG_120d_0CK
GO:0044699	BP	single-organism process	4479	739	663.74	2.40E-05	120d_0FG_120d_0CK
GO:0044699	BP	single-organism process	4479	169	141.88	0.00168	120d_2PG_120d_2CK
GO:0044699	BP	single-organism process	4479	581	516.96	6.10E-05	120d_2FG_120d_2CK
GO:0044283	BP	small molecule biosynthetic process	220	53	36.59	0.00264	0FG_0CK
GO:0044283	BP	small molecule biosynthetic process	220	45	26.83	0.0003	120d_4FG_120d_4CK
GO:0044283	BP	small molecule biosynthetic process	220	59	32.6	2.30E-06	120d_0FG_120d_0CK
GO:0044283	BP	small molecule biosynthetic process	220	47	25.39	1.80E-05	120d_2FG_120d_2CK
GO:0044282	BP	small molecule catabolic process	19	5	0.6	0.00025	120d_2PG_120d_2CK
GO:0044281	BP	small molecule metabolic process	665	155	110.59	3.00E-06	0FG_0CK
GO:0044281	BP	small molecule metabolic process	665	138	93.95	9.60E-07	2FG_2CK
GO:0044281	BP	small molecule metabolic process	665	130	81.11	1.40E-08	120d_4FG_120d_4CK
GO:0044281	BP	small molecule metabolic process	665	147	98.55	1.40E-07	120d_0FG_120d_0CK
GO:0044281	BP	small molecule metabolic process	665	111	76.75	2.80E-05	120d_2FG_120d_2CK
GO:0006814	BP	sodium ion transport	10	3	0.32	0.00342	0PG_0CK
GO:0006694	BP	steroid biosynthetic process	13	4	0.41	0.00056	120d_2PG_120d_2CK
GO:0008202	BP	steroid metabolic process	14	4	0.44	0.00077	120d_2PG_120d_2CK
GO:0005985	BP	sucrose metabolic process	12	3	0.39	0.00597	0PG_0CK
GO:0005985	BP	sucrose metabolic process	12	7	2	0.00127	0FG_0CK
GO:0005985	BP	sucrose metabolic process	12	6	1.7	0.00339	2FG_2CK
GO:0033014	BP	tetrapyrrole biosynthetic process	23	11	3.41	0.00018	120d_0FG_120d_0CK
GO:0033013	BP	tetrapyrrole metabolic process	28	11	4.66	0.00355	0FG_0CK
GO:0033013	BP	tetrapyrrole metabolic process	28	13	4.15	6.70E-05	120d_0FG_120d_0CK
GO:0033013	BP	tetrapyrrole metabolic process	28	9	3.23	0.00316	120d_2FG_120d_2CK
GO:0006412	BP	translation	493	178	81.99	6.90E-27	0FG_0CK
GO:0006412	BP	translation	493	141	69.65	9.40E-18	2FG_2CK
GO:0006412	BP	translation	493	150	60.13	1.90E-28	120d_4FG_120d_4CK
GO:0006414	BP	translational elongation	26	13	4.32	8.70E-05	0FG_0CK
GO:0006414	BP	translational elongation	26	11	3.17	0.00011	120d_4FG_120d_4CK
GO:0055085	BP	transmembrane transport	715	50	35.27	0.00749	120d_0PG_120d_0CK

GO:0043039	BP	tRNA aminoacylation	60	18	9.98	0.00712	0FG_0CK
GO:0043039	BP	tRNA aminoacylation	60	17	8.48	0.00308	2FG_2CK
GO:0006418	BP	tRNA aminoacylation for protein translat...	57	18	9.48	0.00392	0FG_0CK
GO:0006418	BP	tRNA aminoacylation for protein translat...	57	17	8.05	0.00168	2FG_2CK
GO:0071944	CC	cell periphery	202	14	5.21	0.00061	0PG_0CK
GO:0071944	CC	cell periphery	202	13	5.74	0.0045	120d_2PG_120d_2CK
GO:0005618	CC	cell wall	130	12	3.36	0.00011	0PG_0CK
GO:0009507	CC	chloroplast	23	15	3.92	3.30E-07	0FG_0CK
GO:0009507	CC	chloroplast	23	13	3.31	2.90E-06	2FG_2CK
GO:0009507	CC	chloroplast	23	10	2.93	0.00025	120d_4FG_120d_4CK
GO:0009507	CC	chloroplast	23	13	3.09	1.30E-06	120d_0FG_120d_0CK
GO:0009507	CC	chloroplast	23	10	2.43	5.10E-05	120d_2FG_120d_2CK
GO:0005737	CC	cytoplasm	908	254	154.71	8.30E-21	0FG_0CK
GO:0005737	CC	cytoplasm	908	207	130.68	1.10E-14	2FG_2CK
GO:0005737	CC	cytoplasm	908	208	115.78	4.80E-22	120d_4FG_120d_4CK
GO:0044444	CC	cytoplasmic part	720	208	122.68	2.40E-18	0FG_0CK
GO:0044444	CC	cytoplasmic part	720	172	103.62	6.00E-14	2FG_2CK
GO:0044444	CC	cytoplasmic part	720	186	91.81	4.50E-26	120d_4FG_120d_4CK
GO:0030312	CC	external encapsulating structure	145	12	3.74	0.00031	0PG_0CK
GO:0030312	CC	external encapsulating structure	145	10	4.12	0.0078	120d_2PG_120d_2CK
GO:0019898	CC	extrinsic component of membrane	16	11	2.73	6.30E-06	0FG_0CK
GO:0019898	CC	extrinsic component of membrane	16	11	2.3	1.10E-06	2FG_2CK
GO:0019898	CC	extrinsic component of membrane	16	11	2.04	3.20E-07	120d_4FG_120d_4CK
GO:0019898	CC	extrinsic component of membrane	16	14	2.15	5.00E-11	120d_0FG_120d_0CK
GO:0019898	CC	extrinsic component of membrane	16	11	1.69	4.40E-08	120d_2FG_120d_2CK
GO:0016021	CC	integral component of membrane	837	53	33.32	0.00019	120d_0PG_120d_0CK
GO:0016021	CC	integral component of membrane	837	137	112.41	0.00399	120d_0FG_120d_0CK
GO:0005622	CC	intracellular	2596	479	442.32	0.00197	0FG_0CK
GO:0043232	CC	intracellular non-membrane-bounded organ...	528	159	89.96	1.40E-15	0FG_0CK
GO:0043232	CC	intracellular non-membrane-bounded organ...	528	124	75.99	1.80E-09	2FG_2CK
GO:0043232	CC	intracellular non-membrane-bounded organ...	528	139	67.33	1.10E-19	120d_4FG_120d_4CK
GO:0043229	CC	intracellular organelle	1450	281	247.06	0.00251	0FG_0CK
GO:0043229	CC	intracellular organelle	1450	222	184.9	0.00029	120d_4FG_120d_4CK
GO:0044424	CC	intracellular part	1942	397	330.89	1.00E-07	0FG_0CK
GO:0044424	CC	intracellular part	1942	325	279.49	6.70E-05	2FG_2CK
GO:0044424	CC	intracellular part	1942	301	247.63	1.20E-06	120d_4FG_120d_4CK
GO:0031224	CC	intrinsic component of membrane	877	54	34.91	0.00033	120d_0PG_120d_0CK
GO:0031224	CC	intrinsic component of membrane	877	141	117.78	0.00688	120d_0FG_120d_0CK
GO:0032991	CC	macromolecular complex	994	245	169.36	2.20E-12	0FG_0CK
GO:0032991	CC	macromolecular complex	994	205	143.06	6.70E-10	2FG_2CK
GO:0032991	CC	macromolecular complex	994	214	126.75	4.50E-19	120d_4FG_120d_4CK

GO:0016020	CC	membrane	2087	106	83.08	0.00034	120d_0PG_120d_0CK
GO:0016020	CC	membrane	2087	339	280.28	2.10E-07	120d_0FG_120d_0CK
GO:0016020	CC	membrane	2087	74	59.34	0.0058	120d_2PG_120d_2CK
GO:0016020	CC	membrane	2087	261	220.48	5.70E-05	120d_2FG_120d_2CK
GO:0044425	CC	membrane part	1078	57	42.91	0.0091	120d_0PG_120d_0CK
GO:0044425	CC	membrane part	1078	186	144.77	2.40E-05	120d_0FG_120d_0CK
GO:0044425	CC	membrane part	1078	145	113.88	0.00034	120d_2FG_120d_2CK
GO:0005740	CC	mitochondrial envelope	66	17	8.42	0.00296	120d_4FG_120d_4CK
GO:0031966	CC	mitochondrial membrane	53	14	6.76	0.00528	120d_4FG_120d_4CK
GO:0044455	CC	mitochondrial membrane part	21	8	2.68	0.00296	120d_4FG_120d_4CK
GO:0044429	CC	mitochondrial part	74	19	9.44	0.00179	120d_4FG_120d_4CK
GO:0005739	CC	mitochondrion	84	22	10.71	0.0006	120d_4FG_120d_4CK
GO:0043228	CC	non-membrane-bounded organelle	528	159	89.96	1.40E-15	0FG_0CK
GO:0043228	CC	non-membrane-bounded organelle	528	124	75.99	1.80E-09	2FG_2CK
GO:0043228	CC	non-membrane-bounded organelle	528	139	67.33	1.10E-19	120d_4FG_120d_4CK
GO:0043226	CC	organelle	1450	281	247.06	0.00251	0FG_0CK
GO:0043226	CC	organelle	1450	222	184.9	0.00029	120d_4FG_120d_4CK
GO:1990204	CC	oxidoreductase complex	28	13	4.77	0.00028	0FG_0CK
GO:1990204	CC	oxidoreductase complex	28	16	4.03	1.60E-07	2FG_2CK
GO:1990204	CC	oxidoreductase complex	28	15	3.57	2.40E-07	120d_4FG_120d_4CK
GO:1990204	CC	oxidoreductase complex	28	18	3.76	5.70E-10	120d_0FG_120d_0CK
GO:1990204	CC	oxidoreductase complex	28	16	2.96	1.70E-09	120d_2FG_120d_2CK
GO:0034357	CC	photosynthetic membrane	52	34	8.86	7.50E-15	0FG_0CK
GO:0034357	CC	photosynthetic membrane	52	5	0.61	0.00032	2PG_2CK
GO:0034357	CC	photosynthetic membrane	52	37	7.48	1.50E-20	2FG_2CK
GO:0034357	CC	photosynthetic membrane	52	34	6.63	7.40E-19	120d_4FG_120d_4CK
GO:0034357	CC	photosynthetic membrane	52	36	6.98	2.00E-20	120d_0FG_120d_0CK
GO:0034357	CC	photosynthetic membrane	52	33	5.49	2.50E-20	120d_2FG_120d_2CK
GO:0009521	CC	photosystem	49	32	8.35	5.00E-14	0FG_0CK
GO:0009521	CC	photosystem	49	5	0.58	0.00024	2PG_2CK
GO:0009521	CC	photosystem	49	35	7.05	1.40E-19	2FG_2CK
GO:0009521	CC	photosystem	49	32	6.25	8.60E-18	120d_4FG_120d_4CK
GO:0009521	CC	photosystem	49	34	6.58	2.20E-19	120d_0FG_120d_0CK
GO:0009521	CC	photosystem	49	32	5.18	2.60E-20	120d_2FG_120d_2CK
GO:0009522	CC	photosystem I	16	12	2.73	5.20E-07	0FG_0CK
GO:0009522	CC	photosystem I	16	3	0.19	0.00078	2PG_2CK
GO:0009522	CC	photosystem I	16	13	2.3	3.80E-09	2FG_2CK
GO:0009522	CC	photosystem I	16	10	2.04	4.10E-06	120d_4FG_120d_4CK
GO:0009522	CC	photosystem I	16	10	2.15	6.70E-06	120d_0FG_120d_0CK
GO:0009522	CC	photosystem I	16	10	1.69	7.00E-07	120d_2FG_120d_2CK
GO:0009523	CC	photosystem II	30	20	5.11	1.90E-09	0FG_0CK
GO:0009523	CC	photosystem II	30	22	4.32	4.10E-13	2FG_2CK
GO:0009523	CC	photosystem II	30	22	3.83	3.20E-14	120d_4FG_120d_4CK
GO:0009523	CC	photosystem II	30	24	4.03	2.10E-16	120d_0FG_120d_0CK

GO:0009523	CC	photosystem II	30	22	3.17	5.60E-16	120d_2FG_120d_2CK
GO:0009654	CC	photosystem II oxygen evolving complex	20	12	3.41	1.80E-05	0FG_0CK
GO:0009654	CC	photosystem II oxygen evolving complex	20	14	2.88	2.40E-08	2FG_2CK
GO:0009654	CC	photosystem II oxygen evolving complex	20	14	2.55	4.80E-09	120d_4FG_120d_4CK
GO:0009654	CC	photosystem II oxygen evolving complex	20	16	2.69	2.70E-11	120d_0FG_120d_0CK
GO:0009654	CC	photosystem II oxygen evolving complex	20	14	2.11	3.80E-10	120d_2FG_120d_2CK
GO:0009536	CC	plastid	30	18	5.11	1.40E-07	0FG_0CK
GO:0009536	CC	plastid	30	16	4.32	5.80E-07	2FG_2CK
GO:0009536	CC	plastid	30	11	3.83	0.00073	120d_4FG_120d_4CK
GO:0009536	CC	plastid	30	5	1.19	0.00599	120d_0PG_120d_0CK
GO:0009536	CC	plastid	30	16	4.03	2.20E-07	120d_0FG_120d_0CK
GO:0009536	CC	plastid	30	4	0.85	0.0096	120d_2PG_120d_2CK
GO:0009536	CC	plastid	30	13	3.17	3.80E-06	120d_2FG_120d_2CK
GO:0000502	CC	proteasome complex	33	16	4.43	1.20E-06	120d_0FG_120d_0CK
GO:0005839	CC	proteasome core complex	29	16	3.89	1.20E-07	120d_0FG_120d_0CK
GO:0005839	CC	proteasome core complex	29	8	3.06	0.00845	120d_2FG_120d_2CK
GO:0019773	CC	proteasome core complex, alpha-subunit c...	15	8	2.01	0.00027	120d_0FG_120d_0CK
GO:0045259	CC	proton-transporting ATP synthase complex	23	10	2.93	0.00025	120d_4FG_120d_4CK
GO:0045261	CC	proton-transporting ATP synthase complex...	15	6	1.91	0.00758	120d_4FG_120d_4CK
GO:0016469	CC	proton-transporting two-sector ATPase co...	64	18	8.16	0.00075	120d_4FG_120d_4CK
GO:0030529	CC	ribonucleoprotein complex	366	141	62.36	6.60E-25	0FG_0CK
GO:0030529	CC	ribonucleoprotein complex	366	113	52.67	2.20E-17	2FG_2CK
GO:0030529	CC	ribonucleoprotein complex	366	132	46.67	< 1e-30	120d_4FG_120d_4CK
GO:0044391	CC	ribosomal subunit	21	9	3.58	0.00484	0FG_0CK
GO:0044391	CC	ribosomal subunit	21	8	3.02	0.0063	2FG_2CK
GO:0005840	CC	ribosome	342	140	58.27	6.50E-28	0FG_0CK
GO:0005840	CC	ribosome	342	111	49.22	6.20E-19	2FG_2CK
GO:0005840	CC	ribosome	342	131	43.61	< 1e-30	120d_4FG_120d_4CK
GO:0009579	CC	thylakoid	54	36	9.2	4.60E-16	0FG_0CK
GO:0009579	CC	thylakoid	54	5	0.64	0.00039	2PG_2CK
GO:0009579	CC	thylakoid	54	39	7.77	5.50E-22	2FG_2CK
GO:0009579	CC	thylakoid	54	36	6.89	2.50E-20	120d_4FG_120d_4CK
GO:0009579	CC	thylakoid	54	38	7.25	6.80E-22	120d_0FG_120d_0CK
GO:0009579	CC	thylakoid	54	35	5.7	5.90E-22	120d_2FG_120d_2CK
GO:0042651	CC	thylakoid membrane	24	15	4.09	7.50E-07	0FG_0CK
GO:0042651	CC	thylakoid membrane	24	17	3.45	5.20E-10	2FG_2CK
GO:0042651	CC	thylakoid membrane	24	17	3.06	7.30E-11	120d_4FG_120d_4CK
GO:0042651	CC	thylakoid membrane	24	19	3.22	4.70E-13	120d_0FG_120d_0CK
GO:0042651	CC	thylakoid membrane	24	16	2.54	6.30E-11	120d_2FG_120d_2CK
GO:0044436	CC	thylakoid part	54	36	9.2	4.60E-16	0FG_0CK
GO:0044436	CC	thylakoid part	54	5	0.64	0.00039	2PG_2CK
GO:0044436	CC	thylakoid part	54	39	7.77	5.50E-22	2FG_2CK
GO:0044436	CC	thylakoid part	54	36	6.89	2.50E-20	120d_4FG_120d_4CK

GO:0044436	CC	thylakoid part	54	38	7.25	6.80E-22	120d_0FG_120d_0CK
GO:0044436	CC	thylakoid part	54	35	5.7	5.90E-22	120d_2FG_120d_2CK
GO:0051537	MF	2 iron, 2 sulfur cluster binding	17	7	2.5	0.00737	0FG_0CK
GO:0051537	MF	2 iron, 2 sulfur cluster binding	17	8	2.08	0.00043	2FG_2CK
GO:0051537	MF	2 iron, 2 sulfur cluster binding	17	9	1.76	1.50E-05	120d_4FG_120d_4CK
GO:0051537	MF	2 iron, 2 sulfur cluster binding	17	6	1.69	0.00445	120d_2FG_120d_2CK
GO:0016832	MF	aldehyde-lyase activity	13	7	1.91	0.00111	0FG_0CK
GO:0016832	MF	aldehyde-lyase activity	13	7	1.59	0.00035	2FG_2CK
GO:0016832	MF	aldehyde-lyase activity	13	7	1.35	0.00012	120d_4FG_120d_4CK
GO:0016832	MF	aldehyde-lyase activity	13	4	0.55	0.00163	120d_0PG_120d_0CK
GO:0016832	MF	aldehyde-lyase activity	13	7	1.67	0.00048	120d_0FG_120d_0CK
GO:0016832	MF	aldehyde-lyase activity	13	7	1.29	9.30E-05	120d_2FG_120d_2CK
GO:0031420	MF	alkali metal ion binding	17	6	1.76	0.00558	120d_4FG_120d_4CK
GO:0031420	MF	alkali metal ion binding	17	6	1.69	0.00445	120d_2FG_120d_2CK
GO:0003905	MF	alkylbase DNA N-glycosylase activity	10	3	0.29	0.00258	0PG_0CK
GO:0016597	MF	amino acid binding	39	7	1.64	0.00106	120d_0PG_120d_0CK
GO:0016597	MF	amino acid binding	39	10	3.87	0.00388	120d_2FG_120d_2CK
GO:0004812	MF	aminoacyl-tRNA ligase activity	59	18	8.67	0.0015	0FG_0CK
GO:0004812	MF	aminoacyl-tRNA ligase activity	59	17	7.2	0.00049	2FG_2CK
GO:0016209	MF	antioxidant activity	160	14	6.72	0.00758	120d_0PG_120d_0CK
GO:0016887	MF	ATPase activity	360	62	46.19	0.00913	120d_0FG_120d_0CK
GO:0043492	MF	ATPase activity, coupled to movement of ...	97	24	14.26	0.00618	0FG_0CK
GO:0043492	MF	ATPase activity, coupled to movement of ...	97	21	11.84	0.00601	2FG_2CK
GO:0043492	MF	ATPase activity, coupled to movement of ...	97	22	12.45	0.00505	120d_0FG_120d_0CK
GO:0042626	MF	ATPase activity, coupled to transmembran...	97	24	14.26	0.00618	0FG_0CK
GO:0042626	MF	ATPase activity, coupled to transmembran...	97	21	11.84	0.00601	2FG_2CK
GO:0044769	MF	ATPase activity, coupled to transmembran...	28	8	2.91	0.00615	120d_4FG_120d_4CK
GO:0042626	MF	ATPase activity, coupled to transmembran...	97	22	12.45	0.00505	120d_0FG_120d_0CK
GO:0005509	MF	calcium ion binding	56	17	8.23	0.00213	0FG_0CK
GO:0005509	MF	calcium ion binding	56	19	6.84	1.90E-05	2FG_2CK
GO:0005509	MF	calcium ion binding	56	17	5.81	3.30E-05	120d_4FG_120d_4CK
GO:0005509	MF	calcium ion binding	56	18	7.19	0.00014	120d_0FG_120d_0CK
GO:0005509	MF	calcium ion binding	56	16	5.55	7.20E-05	120d_2FG_120d_2CK
GO:0019200	MF	carbohydrate kinase activity	12	6	1.76	0.00417	0FG_0CK
GO:0019200	MF	carbohydrate kinase activity	12	5	1.25	0.00507	120d_4FG_120d_4CK
GO:0016830	MF	carbon-carbon lyase activity	81	30	11.91	5.70E-07	0FG_0CK
GO:0016830	MF	carbon-carbon lyase activity	81	28	9.89	1.40E-07	2FG_2CK
GO:0016830	MF	carbon-carbon lyase activity	81	19	8.41	0.00051	120d_4FG_120d_4CK
GO:0016830	MF	carbon-carbon lyase activity	81	25	10.39	1.70E-05	120d_0FG_120d_0CK
GO:0016830	MF	carbon-carbon lyase activity	81	21	8.03	2.90E-05	120d_2FG_120d_2CK
GO:0016840	MF	carbon-nitrogen lyase activity	22	7	2.28	0.00536	120d_4FG_120d_4CK
GO:0016840	MF	carbon-nitrogen lyase activity	22	5	0.92	0.00187	120d_0PG_120d_0CK
GO:0031406	MF	carboxylic acid binding	39	7	1.64	0.00106	120d_0PG_120d_0CK
GO:0031406	MF	carboxylic acid binding	39	10	3.87	0.00388	120d_2FG_120d_2CK

GO:0016831	MF	carboxy-lyase activity	48	14	7.06	0.00751	0FG_0CK
GO:0016831	MF	carboxy-lyase activity	48	13	5.86	0.00401	2FG_2CK
GO:0003824	MF	catalytic activity	7941	1256	1167.48	3.10E-05	0FG_0CK
GO:0003824	MF	catalytic activity	7941	109	92.02	0.0064	2PG_2CK
GO:0003824	MF	catalytic activity	7941	1094	969.56	5.00E-10	2FG_2CK
GO:0003824	MF	catalytic activity	7941	915	824.07	8.70E-07	120d_4FG_120d_4CK
GO:0003824	MF	catalytic activity	7941	406	333.64	3.30E-09	120d_0PG_120d_0CK
GO:0003824	MF	catalytic activity	7941	1211	1018.91	1.00E-20	120d_0FG_120d_0CK
GO:0003824	MF	catalytic activity	7941	249	218.48	0.00153	120d_2PG_120d_2CK
GO:0003824	MF	catalytic activity	7941	935	787.57	9.50E-16	120d_2FG_120d_2CK
GO:0043169	MF	cation binding	1895	36	21.96	0.0018	2PG_2CK
GO:0043169	MF	cation binding	1895	248	187.94	1.20E-06	120d_2FG_120d_2CK
GO:0008324	MF	cation transmembrane transporter activit...	224	38	23.25	0.0016	120d_4FG_120d_4CK
GO:0019829	MF	cation-transporting ATPase activity	28	8	2.91	0.00615	120d_4FG_120d_4CK
GO:0051087	MF	chaperone binding	20	6	0.59	1.70E-05	0PG_0CK
GO:0008061	MF	chitin binding	12	2	0.14	0.0082	2PG_2CK
GO:0050662	MF	coenzyme binding	298	61	43.81	0.004	0FG_0CK
GO:0050662	MF	coenzyme binding	298	59	36.38	0.00011	2FG_2CK
GO:0050662	MF	coenzyme binding	298	47	30.92	0.00233	120d_4FG_120d_4CK
GO:0050662	MF	coenzyme binding	298	59	38.24	0.00041	120d_0FG_120d_0CK
GO:0050662	MF	coenzyme binding	298	50	29.55	0.00014	120d_2FG_120d_2CK
GO:0048037	MF	cofactor binding	419	88	61.6	0.00027	0FG_0CK
GO:0048037	MF	cofactor binding	419	85	51.16	1.30E-06	2FG_2CK
GO:0048037	MF	cofactor binding	419	76	43.48	8.10E-07	120d_4FG_120d_4CK
GO:0048037	MF	cofactor binding	419	85	53.76	9.40E-06	120d_0FG_120d_0CK
GO:0048037	MF	cofactor binding	419	21	11.53	0.00618	120d_2PG_120d_2CK
GO:0048037	MF	cofactor binding	419	73	41.56	1.20E-06	120d_2FG_120d_2CK
GO:0004129	MF	cytochrome-c oxidase activity	11	6	1.14	0.00036	120d_4FG_120d_4CK
GO:0051213	MF	dioxygenase activity	189	8	2.19	0.0016	2PG_2CK
GO:0051213	MF	dioxygenase activity	189	12	5.2	0.00617	120d_2PG_120d_2CK
GO:0019104	MF	DNA N-glycosylase activity	12	3	0.35	0.00452	0PG_0CK
GO:0009055	MF	electron carrier activity	522	15	6.05	0.0011	2PG_2CK
GO:0009055	MF	electron carrier activity	522	83	54.17	4.80E-05	120d_4FG_120d_4CK
GO:0009055	MF	electron carrier activity	522	27	14.36	0.0013	120d_2PG_120d_2CK
GO:0009055	MF	electron carrier activity	522	88	51.77	4.10E-07	120d_2FG_120d_2CK
GO:0004332	MF	fructose-bisphosphate aldolase activity	11	7	1.62	0.00028	0FG_0CK
GO:0004332	MF	fructose-bisphosphate aldolase activity	11	7	1.34	8.40E-05	2FG_2CK
GO:0004332	MF	fructose-bisphosphate aldolase activity	11	7	1.14	2.90E-05	120d_4FG_120d_4CK
GO:0004332	MF	fructose-bisphosphate aldolase activity	11	4	0.46	0.0008	120d_0PG_120d_0CK
GO:0004332	MF	fructose-bisphosphate aldolase activity	11	7	1.41	0.00012	120d_0FG_120d_0CK
GO:0004332	MF	fructose-bisphosphate aldolase activity	11	7	1.09	2.10E-05	120d_2FG_120d_2CK
GO:0004372	MF	glycine hydroxymethyltransferase activit...	10	5	1.47	0.00903	0FG_0CK
GO:0031072	MF	heat shock protein binding	128	28	15.63	0.00143	2FG_2CK
GO:0020037	MF	heme binding	470	14	5.45	0.0012	2PG_2CK

GO:0020037	MF	heme binding	470	38	19.75	8.90E-05	120d_0PG_120d_0CK
GO:0020037	MF	heme binding	470	30	12.93	1.80E-05	120d_2PG_120d_2CK
GO:0020037	MF	heme binding	470	71	46.61	0.0002	120d_2FG_120d_2CK
GO:0015002	MF	heme-copper terminal oxidase activity	11	6	1.14	0.00036	120d_4FG_120d_4CK
GO:0015078	MF	hydrogen ion transmembrane transporter a...	113	26	11.73	7.30E-05	120d_4FG_120d_4CK
GO:0016820	MF	hydrolase activity, acting on acid anhyd...	105	27	15.44	0.00214	0FG_0CK
GO:0016820	MF	hydrolase activity, acting on acid anhyd...	105	23	12.82	0.00357	2FG_2CK
GO:0016820	MF	hydrolase activity, acting on acid anhyd...	105	20	10.9	0.00527	120d_4FG_120d_4CK
GO:0016820	MF	hydrolase activity, acting on acid anhyd...	105	23	13.47	0.00658	120d_0FG_120d_0CK
GO:0016798	MF	hydrolase activity, acting on glycosyl b...	473	27	13.87	0.00078	0PG_0CK
GO:0004553	MF	hydrolase activity, hydrolyzing O-glycos...	448	24	13.14	0.00343	0PG_0CK
GO:0016836	MF	hydro-lyase activity	37	11	3.84	0.00097	120d_4FG_120d_4CK
GO:0016836	MF	hydro-lyase activity	37	7	1.55	0.00077	120d_0PG_120d_0CK
GO:0016836	MF	hydro-lyase activity	37	10	3.67	0.00255	120d_2FG_120d_2CK
GO:0042802	MF	identical protein binding	92	8	2.7	0.00558	0PG_0CK
GO:0022890	MF	inorganic cation transmembrane transport...	198	37	20.55	0.00028	120d_4FG_120d_4CK
GO:0016860	MF	intramolecular oxidoreductase activity	23	8	2.81	0.00438	2FG_2CK
GO:0016860	MF	intramolecular oxidoreductase activity	23	5	0.97	0.00231	120d_0PG_120d_0CK
GO:0016860	MF	intramolecular oxidoreductase activity	23	8	2.95	0.00596	120d_0FG_120d_0CK
GO:0016861	MF	intramolecular oxidoreductase activity, ...	20	5	0.84	0.00118	120d_0PG_120d_0CK
GO:0016861	MF	intramolecular oxidoreductase activity, ...	20	7	2.57	0.0096	120d_0FG_120d_0CK
GO:0015075	MF	ion transmembrane transporter activity	344	55	35.7	0.00075	120d_4FG_120d_4CK
GO:0005506	MF	iron ion binding	378	15	4.38	3.50E-05	2PG_2CK
GO:0005506	MF	iron ion binding	378	64	46.15	0.004	2FG_2CK
GO:0005506	MF	iron ion binding	378	66	39.23	1.50E-05	120d_4FG_120d_4CK
GO:0005506	MF	iron ion binding	378	32	15.88	0.00014	120d_0PG_120d_0CK
GO:0005506	MF	iron ion binding	378	68	48.5	0.00227	120d_0FG_120d_0CK
GO:0005506	MF	iron ion binding	378	27	10.4	6.30E-06	120d_2PG_120d_2CK
GO:0005506	MF	iron ion binding	378	78	37.49	2.30E-10	120d_2FG_120d_2CK
GO:0051536	MF	iron-sulfur cluster binding	80	21	11.76	0.00489	0FG_0CK
GO:0051536	MF	iron-sulfur cluster binding	80	20	9.77	0.00122	2FG_2CK
GO:0051536	MF	iron-sulfur cluster binding	80	21	8.3	4.70E-05	120d_4FG_120d_4CK
GO:0016853	MF	isomerase activity	146	40	21.46	4.80E-05	0FG_0CK
GO:0016853	MF	isomerase activity	146	31	17.83	0.00137	2FG_2CK
GO:0016853	MF	isomerase activity	146	15	6.13	0.00124	120d_0PG_120d_0CK
GO:0016853	MF	isomerase activity	146	32	18.73	0.00152	120d_0FG_120d_0CK
GO:0016874	MF	ligase activity	288	55	36.95	0.0015	120d_0FG_120d_0CK
GO:0016876	MF	ligase activity, forming aminoacyl-tRNA ...	61	18	8.97	0.00227	0FG_0CK
GO:0016876	MF	ligase activity, forming aminoacyl-tRNA ...	61	17	7.45	0.00075	2FG_2CK
GO:0016875	MF	ligase activity, forming carbon-oxygen b...	61	18	8.97	0.00227	0FG_0CK
GO:0016875	MF	ligase activity, forming carbon-oxygen b...	61	17	7.45	0.00075	2FG_2CK
GO:0016829	MF	lyase activity	223	52	32.79	0.00039	0FG_0CK
GO:0016829	MF	lyase activity	223	53	27.23	1.10E-06	2FG_2CK
GO:0016829	MF	lyase activity	223	42	23.14	9.50E-05	120d_4FG_120d_4CK

GO:0016829	MF	lyase activity	223	22	9.37	0.00018	120d_0PG_120d_0CK
GO:0016829	MF	lyase activity	223	46	28.61	0.00068	120d_0FG_120d_0CK
GO:0016829	MF	lyase activity	223	14	6.14	0.00357	120d_2PG_120d_2CK
GO:0016829	MF	lyase activity	223	43	22.12	1.50E-05	120d_2FG_120d_2CK
GO:0051540	MF	metal cluster binding	80	21	11.76	0.00489	0FG_0CK
GO:0051540	MF	metal cluster binding	80	20	9.77	0.00122	2FG_2CK
GO:0051540	MF	metal cluster binding	80	21	8.3	4.70E-05	120d_4FG_120d_4CK
GO:0046872	MF	metal ion binding	1884	36	21.83	0.0016	2PG_2CK
GO:0046872	MF	metal ion binding	1884	246	186.85	1.60E-06	120d_2FG_120d_2CK
GO:0015077	MF	monovalent inorganic cation transmembran...	161	32	16.71	0.00023	120d_4FG_120d_4CK
GO:0051287	MF	NAD binding	43	13	6.32	0.00709	0FG_0CK
GO:0051287	MF	NAD binding	43	15	5.25	0.0001	2FG_2CK
GO:0051287	MF	NAD binding	43	13	4.46	0.00028	120d_4FG_120d_4CK
GO:0051287	MF	NAD binding	43	13	4.26	0.00018	120d_2FG_120d_2CK
GO:0004576	MF	oligosaccharyl transferase activity	12	6	1.47	0.00158	2FG_2CK
GO:0043177	MF	organic acid binding	39	7	1.64	0.00106	120d_0PG_120d_0CK
GO:0043177	MF	organic acid binding	39	10	3.87	0.00388	120d_2FG_120d_2CK
GO:0016491	MF	oxidoreductase activity	1615	308	237.44	2.30E-07	0FG_0CK
GO:0016491	MF	oxidoreductase activity	1615	40	18.71	2.50E-06	2PG_2CK
GO:0016491	MF	oxidoreductase activity	1615	288	197.18	2.40E-12	2FG_2CK
GO:0016491	MF	oxidoreductase activity	1615	287	167.6	7.60E-22	120d_4FG_120d_4CK
GO:0016491	MF	oxidoreductase activity	1615	112	67.85	5.00E-08	120d_0PG_120d_0CK
GO:0016491	MF	oxidoreductase activity	1615	305	207.22	1.60E-13	120d_0FG_120d_0CK
GO:0016491	MF	oxidoreductase activity	1615	79	44.43	2.40E-07	120d_2PG_120d_2CK
GO:0016491	MF	oxidoreductase activity	1615	275	160.17	4.60E-21	120d_2FG_120d_2CK
GO:0016675	MF	oxidoreductase activity, acting on a hem...	11	6	1.14	0.00036	120d_4FG_120d_4CK
GO:0016676	MF	oxidoreductase activity, acting on a hem...	11	6	1.14	0.00036	120d_4FG_120d_4CK
GO:0016614	MF	oxidoreductase activity, acting on CH-OH...	155	40	22.79	0.0002	0FG_0CK
GO:0016614	MF	oxidoreductase activity, acting on CH-OH...	155	31	18.92	0.00364	2FG_2CK
GO:0016614	MF	oxidoreductase activity, acting on CH-OH...	155	37	16.09	9.40E-07	120d_4FG_120d_4CK
GO:0016614	MF	oxidoreductase activity, acting on CH-OH...	155	14	6.51	0.00576	120d_0PG_120d_0CK
GO:0016614	MF	oxidoreductase activity, acting on CH-OH...	155	37	19.89	0.00011	120d_0FG_120d_0CK
GO:0016614	MF	oxidoreductase activity, acting on CH-OH...	155	11	4.26	0.00376	120d_2PG_120d_2CK
GO:0016614	MF	oxidoreductase activity, acting on CH-OH...	155	37	15.37	3.10E-07	120d_2FG_120d_2CK
GO:0016679	MF	oxidoreductase activity, acting on diphe...	12	5	1.25	0.00507	120d_4FG_120d_4CK
GO:0016705	MF	oxidoreductase activity, acting on paire...	517	18	5.99	3.20E-05	2PG_2CK
GO:0016706	MF	oxidoreductase activity, acting on paire...	156	6	1.81	0.0097	2PG_2CK
GO:0016705	MF	oxidoreductase activity, acting on paire...	517	72	53.65	0.00576	120d_4FG_120d_4CK
GO:0016705	MF	oxidoreductase activity, acting on paire...	517	36	21.72	0.00211	120d_0PG_120d_0CK
GO:0016717	MF	oxidoreductase activity, acting on paire...	15	6	1.92	0.00789	120d_0FG_120d_0CK
GO:0016705	MF	oxidoreductase activity, acting on paire...	517	35	14.22	9.10E-07	120d_2PG_120d_2CK
GO:0016705	MF	oxidoreductase activity, acting on paire...	517	82	51.27	1.20E-05	120d_2FG_120d_2CK
GO:0016717	MF	oxidoreductase activity, acting on paire...	15	7	1.49	0.00029	120d_2FG_120d_2CK

GO:0016701	MF	oxidoreductase activity, acting on singl...	44	12	5.37	0.00527	2FG_2CK
GO:0016701	MF	oxidoreductase activity, acting on singl...	44	12	4.57	0.00134	120d_4FG_120d_4CK
GO:0016701	MF	oxidoreductase activity, acting on singl...	44	12	5.65	0.00783	120d_0FG_120d_0CK
GO:0016701	MF	oxidoreductase activity, acting on singl...	44	13	4.36	0.00023	120d_2FG_120d_2CK
GO:0016620	MF	oxidoreductase activity, acting on the a...	30	11	4.41	0.00248	0FG_0CK
GO:0016903	MF	oxidoreductase activity, acting on the a...	44	14	6.47	0.00316	0FG_0CK
GO:0016620	MF	oxidoreductase activity, acting on the a...	30	9	3.11	0.00258	120d_4FG_120d_4CK
GO:0016903	MF	oxidoreductase activity, acting on the a...	44	13	4.57	0.00037	120d_4FG_120d_4CK
GO:0016620	MF	oxidoreductase activity, acting on the a...	30	8	2.98	0.00735	120d_2FG_120d_2CK
GO:0016903	MF	oxidoreductase activity, acting on the a...	44	11	4.36	0.0031	120d_2FG_120d_2CK
GO:0016616	MF	oxidoreductase activity, acting on the C...	141	39	20.73	4.70E-05	0FG_0CK
GO:0016616	MF	oxidoreductase activity, acting on the C...	141	30	17.22	0.00157	2FG_2CK
GO:0016616	MF	oxidoreductase activity, acting on the C...	141	34	14.63	2.00E-06	120d_4FG_120d_4CK
GO:0016616	MF	oxidoreductase activity, acting on the C...	141	14	5.92	0.00246	120d_0PG_120d_0CK
GO:0016616	MF	oxidoreductase activity, acting on the C...	141	34	18.09	0.00017	120d_0FG_120d_0CK
GO:0016616	MF	oxidoreductase activity, acting on the C...	141	11	3.88	0.0018	120d_2PG_120d_2CK
GO:0016616	MF	oxidoreductase activity, acting on the C...	141	35	13.98	2.30E-07	120d_2FG_120d_2CK
GO:0016638	MF	oxidoreductase activity, acting on the C...	18	6	1.79	0.00613	120d_2FG_120d_2CK
GO:0008443	MF	phosphofructokinase activity	12	6	1.76	0.00417	0FG_0CK
GO:0008443	MF	phosphofructokinase activity	12	5	1.25	0.00507	120d_4FG_120d_4CK
GO:0030955	MF	potassium ion binding	17	6	1.76	0.00558	120d_4FG_120d_4CK
GO:0030955	MF	potassium ion binding	17	6	1.69	0.00445	120d_2FG_120d_2CK
GO:0015405	MF	P-P-bond-hydrolysis-driven transmembrane...	115	28	16.91	0.00418	0FG_0CK
GO:0015399	MF	primary active transmembrane transporter...	115	28	16.91	0.00418	0FG_0CK
GO:0046933	MF	proton-transporting ATP synthase activit...	22	8	2.28	0.00111	120d_4FG_120d_4CK
GO:0030170	MF	pyridoxal phosphate binding	93	23	13.67	0.00729	0FG_0CK
GO:0030170	MF	pyridoxal phosphate binding	93	20	11.35	0.0078	2FG_2CK
GO:0030170	MF	pyridoxal phosphate binding	93	20	9.65	0.00118	120d_4FG_120d_4CK
GO:0030170	MF	pyridoxal phosphate binding	93	21	11.93	0.00639	120d_0FG_120d_0CK
GO:0030170	MF	pyridoxal phosphate binding	93	17	9.22	0.00941	120d_2FG_120d_2CK
GO:0004743	MF	pyruvate kinase activity	17	6	1.76	0.00558	120d_4FG_120d_4CK
GO:0004743	MF	pyruvate kinase activity	17	6	1.69	0.00445	120d_2FG_120d_2CK
GO:0019843	MF	rRNA binding	14	7	2.06	0.00195	0FG_0CK
GO:0004252	MF	serine-type endopeptidase activity	126	10	3.7	0.00397	0PG_0CK
GO:0003735	MF	structural constituent of ribosome	342	140	50.28	< 1e-30	0FG_0CK
GO:0003735	MF	structural constituent of ribosome	342	111	41.76	2.20E-23	2FG_2CK
GO:0003735	MF	structural constituent of ribosome	342	131	35.49	< 1e-30	120d_4FG_120d_4CK
GO:0005198	MF	structural molecule activity	392	156	57.63	< 1e-30	0FG_0CK
GO:0005198	MF	structural molecule activity	392	122	47.86	8.40E-24	2FG_2CK
GO:0005198	MF	structural molecule activity	392	137	40.68	< 1e-30	120d_4FG_120d_4CK
GO:0022891	MF	substrate-specific transmembrane transpo...	362	57	37.57	0.00089	120d_4FG_120d_4CK
GO:0046906	MF	tetrapyrrole binding	473	14	5.48	0.0012	2PG_2CK
GO:0046906	MF	tetrapyrrole binding	473	38	19.87	0.0001	120d_0PG_120d_0CK

GO:0046906	MF	tetrapyrrole binding	473	30	13.01	2.00E-05	120d_2PG_120d_2CK
GO:0046906	MF	tetrapyrrole binding	473	71	46.91	0.00025	120d_2FG_120d_2CK
GO:0004298	MF	threonine-type endopeptidase activity	29	16	3.72	6.60E-08	120d_0FG_120d_0CK
GO:0004298	MF	threonine-type endopeptidase activity	29	8	2.88	0.00589	120d_2FG_120d_2CK
GO:0070003	MF	threonine-type peptidase activity	29	16	3.72	6.60E-08	120d_0FG_120d_0CK
GO:0070003	MF	threonine-type peptidase activity	29	8	2.88	0.00589	120d_2FG_120d_2CK
GO:0046912	MF	transferase activity, transferring acyl ...	11	6	1.34	0.00088	2FG_2CK
GO:0046912	MF	transferase activity, transferring acyl ...	11	6	1.14	0.00036	120d_4FG_120d_4CK
GO:0016746	MF	transferase activity, transferring acyl ...	272	49	34.9	0.00838	120d_0FG_120d_0CK
GO:0016758	MF	transferase activity, transferring hexos...	345	58	42.12	0.00689	2FG_2CK
GO:0016769	MF	transferase activity, transferring nitro...	47	12	5.74	0.00924	2FG_2CK
GO:0016769	MF	transferase activity, transferring nitro...	47	11	4.88	0.00749	120d_4FG_120d_4CK
GO:0046914	MF	transition metal ion binding	1509	28	17.49	0.0084	2PG_2CK
GO:0046914	MF	transition metal ion binding	1509	182	149.66	0.00239	120d_2FG_120d_2CK
GO:0022857	MF	transmembrane transporter activity	586	87	60.81	0.00036	120d_4FG_120d_4CK
GO:0005215	MF	transporter activity	811	122	84.16	1.30E-05	120d_4FG_120d_4CK
GO:0005215	MF	transporter activity	811	53	34.07	0.00092	120d_0PG_120d_0CK
GO:0005215	MF	transporter activity	811	38	22.31	0.00096	120d_2PG_120d_2CK

Note: “CK”indicates control, “PG”indicates partial girdled, and “FG” indicates fully girdled. “0”indicates 0hr, “2” indicates 2hr and “4”indicates 4hr. “120d”indicates 120-day old leaf, others not labeld with days came from 45-day old leaf.

Supplemental Table S4. Genes annotated to photosynthesis, N-metabolism and tetrapyrrole synthesis pathways. The extent of differential expression is measured in terms of log2 fold change of treatment vs. control.

Table Class	Cassava ID	Mapman Category	Short Description	PG0	FG0	PG2	FG2	PG4	FG4	PG0	FG0	PG2	FG2
				120 day						45 day			
Light Reaction	cassava4.1_028060m.g	PS.lightreaction.ATP synthase	ATP synthase subunit alpha	-1.78	-1.02	-0.94	-0.45	-0.15	-2.65	0.55	-2.27	-0.45	-1.77
	cassava4.1_014114m.g	PS.lightreaction.photosystem I.LHC-I	photosystem I light harvesting complex gene 6	-1.41	-2.41	-0.81	-1.83	-0.09	-1.89	-0.91	-2.00	-0.72	-1.93
	cassava4.1_013714m.g	PS.lightreaction.photosystem II.LHC-II	light harvesting complex photosystem II	-1.33	-3.17	-0.60	-3.56	-0.13	-4.56	-0.92	-3.18	-1.20	-3.90
	cassava4.1_010721m.g	PS.lightreaction.cyclic electron flow-chlororespiration	NDH-dependent cyclic electron flow 1	-1.29	-4.78	-0.91	-4.45	0.05	-3.55	-0.68	-2.43	-0.48	-2.17
	cassava4.1_017367m.g	PS.lightreaction.photosystem II.PSII polypeptide subunits	photosystem II reaction center PSB28 protein	-1.29	-2.65	-0.95	-2.18	-0.36	-1.30	-0.75	-1.84	-0.68	-1.77
	cassava4.1_015252m.g	PS.lightreaction.photosystem II.PSII polypeptide subunits	PsbP-like protein 2	-1.18	-2.58	-0.83	-2.64	-0.07	-2.42	-0.56	-1.56	-0.54	-1.78
	cassava4.1_013294m.g	PS.lightreaction.photosystem II.LHC-II	light harvesting complex photosystem II	-1.04	-3.86	0.06	-3.04	0.09	-2.67	-0.72	-2.45	-0.42	-2.60
	cassava4.1_015152m.g	PS.lightreaction.photosystem II.PSII polypeptide subunits	PsbP-like protein 1	-1.03	-2.97	-0.53	-2.28	-0.39	-2.95	-0.51	-2.61	-0.63	-2.41
	cassava4.1_010832m.g	PS.lightreaction.other electron carrier (ox/red).ferredoxin reductase	ferredoxin-NADP(+)-oxidoreductase 2	-1.03	-3.21	-0.65	-2.64	-0.17	-3.00	-0.90	-2.69	-1.14	-3.44
	cassava4.1_017708m.g	PS.lightreaction.photosystem I.PSI polypeptide subunits	photosystem I P subunit	-1.02	-4.00	-0.37	-3.02	-0.09	-4.06	-0.65	-3.38	-1.15	-3.80
	cassava4.1_012195m.g	PS.lightreaction.photosystem I.LHC-I	light-harvesting chlorophyll-protein complex I subunit A4	-0.99	-3.74	-0.06	-2.20	0.14	-1.97	-0.52	-1.79	-0.33	-1.93
	cassava4.1_012460m.g	PS.lightreaction.photosystem II.LHC-II	light-harvesting chlorophyll-protein complex II subunit B1	-0.97	-5.16	0.09	-2.62	0.07	-2.03	-0.93	-1.94	-0.32	-1.94
	cassava4.1_019328m.g	PS.lightreaction.photosystem II.PSII polypeptide subunits	photosystem II subunit X	-0.97	-3.65	-0.72	-3.15	0.03	-2.36	-0.27	-2.53	-0.72	-2.38
	cassava4.1_011470m.g	PS.lightreaction.photosystem II.PSII polypeptide subunits	photosystem II subunit O-2	-0.88	-3.07	-0.29	-2.54	0.00	-2.59	-0.66	-2.56	-0.75	-2.74
	cassava4.1_011539m.g	PS.lightreaction.photosystem II.PSII polypeptide	photosystem II subunit O-2	-0.88	-2.69	-0.27	-2.54	-0.06	-2.76	-0.64	-2.32	-0.91	-2.99

		subunits											
	cassava4.1_014055m.g	PS.lightreaction.photosystem II.LHC-II	photosystem II light harvesting complex gene 2.1	-0.86	-3.85	-0.32	-2.62	-0.08	-2.74	-0.76	-2.36	-0.84	-2.42
	cassava4.1_009836m.g	PS.lightreaction.ATP synthase.gamma chain	ATPase, F1 complex, gamma subunit protein	-0.85	-2.26	-0.75	-2.61	-0.24	-2.50	-0.66	-2.37	-1.08	-2.93
	cassava4.1_017705m.g	PS.lightreaction.other electron carrier (ox/red).plastocyanin	Cupredoxin superfamily protein	-0.85	-3.18	-0.50	-2.90	-0.09	-2.35	-0.81	-2.15	-1.10	-2.72
	cassava4.1_018500m.g	PS.lightreaction.other electron carrier (ox/red).ferredoxin	2Fe-2S ferredoxin-like superfamily protein	-0.84	-4.02	-0.99	-3.76	0.06	-2.71	-0.39	-3.14	-1.00	-3.32
	cassava4.1_013980m.g	PS.lightreaction.photosystem I.LHC-I	photosystem I light harvesting complex gene 5	-0.84	-2.47	-0.39	-2.20	0.11	-1.98	-0.38	-1.04	-0.25	-0.79
	cassava4.1_015521m.g	PS.lightreaction.photosystem II.PSII polypeptide subunits	photosystem II subunit Q-2	-0.84	-2.62	-0.16	-2.82	-0.01	-3.03	-0.64	-2.29	-0.77	-2.74
	cassava4.1_017374m.g	PS.lightreaction.photosystem I.PSI polypeptide subunits	photosystem I P subunit	-0.83	-2.49	-0.24	-2.92	-0.01	-3.17	-0.40	-2.42	-0.72	-2.90
	cassava4.1_030348m.g	PS.lightreaction.photosystem I.PSI polypeptide subunits	photosystem I subunit l	-0.83	-2.98	-0.11	-2.59	0.00	-2.57	-0.66	-2.33	-0.90	-2.72
	cassava4.1_014559m.g	PS.lightreaction.photosystem I.LHC-I	light-harvesting chlorophyll-protein complex I subunit A4	-0.81	-3.94	-0.03	-2.65	0.04	-2.03	-0.20	-1.54	-0.28	-2.01
	cassava4.1_018914m.g	PS.lightreaction.photosystem II.PSII polypeptide subunits	photosystem II reaction center W	-0.81	-2.88	-0.93	-2.71	0.01	-2.24	-0.34	-1.80	-0.66	-2.34
	cassava4.1_014926m.g	PS.lightreaction.photosystem I.LHC-I	photosystem I light harvesting complex gene 1	-0.81	-3.93	0.03	-2.55	-0.01	-1.91	-0.53	-1.94	-0.10	-1.74
	cassava4.1_017909m.g	PS.lightreaction.NADH DH	NAD(P)H:plastoquinone dehydrogenase complex subunit O	-0.80	-1.58	-0.39	-0.74	0.02	-0.72	-0.49	-0.92	-0.39	-0.57
	cassava4.1_019060m.g	PS.lightreaction.photosystem I.PSI polypeptide subunits	photosystem I subunit K	-0.79	-3.38	-0.48	-3.14	0.02	-3.22	-0.60	-2.42	-1.17	-3.22
	cassava4.1_010341m.g	PS.lightreaction.other electron carrier (ox/red).ferredoxin reductase	ferredoxin-NADP(+)-oxidoreductase 2	-0.78	-3.02	-0.77	-3.76	-0.13	-3.37	-0.45	-2.29	-1.05	-3.53
	cassava4.1_017061m.g	PS.lightreaction.NADH DH	inorganic carbon transport protein-related	-0.77	-2.42	-0.73	-2.59	-0.24	-3.31	-0.57	-1.62	-0.92	-2.36
	cassava4.1_010324m.g	PS.lightreaction.other electron carrier (ox/red).ferredoxin reductase	ferredoxin-NADP(+)-oxidoreductase 1	-0.77	-2.08	-0.39	-2.19	0.03	-2.08	-0.50	-1.85	-0.81	-2.51
	cassava4.1_017065m.g	PS.lightreaction.photosystem II.PSII polypeptide subunits	PsbQ-like 2	-0.76	-2.18	-0.80	-3.17	0.09	-2.78	-0.42	-1.44	-0.76	-2.22
	cassava4.1_014170m.g	PS.lightreaction.photosystem II.PSII polypeptide subunits	Mog1/PsbP/DUF1795-like photosystem II reaction center PsbP family protein	-0.75	-1.19	-0.24	-0.96	0.01	-1.61	-0.33	-0.96	-0.07	-0.84

	cassava4.1_017073m.g	PS.lightreaction.photosystem I.LHC-I	one-helix protein 2	-0.75	-1.47	-0.15	-1.45	-0.07	-1.04	-0.32	-1.70	-0.92	-2.52
	cassava4.1_012896m.g	PS.lightreaction.photosystem II.PSII polypeptide subunits	Mog1/PsbP/DUF1795-like photosystem II reaction center PsbP family protein	-0.75	-1.60	-0.35	-0.67	-0.27	-0.66	-0.80	-0.49	0.08	0.28
	cassava4.1_015488m.g	PS.lightreaction.cytochrome b6/f.iron sulfur subunit	photosynthetic electron transfer C	-0.74	-2.03	-0.42	-1.72	-0.05	-1.83	-0.77	-1.98	-0.88	-2.24
	cassava4.1_019108m.g	PS.lightreaction.photosystem I.PSI polypeptide subunits	photosystem I subunit K	-0.73	-3.55	-0.17	-3.16	0.04	-3.16	-0.60	-2.27	-1.22	-3.25
	cassava4.1_015604m.g	PS.lightreaction.photosystem II.PSII polypeptide subunits	PsbQ-like 1	-0.73	-1.80	-0.87	-1.94	0.09	-1.48	-0.56	0.56	-0.09	0.63
	cassava4.1_014868m.g	PS.lightreaction.ATP synthase.delta chain	ATP synthase delta-subunit gene	-0.72	-2.38	-0.55	-1.95	-0.08	-1.92	-0.73	-2.01	-0.95	-2.54
	cassava4.1_015015m.g	PS.lightreaction.photosystem II.PSII polypeptide subunits	PsbP-like protein 1	-0.71	-1.85	-0.63	-1.60	0.11	-1.79	-0.38	-1.27	-0.53	-1.33
	cassava4.1_014216m.g	PS.lightreaction.photosystem II.PSII polypeptide subunits	photosystem II subunit P-1	-0.70	-2.03	-0.08	-2.04	0.01	-2.75	-0.73	-2.05	-0.78	-2.51
	cassava4.1_014117m.g	PS.lightreaction.photosystem II.LHC-II	light-harvesting chlorophyll B-binding protein 3	-0.69	-4.02	-0.11	-2.35	0.06	-2.03	-0.74	-1.83	-0.47	-2.33
	cassava4.1_015490m.g	PS.lightreaction.photosystem I.PSI polypeptide subunits	photosystem I subunit F	-0.68	-2.28	-0.21	-2.40	0.07	-2.45	-0.67	-2.13	-0.86	-2.55
	cassava4.1_008115m.g	PS.lightreaction.NADH DH	unknow	-0.68	-0.63	-0.13	-0.57	-0.09	-1.03	-0.04	0.10	-0.02	-0.23
	cassava4.1_014906m.g	PS.lightreaction.photosystem I.LHC-I	photosystem I light harvesting complex gene 1	-0.68	-3.19	-0.13	-2.28	-0.08	-1.34	-0.42	-1.60	-0.53	-2.08
	cassava4.1_014115m.g	PS.lightreaction.ATP synthase.subunit B_ (ATPX)	ATPase, F0 complex, subunit B/B', bacterial/chloroplast	-0.67	-2.46	-0.49	-1.64	-0.04	-1.74	-0.58	-1.81	-0.88	-1.67
	cassava4.1_018335m.g	PS.lightreaction.photosystem I.PSI polypeptide subunits	photosystem I subunit O	-0.63	-2.45	-0.11	-2.70	0.03	-3.21	-0.70	-2.46	-0.81	-2.88
	cassava4.1_014010m.g	PS.lightreaction.photosystem II.PSII polypeptide subunits	photosystem II subunit P-1	-0.62	-1.94	-0.07	-1.68	-0.08	-1.22	-0.80	-1.01	-0.51	-1.31
	cassava4.1_017616m.g	PS.lightreaction.photosystem I.PSI polypeptide subunits	photosystem I reaction center subunit PSI-N, chloroplast, putative / PSI-N, putative (PSAN)	-0.61	-3.27	-0.69	-3.38	0.08	-2.49	-0.34	-2.34	-1.08	-3.04
	cassava4.1_015083m.g	PS.lightreaction.photosystem II.PSII polypeptide subunits	unclear	-0.61	-2.30	-0.63	-2.14	-0.13	-1.82	-0.49	-2.37	-0.80	-2.14
	cassava4.1_011842m.g	PS.lightreaction.cyclic electron flow-chlororespiration	PGR5-LIKE A	-0.60	-1.80	-0.25	-2.07	-0.06	-1.97	-0.18	-1.80	-0.88	-2.53
	cassava4.1_013916m.g	PS.lightreaction.photosystem I.LHC-I	photosystem I light harvesting complex gene 2	-0.60	-3.24	0.15	-3.21	0.01	-2.78	-0.64	-2.45	-0.71	-2.91
	cassava4.1_020972m.g	PS.lightreaction.ATP synthase.subunit B_ (ATPX)	Ubiquitin-like superfamily protein	-0.59	-1.98	-0.52	-1.77	0.00	-1.40	-0.48	-1.57	-0.74	-1.83
	cassava4.1_016312m.g	PS.lightreaction.photosystem I.PSI polypeptide subunits	photosystem I subunit D-1	-0.58	-2.28	-0.16	-2.10	0.04	-1.56	-0.88	-1.54	-0.68	-2.07

	cassava4.1_013735m.g	PS.lightreaction.photosystem II.PSII polypeptide subunits	Chlorophyll A-B binding family protein	-0.57	-2.92	-0.25	-3.28	0.01	-3.37	-0.21	-1.70	-0.86	-2.97
	cassava4.1_015240m.g	PS.lightreaction.NADH DH	subunit NDH-M of NAD(P)H:plastoquinone dehydrogenase complex	-0.57	-1.48	-0.55	-1.85	-0.10	-1.82	-0.63	-0.94	-0.97	-2.14
	cassava4.1_018584m.g	PS.lightreaction.photosystem I.PSI polypeptide subunits	photosystem I subunit H-1	-0.54	-2.43	-0.24	-2.26	0.12	-2.59	-0.66	-1.74	-1.12	-2.61
	cassava4.1_016411m.g	PS.lightreaction.photosystem I.PSI polypeptide subunits	photosystem I subunit D-1	-0.54	-1.51	-0.25	-1.87	0.05	-1.92	-0.76	-1.59	-0.93	-2.29
	cassava4.1_013872m.g	PS.lightreaction.photosystem II.PSII polypeptide subunits	Mog1/PsbP/DUF1795-like photosystem II reaction center PsbP family protein	-0.52	-3.34	-0.07	-1.53	0.15	-1.39	0.44	-1.44	-0.45	-1.58
	cassava4.1_018544m.g	PS.lightreaction.photosystem I.PSI polypeptide subunits	photosystem I subunit E-2	-0.52	-2.16	-0.16	-2.33	0.06	-2.15	-0.63	-1.90	-0.73	-2.55
	cassava4.1_019040m.g	PS.lightreaction.photosystem II.PSII polypeptide subunits	photosystem II reaction center W	-0.52	-2.25	-0.42	-2.71	0.07	-2.40	-0.48	-2.00	-0.92	-2.53
	cassava4.1_010951m.g	PS.lightreaction.photosystem II.LHC-II	Chlorophyll A-B binding family protein	-0.50	-1.73	-0.01	-1.86	0.07	-1.87	-0.47	-1.82	-0.81	-2.53
	cassava4.1_026492m.g	PS.lightreaction.photosystem II.PSII polypeptide subunits	Photosystem II reaction center PsbP family protein	-0.49	-1.11	-0.09	-0.60	-0.16	-0.88	-0.41	-1.13	-0.72	-1.19
	cassava4.1_016712m.g	PS.lightreaction.photosystem II.PSII polypeptide subunits	photosystem II BY	-0.48	-1.69	-0.24	-1.96	-0.04	-1.67	-0.53	-1.36	-1.15	-2.34
	cassava4.1_018917m.g	PS.lightreaction.photosystem II.PSII polypeptide subunits	photosystem II reaction center W	-0.48	-2.08	-0.32	-2.14	0.01	-1.72	-0.34	-1.56	-0.84	-2.20
	cassava4.1_015547m.g	PS.lightreaction.NADH DH	oxidoreductases, acting on NADH or NADPH, quinone or similar compound as acceptor	-0.44	-1.90	-0.68	-1.77	0.05	-2.06	-0.75	-0.96	-0.85	-1.86
	cassava4.1_030615m.g	PS.lightreaction.ATP synthase.chloroplastic subunit a	ATPase, F0 complex, subunit A protein	-0.43	-0.49	-0.11	-0.09	0.46	-0.35	0.10	-2.20	-0.32	-1.59
	cassava4.1_016692m.g	PS.lightreaction.photosystem II.PSII polypeptide subunits	low psii accumulation2	-0.42	-0.67	-0.05	-0.37	0.23	-0.72	-0.85	-0.64	-0.66	-1.03
	cassava4.1_018334m.g	PS.lightreaction.photosystem I.PSI polypeptide subunits	photosystem I subunit G	-0.38	-1.68	-0.23	-2.02	0.00	-1.61	-0.39	-1.59	-0.70	-2.01
	cassava4.1_013957m.g	PS.lightreaction.photosystem I.LHC-I	photosystem I light harvesting complex gene 2	-0.37	-2.64	-0.14	-2.68	0.08	-2.53	-0.63	-1.72	-0.89	-2.68
	cassava4.1_016854m.g	PS.lightreaction.photosystem II.PSII polypeptide subunits	photosystem II BY	-0.37	-1.81	-0.13	-2.11	0.03	-1.83	-0.46	-1.36	-1.34	-2.59
	cassava4.1_018756m.g	PS.lightreaction.photosystem II.PSII polypeptide	photosystem II subunit R	-0.36	-1.80	-0.24	-1.92	-0.05	-2.05	-0.48	-1.42	-1.22	-2.90

		subunits											
	cassava4.1_013684m.g	PS.lightreaction.photosystem I.LHC-I	photosystem I light harvesting complex gene 3	-0.36	-2.84	-0.32	-2.82	0.04	-2.15	-0.41	-2.00	-0.78	-2.57
	cassava4.1_018388m.g	PS.lightreaction.other electron carrier (ox/red).ferredoxin	2Fe-2S ferredoxin-like superfamily protein	-0.35	-1.09	-0.71	-1.20	-0.34	-0.84	-0.32	-1.53	-0.51	-1.32
	cassava4.1_019355m.g	PS.lightreaction.cyclic electron flow-chlororespiration	proton gradient regulation 5	-0.34	-1.94	0.13	-0.03	-0.02	-0.32	-0.26	-0.05	-0.83	-1.61
	cassava4.1_004476m.g	PS.lightreaction.state transition	STT7 homolog STN7	-0.32	-1.32	-0.08	-1.20	0.04	-1.24	0.12	-1.84	-0.68	-2.04
	cassava4.1_019321m.g	PS.lightreaction.cyclic electron flow-chlororespiration	proton gradient regulation 5	-0.29	-2.37	-0.10	-1.31	0.03	-1.78	-0.17	-0.50	-1.02	-2.22
	cassava4.1_017598m.g	PS.lightreaction.photosystem II.PSII polypeptide subunits	photosystem II family protein	-0.28	-1.62	-0.17	-1.23	0.11	-1.36	-0.85	-1.41	-0.71	-1.66
	cassava4.1_004477m.g	PS.lightreaction.state transition	STT7 homolog STN7	-0.25	-1.61	-0.25	-2.00	0.00	-2.42	-0.17	-1.94	-0.71	-2.63
	cassava4.1_012618m.g	PS.lightreaction.other electron carrier (ox/red).ferredoxin oxireductase	FAD/NAD(P)-binding oxidoreductase	-0.24	-0.96	-0.19	-0.66	0.01	-0.32	-0.35	-1.17	-0.56	-1.53
	cassava4.1_013632m.g	PS.lightreaction.photosystem II.LHC-II	light harvesting complex photosystem II subunit 6	-0.21	-1.85	-0.22	-1.95	0.10	-1.71	-0.70	-1.00	-0.90	-2.00
	cassava4.1_031236m.g	PS.lightreaction.photosystem II.PSII polypeptide subunits	photosystem II reaction center protein A	-0.15	-0.97	-1.38	-1.02	0.36	-0.11	-0.45	-1.01	-0.94	-1.63
	cassava4.1_016776m.g	PS.lightreaction.other electron carrier (ox/red).ferredoxin	NDH-dependent cyclic electron flow 1	-0.09	-0.33	-0.60	-1.79	-0.25	-2.48	-0.60	-0.52	-0.91	-0.86
	cassava4.1_027296m.g	PS.lightreaction.photosystem I.PSI polypeptide subunits	Photosystem I, PsaA/PsaB protein	-0.04	-0.42	-1.97	-0.82	-0.14	-1.31	-0.53	-2.52	-0.10	-1.97
	cassava4.1_013335m.g	PS.lightreaction.cyclic electron flow-chlororespiration	post-illumination chlorophyll fluorescence increase	-0.03	-1.60	-0.03	-2.39	0.11	-2.90	-0.16	-1.29	-0.71	-2.74
	cassava4.1_018830m.g	PS.lightreaction.photosystem II.PSII polypeptide subunits	photosystem II subunit R	0.28	-0.12	-0.06	-1.59	0.27	-1.41	-0.24	-0.38	-0.92	-2.73
	cassava4.1_025967m.g	PS.lightreaction.ATP synthase	ATP synthase protein I -related	0.31	0.18	0.17	-1.60	0.03	-2.22	0.00	-0.64	-0.48	-1.71
	cassava4.1_029388m.g	PS.lightreaction.photosystem I.PSI polypeptide subunits	Photosystem I, PsaA/PsaB protein	0.34	-0.01	-1.34	-0.31	-0.55	-1.28	-0.46	-2.15	-0.11	-2.27
	cassava4.1_020912m.g	PS.lightreaction.ATP synthase.chloroplastic subunit a	ATPase, F0 complex, subunit A protein	0.35	0.29	-0.91	-0.66	0.91	-0.48	-0.29	-2.27	-0.43	-1.84
	cassava4.1_022954m.g	PS.lightreaction.photosystem I.PSI polypeptide subunits	Photosystem I, PsaA/PsaB protein	0.48	-1.13	-0.05	-0.66	-0.16	-1.20	-0.10	-1.78	0.05	-2.08
	cassava4.1_031110m.g	PS.lightreaction.photosystem II.PSII polypeptide subunits	photosystem II reaction center protein D	0.49	0.38	-0.65	-0.40	-1.05	-0.77	0.62	-2.04	-0.47	-2.67
Calvin Cycle	cassava4.1_029431m.g	PS.calvin cycle.Rib5P Isomerase	NagB/RpiA/CoA transferase-like superfamily protein	-1.53	-2.32	-0.73	-2.55	-0.03	-3.17	-1.05	-2.51	-0.93	-2.97

	cassava4.1_017243m.g	PS.calvin cycle.rubisco small subunit	Ribulose bisphosphate carboxylase (small chain) family protein	-1.48	-2.83	-0.71	-2.14	-0.13	-3.06	-1.31	-2.70	-0.82	-2.71
	cassava4.1_030397m.g	PS.calvin cycle.FBPase	high cyclic electron flow 1	-1.46	-3.28	0.45	-0.61	0.36	-2.44	-1.10	-3.65	-0.94	-2.08
	cassava4.1_012057m.g	PS.calvin cycle.TPI	triosephosphate isomerase	-1.39	-1.73	-0.39	-0.79	-0.29	-0.58	-1.15	-1.48	-0.74	-0.61
	cassava4.1_011197m.g	PS.calvin cycle.FBPase	Inositol monophosphatase family protein	-1.36	-3.76	-0.89	-3.04	-0.37	-3.56	-0.19	-3.54	-0.76	-3.53
	cassava4.1_007468m.g	PS.calvin cycle.GAP	glyceraldehyde-3-phosphate dehydrogenase B subunit	-1.24	-5.51	-0.65	-5.32	-0.11	-5.90	-0.49	-4.58	-0.91	-5.38
	cassava4.1_010561m.g	PS.calvin cycle.aldolase	Aldolase superfamily protein	-1.17	-4.18	-0.72	-4.45	-0.10	-4.50	-0.84	-3.41	-1.18	-4.14
	cassava4.1_006492m.g	PS.calvin cycle.rubisco interacting	Rubisco methyltransferase family protein	-1.15	-2.17	-0.72	-0.95	-0.07	-1.77	-0.88	-0.73	0.09	-0.49
	cassava4.1_006605m.g	PS.calvin cycle.phosphoglycerate kinase	Phosphoglycerate kinase family protein	-1.07	-3.08	-0.44	-2.95	-0.34	-3.22	-0.49	-2.47	-0.96	-3.34
	cassava4.1_008668m.g	PS.calvin cycle.FBPase	high cyclic electron flow 1	-1.07	-3.35	-0.55	-3.05	-0.02	-3.81	-0.82	-2.46	-0.91	-3.01
	cassava4.1_008849m.g	PS.calvin cycle.PRK	phosphoribulokinase	-1.04	-3.22	-0.58	-2.94	-0.11	-3.41	-0.52	-2.56	-0.97	-3.10
	cassava4.1_014454m.g	PS.calvin cycle.TPI	triosephosphate isomerase	-1.01	-2.70	-0.35	-1.79	-0.28	-1.66	-0.55	-2.79	-0.88	-2.74
	cassava4.1_008960m.g	PS.calvin cycle.GAP	glyceraldehyde 3-phosphate dehydrogenase A subunit	-0.88	-2.75	-0.56	-2.14	-0.09	-2.04	-0.77	-2.27	-0.91	-2.55
	cassava4.1_017170m.g	PS.calvin cycle.rubisco small subunit	Ribulose bisphosphate carboxylase (small chain) family protein	-0.88	-3.08	-0.49	-2.78	-0.09	-3.52	-0.52	-2.63	-0.82	-2.77
	cassava4.1_014243m.g	PS.calvin cycle.FBPase	Inositol monophosphatase family protein	-0.87	-3.71	-0.43	-2.52	-0.20	-3.82	-0.19	-2.93	-0.50	-3.12
	cassava4.1_012016m.g	PS.calvin cycle.TPI	triosephosphate isomerase	-0.83	-1.29	-0.23	-0.79	-0.15	-1.06	-0.77	-1.58	-0.87	-1.50
	cassava4.1_006803m.g	PS.calvin cycle.rubisco interacting	rubisco activase	-0.81	-3.62	-0.69	-3.00	-0.31	-3.54	-0.45	-2.90	-0.84	-3.52
	cassava4.1_002307m.g	PS.calvin cycle.transketolase	Transketolase	-0.80	-2.18	-0.27	-1.06	-0.06	-0.56	-0.38	-1.47	-0.68	-1.60
	cassava4.1_008965m.g	PS.calvin cycle.GAP	glyceraldehyde 3-phosphate dehydrogenase A subunit	-0.78	-2.98	-0.47	-2.20	-0.15	-2.36	-0.67	-2.44	-0.82	-2.60
	cassava4.1_013583m.g	PS	acclimation of photosynthesis to environment	-0.74	-2.09	-0.29	-1.71	0.11	-1.72	-0.42	-1.71	-0.93	-2.30
	cassava4.1_009395m.g	PS.calvin cycle.seduheptulose bisphosphatase	sedoheptulose-bisphosphatase	-0.73	-2.57	-0.52	-1.88	0.03	-2.07	-0.37	-1.59	-0.77	-2.15
	cassava4.1_008894m.g	PS.calvin cycle.PRK	phosphoribulokinase	-0.71	-1.81	-0.36	-1.65	-0.02	-2.00	-0.64	-1.41	-0.94	-2.35
	cassava4.1_007476m.g	PS.calvin cycle.GAP	glyceraldehyde-3-phosphate dehydrogenase B subunit	-0.68	-1.58	-0.33	-1.23	-0.10	-1.57	-0.45	-1.95	-0.55	-1.93
	cassava4.1_006183m.g	PS.calvin cycle.GAP	aldehyde dehydrogenase 11A3	-0.63	-2.11	-0.19	-0.53	-0.03	0.13	-0.10	-0.48	-0.71	-0.82
	cassava4.1_009163m.g	PS.calvin cycle.aldolase	fructose-bisphosphate aldolase 2	-0.57	-2.44	-0.23	-2.35	-0.01	-3.18	0.07	-2.81	-0.25	-2.84
	cassava4.1_009140m.g	PS.calvin cycle.aldolase	fructose-bisphosphate aldolase 2	-0.55	-2.12	-0.62	-2.29	0.09	-2.07	-0.47	-2.20	-0.50	-2.67
	cassava4.1_006596m.g	PS.calvin cycle.phosphoglycerate kinase	Phosphoglycerate kinase family protein	-0.51	-1.28	-0.27	-0.93	0.05	-0.85	-0.50	-1.40	-0.76	-2.00

	cassava4.1_009143m.g	PS.calvin cycle.aldolase	fructose-bisphosphate aldolase 2	-0.51	-2.79	-0.47	-2.20	-0.03	-1.77	-0.18	-1.58	-0.62	-2.17
	cassava4.1_017330m.g	PS.calvin cycle.rubisco large subunit	ribulose-bisphosphate carboxylases	-0.47	-0.25	-1.21	-0.37	0.11	-0.06	-1.09	-1.99	-0.83	-2.31
	cassava4.1_013479m.g	PS.calvin cycle.RPE	D-ribulose-5-phosphate-3-epimerase	-0.45	-1.70	-0.32	-1.86	-0.08	-2.41	-0.31	-1.90	-0.97	-2.82
	cassava4.1_006796m.g	PS.calvin cycle.rubisco interacting	rubisco activase	-0.31	-2.06	-0.27	-2.60	-0.12	-2.21	1.45	-1.09	-0.29	-2.37
	cassava4.1_033564m.g	PS.calvin cycle.rubisco interacting	P-loop containing nucleoside triphosphate hydrolases superfamily protein	-0.29	-1.46	-0.06	-0.88	0.03	-1.90	0.39	-2.44	-0.52	-2.20
	cassava4.1_009003m.g	PS.calvin cycle.phosphoglycerate kinase	phosphoglycerate kinase	-0.07	-0.16	0.02	-0.10	-0.13	0.06	-0.23	0.08	-0.69	-1.08
	cassava4.1_019184m.g	PS.calvin cycle	CP12 domain-containing protein 2	0.37	0.01	0.00	-1.28	0.16	-1.55	-0.35	-0.61	-0.79	-1.77
	cassava4.1_023305m.g	PS.calvin cycle.rubisco large subunit	ribulose-bisphosphate carboxylases	0.67	-0.34	-1.78	-0.56	-0.03	-0.72	-1.03	-1.53	-0.32	-2.29
	cassava4.1_005710m.g	PS.photorespiration.serine hydroxymethyltransferase	serine transhydroxymethyltransferase 1	-1.25	-3.78	-0.61	-4.50	0.01	-4.10	-0.12	-3.73	-0.92	-4.28
	cassava4.1_005814m.g	PS.photorespiration.serine hydroxymethyltransferase	serine transhydroxymethyltransferase 1	-1.16	-3.56	-0.34	-2.81	0.05	-3.22	-0.33	-2.73	-0.70	-2.98
	cassava4.1_008994m.g	PS.photorespiration.aminotransferases peroxisomal	alanine:glyoxylate aminotransferase	-1.05	-3.36	-0.77	-3.49	-0.09	-3.05	-0.19	-2.29	-0.57	-2.68
	cassava4.1_017913m.g	PS.photorespiration.glycine cleavage.H protein	Single hybrid motif superfamily protein	-0.98	-2.64	-1.00	-2.60	-0.10	-2.30	-0.69	-1.75	-1.05	-2.30
	cassava4.1_009477m.g	PS.photorespiration.hydroxypyruvate reductase	hydroxypyruvate reductase	-0.88	-2.85	-0.51	-2.71	-0.04	-2.81	-0.33	-2.24	-0.81	-2.88
	cassava4.1_011790m.g	PS.photorespiration	formyltetrahydrofolate deformylase, putative	-0.72	-2.09	-0.23	-1.09	-0.05	-1.99	-0.39	-1.87	-0.56	-1.86
	cassava4.1_008989m.g	PS.photorespiration.aminotransferases peroxisomal	alanine:glyoxylate aminotransferase	-0.69	-2.17	-0.67	-2.37	0.00	-1.82	-0.13	-1.84	-0.57	-2.53
	cassava4.1_010145m.g	PS.photorespiration.glycolate oxydase	Aldolase-type TIM barrel family protein	-0.67	-2.98	-0.33	-2.89	0.06	-2.87	-0.06	-1.82	-0.54	-2.51
	cassava4.1_010148m.g	PS.photorespiration.glycolate oxydase	Aldolase-type TIM barrel family protein	-0.64	-2.37	-0.52	-2.10	-0.14	-1.88	-0.27	-1.99	-0.74	-2.48
Photorespiration	cassava4.1_006274m.g	PS.photorespiration.glycerate kinase	P-loop containing nucleoside triphosphate hydrolases superfamily protein	-0.54	-1.45	-0.10	-1.45	0.05	-1.83	-0.19	-1.64	-0.61	-2.16
	cassava4.1_012207m.g	PS.photorespiration.hydroxypyruvate reductase	D-isomer specific 2-hydroxyacid dehydrogenase family protein	-0.42	-2.01	0.14	-2.17	0.15	-2.58	-0.46	-1.42	-0.58	-2.18
	cassava4.1_004233m.g	nitrite reductase 1	N-metabolism.nitrate metabolism.nitrite reductase	-2.35	-3.89	-0.98	-0.68	-0.31	-2.51	-1.02	-3.32	-0.51	-1.51
	cassava4.1_009848m.g	FMN-linked oxidoreductases superfamily protein	N-metabolism.misc	-1.18	-1.61	-0.16	-0.46	0.12	-0.41	-0.26	-2.46	-0.37	-1.36
	cassava4.1_008019m.g	glutamine synthetase 2	N-metabolism.ammonia metabolism.glutamine synthetase	-1.05	-2.83	-0.45	-2.12	-0.22	-2.78	-0.85	-2.59	-0.71	-2.54
	cassava4.1_000214m.g	glutamate synthase 1	N-metabolism.ammonia metabolism.glutamate synthase	-0.99	-2.47	-0.27	-2.31	-0.37	-2.45	0.10	-2.91	-0.78	-3.14
	cassava4.1_031400m.g	nitrate reductase 1	N-metabolism.nitrate metabolism.NR	-0.98	-2.84	-0.45	-1.49	-0.15	-3.11	-0.49	-3.61	-0.40	-2.09
	cassava4.1_007225m.g	nitrate reductase 1	N-metabolism.nitrate metabolism.NR	-0.84	-2.80	-0.64	-1.31	0.00	-2.69	-0.62	-3.58	-0.29	-1.46

	cassava4.1_003338m.g	nitroreductase family protein	N-metabolism.misc	-0.60	-1.13	-0.73	-1.46	-0.11	-0.79	0.21	-0.74	-0.41	-1.26
	cassava4.1_009228m.g	sulfite oxidase	N-metabolism.nitrate metabolism.NR	-0.52	-1.65	-0.06	-1.16	-0.23	-0.79	0.19	-2.88	-0.64	-2.49
	cassava4.1_000040m.g	NADH-dependent glutamate synthase 1	N-metabolism.ammonia metabolism.glutamate synthase	-0.30	-2.18	-0.20	-2.68	-0.36	-2.31	0.53	-1.75	-1.09	-3.48
	cassava4.1_010581m.g	glutamine synthase clone R1	N-metabolism.ammonia metabolism.glutamine synthetase	-0.26	0.09	-0.03	0.94	-0.09	1.32	-0.83	-0.62	-0.31	-0.33
	cassava4.1_000038m.g	NADH-dependent glutamate synthase 1	N-metabolism.ammonia metabolism.glutamate synthase	0.09	0.02	0.51	0.81	-0.38	0.89	0.97	0.24	0.24	0.81
	cassava4.1_001290m.g	signalling.in sugar and nutrient physiology	glutamate receptor 5	0.22	0.80	0.64	0.83	0.12	0.08	0.27	0.86	0.44	0.88
	cassava4.1_010597m.g	glutamine synthase clone R1	N-metabolism.ammonia metabolism.glutamine synthetase	0.29	0.27	0.01	0.19	-0.30	-0.07	-1.14	-0.59	-0.15	-0.51
Tetrapyrrole Synthesis	cassava4.1_028676m.g	tetrapyrrole synthesis.uroporphyrin-III C-methyltransferase	urophorphyrin methylase 1	-1.89	-1.78	0.44	1.49	-0.40	0.16	-1.40	-2.91	0.13	-0.44
	cassava4.1_010226m.g	tetrapyrrole synthesis.uroporphyrin-III C-methyltransferase	urophorphyrin methylase 1	-1.53	-2.13	0.22	0.45	-0.70	-0.50	-0.75	-2.86	-0.84	-1.25
	cassava4.1_027491m.g	tetrapyrrole synthesis.ALA dehydratase	Aldolase superfamily protein	-0.97	-2.63	0.19	-1.67	0.04	-2.49	-0.88	-2.32	-0.57	-2.29
	cassava4.1_031984m.g	tetrapyrrole synthesis.uroporphyrinogen III synthase	uroporphyrinogen-III synthase family protein	-0.95	-1.60	-0.62	-0.86	-0.36	-1.83	0.02	-1.67	-0.04	-1.13
	cassava4.1_013789m.g	tetrapyrrole synthesis.regulation	enzyme binding;tetrapyrrole binding	-0.92	-3.38	-0.56	-2.41	0.09	-1.99	-0.02	-1.70	-0.40	-2.54
	cassava4.1_000280m.g	tetrapyrrole synthesis.magnesium chelatase	magnesium-chelatase subunit chlH, chloroplast, putative / Mg-protoporphyrin IX chelatase, putative (CHLH)	-0.91	-2.90	-0.16	-2.19	-0.12	-2.38	-0.14	-2.19	-0.58	-2.66
	cassava4.1_002247m.g	tetrapyrrole synthesis.magnesium chelatase	ALBINA 1	-0.74	-1.35	-0.41	-1.00	-0.09	-1.72	-0.25	-1.02	0.04	-0.22
	cassava4.1_005000m.g	tetrapyrrole synthesis.glu-tRNA synthetase	Amidase family protein	-0.73	-1.70	-0.42	-1.29	-0.02	-0.77	-0.61	-0.74	-0.40	-0.86
	cassava4.1_008391m.g	tetrapyrrole synthesis.magnesium chelatase	P-loop containing nucleoside triphosphate hydrolases superfamily protein	-0.72	-1.50	-0.30	-1.04	-0.09	-1.33	-1.00	-1.08	-0.64	-1.09
	cassava4.1_004598m.g	tetrapyrrole synthesis.glu-tRNA synthetase	GLU-ADT subunit B	-0.72	-1.24	-0.01	-0.44	-0.18	-0.78	-0.68	-1.22	-0.37	-0.82
	cassava4.1_008382m.g	tetrapyrrole synthesis.magnesium chelatase	magnesium chelatase i2	-0.69	-1.46	0.12	-0.68	0.16	-1.51	-0.57	0.65	-0.16	0.19
	cassava4.1_008691m.g	tetrapyrrole synthesis.magnesium-protoporphyrin IX monomethyl ester (oxidative) cyclase	dicarboxylate diiron protein, putative (Crd1)	-0.64	-2.84	-0.25	-2.29	0.04	-2.36	-0.49	-1.80	-0.87	-2.33
	cassava4.1_009378m.g	tetrapyrrole synthesis.uroporphyrinogen decarboxylase	Uroporphyrinogen decarboxylase	-0.61	-1.30	-0.01	-1.24	0.07	-1.91	-0.66	-0.97	-0.50	-1.08
	assava4.1_009000m.g	tetrapyrrole synthesis.coproporphyrinogen III oxidase	Coproporphyrinogen III oxidase	-0.59	-0.85	-0.18	-0.46	-0.10	-0.29	-0.64	-0.70	-0.16	-0.43
	cassava4.1_008036m.g	tetrapyrrole synthesis.ALA dehydratase	Aldolase superfamily protein	-0.56	-1.17	-0.03	-0.82	-0.05	-0.81	-0.43	-0.78	-0.41	-0.78
	cassava4.1_008914m.g	tetrapyrrole synthesis.protochlorophyllide reductase	protochlorophyllide oxidoreductase C	-0.55	-2.06	-0.28	-2.33	0.00	-1.76	0.39	-1.58	-0.46	-1.93

	cassava4.1_006730m.g	tetrapyrrole synthesis.GSA	glutamate-1-semialdehyde 2,1-aminomutase 2	-0.52	-1.49	-0.08	-1.06	0.02	-0.91	-0.83	-0.43	-0.37	-0.52
	cassava4.1_005258m.g	tetrapyrrole synthesis.chlorophyll b synthase	Pheophorbide a oxygenase family protein with Rieske [2Fe-2S] domain	-0.49	-2.33	-0.19	-1.90	0.14	-2.25	-0.12	-1.93	-0.52	-2.15
	cassava4.1_004918m.g	tetrapyrrole synthesis.glu-tRNA reductase	Glutamyl-tRNA reductase family protein	-0.48	-0.76	1.04	1.07	0.35	0.52	-0.65	-1.12	-0.30	-0.51
	cassava4.1_013127m.g	tetrapyrrole synthesis.heme oxygenase	Plant haem oxygenase (decyclizing) family protein	-0.47	-0.73	-0.42	-0.94	-0.09	-0.51	0.00	-1.76	-0.85	-2.11
	cassava4.1_009920m.g	tetrapyrrole synthesis.chlorophyll synthase	UbiA prenyltransferase family protein	-0.46	-0.43	-0.21	-0.55	-0.10	-0.80	-0.28	-1.05	-0.09	-0.73
	cassava4.1_007874m.g	tetrapyrrole synthesis.ferrochelatase	ferrochelatase 2	-0.36	-1.53	-0.23	-0.89	0.00	-0.74	0.10	-0.47	-0.33	-0.74
	cassava4.1_011961m.g	tetrapyrrole synthesis.magnesium protoporphyrin IX methyltransferase	magnesium-protoporphyrin IX methyltransferase	-0.35	-2.00	0.16	-2.31	-0.04	-2.35	-0.66	-1.26	-0.26	-1.23
	cassava4.1_017765m.g	tetrapyrrole synthesis.uroporphyrinogen III synthase	uroporphyrinogen-III synthase family protein	-0.31	-1.19	-0.10	-0.95	0.06	-1.90	-0.38	-1.96	-0.94	-1.90
	cassava4.1_008498m.g	tetrapyrrole synthesis.divinyl chlorophyllide-a 8-vinyl-reductase	NAD(P)-binding Rossmann-fold superfamily protein	-0.29	-0.83	0.49	-0.42	-0.10	-1.36	0.19	-0.93	-0.61	-1.56
	cassava4.1_009063m.g	tetrapyrrole synthesis.protochlorophyllide reductase	protochlorophyllide oxidoreductase A	0.54	-0.30	0.42	-1.38	0.04	-2.21	-0.88	0.10	0.08	-0.58

Supplemental Table S5. Genes annotated to glycolysis, TCA, mitochondrial electron transport and flavonoid biosynthetic pathway.

The extent of differential expression is measured in terms of log2 fold change of treatment vs. control.

Table Class	Cassava ID	Mapman Category	Short Description	PG0	FG0	PG2	FG2	PG4	FG4	PG0	FG0	PG2	FG2
				120 day						45 day			
Glycolysis	cassava4.1_006973m.g	glycolysis.cytosolic branch.UGPase	UDP-glucose pyrophosphorylase 2	-0.46	-0.20	-0.35	-0.01	-0.31	0.07	-0.48	-0.78	-0.77	-0.99
	cassava4.1_006979m.g	glycolysis.cytosolic branch.UGPase	UDP-glucose pyrophosphorylase 2	0.90	2.14	0.73	1.79	0.30	2.29	0.20	1.60	0.42	1.77
	cassava4.1_003757m.g	glycolysis.cytosolic branch.phosphofructokinase (PFK)	Phosphofructokinase family protein	0.13	0.66	0.26	0.64	0.00	0.95	0.03	0.80	-0.20	0.67
	cassava4.1_003814m.g	glycolysis.cytosolic branch.phosphofructokinase (PFK)	Phosphofructokinase family protein	0.25	0.61	0.12	1.13	0.14	2.18	-0.81	1.48	0.24	1.72
	cassava4.1_004602m.g	glycolysis.cytosolic branch.phosphofructokinase (PFK)	Phosphofructokinase family protein	-0.12	0.03	0.06	0.41	0.09	0.96	-0.57	1.01	-0.02	0.88
	cassava4.1_014454m.g	glycolysis.cytosolic branch.triosephosphate isomerase (TPI)	triosephosphate isomerase	-1.01	-2.70	-0.35	-1.79	-0.28	-1.66	-0.55	-2.79	-0.88	-2.74
	cassava4.1_008220m.g	glycolysis.cytosolic branch.glyceraldehyde 3-phosphate dehydrogenase (GAP-DH)	glyceraldehyde-3-phosphate dehydrogenase of plastid 2	1.59	2.68	1.64	2.64	0.34	2.92	0.63	2.27	0.65	2.01
	cassava4.1_008225m.g	glycolysis.cytosolic branch.glyceraldehyde 3-phosphate dehydrogenase (GAP-DH)	glyceraldehyde-3-phosphate dehydrogenase of plastid 2	1.95	3.75	0.65	2.33	-0.18	3.72	-1.18	3.27	2.05	4.67
	cassava4.1_011176m.g	glycolysis.cytosolic branch.glyceraldehyde 3-phosphate dehydrogenase (GAP-DH)	glyceraldehyde-3-phosphate dehydrogenase C subunit 1	0.31	1.01	-0.17	0.37	-0.13	0.89	0.19	0.72	-0.22	0.29
	cassava4.1_011340m.g	glycolysis.cytosolic branch.glyceraldehyde 3-phosphate dehydrogenase (GAP-DH)	glyceraldehyde-3-phosphate dehydrogenase C subunit 1	-0.02	0.50	-0.06	0.44	-0.02	0.88	-0.60	0.43	-0.37	0.29
	cassava4.1_007673m.g	glycolysis.cytosolic branch.enolase	Enolase	0.22	0.34	0.24	0.75	0.16	1.08	0.31	1.04	0.03	0.70
	cassava4.1_006316m.g	glycolysis.cytosolic branch.pyruvate kinase (PK)	Pyruvate kinase family protein	0.29	1.19	0.75	2.61	0.05	2.03	0.22	0.67	0.70	2.41
	cassava4.1_005850m.g	glycolysis.cytosolic branch.pyruvate kinase (PK)	Pyruvate kinase family protein	0.30	0.80	0.53	1.25	-0.14	1.66	-0.40	0.61	-0.31	0.72
	cassava4.1_005893m.g	glycolysis.cytosolic branch.pyruvate kinase (PK)	Pyruvate kinase family protein	1.27	1.82	0.35	1.49	0.18	1.08	-0.44	2.10	-0.17	0.52
	cassava4.1_000725m.g	glycolysis.cytosolic branch.phospho-enol-pyruvate carboxylase (PEPC)	phosphoenolpyruvate carboxylase 4	0.08	-0.05	-0.05	-0.73	0.09	-0.31	0.30	-0.98	-0.05	-0.56
	cassava4.1_001047m.g	glycolysis.cytosolic branch.phospho-enol-pyruvate carboxylase (PEPC)	phosphoenolpyruvate carboxylase 1	-0.88	-1.80	-0.22	-1.18	-0.07	-0.96	-0.12	-2.21	-0.53	-1.84
	cassava4.1_003792m.g	glycolysis.plastid branch.phosphoglucomutase (PGM)	phosphoglucosamine mutase family protein	0.38	1.30	0.60	1.05	0.00	0.42	0.14	1.30	0.26	0.87

	cassava4.1_006414m.g	glycolysis.plastid branch.glucose-6-phosphate isomerase	phosphoglucose isomerase 1	1.55	2.39	1.14	1.65	0.25	0.64	-0.42	1.63	0.12	1.28
	cassava4.1_005217m.g	glycolysis.plastid branch.phosphofructokinase (PFK)	phosphofructokinase 5	1.12	1.68	0.88	1.78	0.23	2.05	-0.03	2.01	-0.07	1.77
	cassava4.1_005438m.g	glycolysis.plastid branch.phosphofructokinase (PFK)	phosphofructokinase 4	0.43	0.64	0.15	0.25	0.20	0.55	-0.49	1.22	0.26	0.91
	cassava4.1_005418m.g	glycolysis.plastid branch.pyruvate kinase (PK)	Pyruvate kinase family protein	0.66	1.11	-0.14	1.01	0.07	1.41	0.14	1.15	-0.27	0.24
	cassava4.1_004396m.g	glycolysis.plastid branch.pyruvate kinase (PK)	plastidic pyruvate kinase beta subunit 1	0.00	1.36	0.39	1.87	-0.10	2.37	-0.68	0.43	0.23	1.52
	cassava4.1_004405m.g	glycolysis.plastid branch.pyruvate kinase (PK)	plastidic pyruvate kinase beta subunit 1	0.25	1.14	0.08	0.91	0.23	1.49	-0.31	1.28	0.59	1.86
	cassava4.1_006316m.g	glycolysis.plastid branch.pyruvate kinase (PK)	Pyruvate kinase family protein	0.29	1.19	0.75	2.61	0.05	2.03	0.22	0.67	0.70	2.41
	cassava4.1_006063m.g	glycolysis.unclear/dually targeted.phosphoglycerate mutase	phosphoglycerate/bisphosphoglycerate mutase family protein	-0.01	-1.07	0.57	-0.38	0.06	-0.38	0.33	0.10	-0.44	-0.94
	cassava4.1_011851m.g	glycolysis.unclear/dually targeted.phosphoglycerate mutase	Phosphoglycerate mutase family protein	-0.19	0.24	-0.28	-1.10	0.01	-1.09	0.69	0.51	-0.15	-0.75
TCA	cassava4.1_007404m.g	TCA / org transformation.TCA.pyruvate DH.E1	pyruvate dehydrogenase complex E1 alpha subunit	0.39	0.78	0.61	1.23	0.08	1.09	-0.35	0.37	-0.24	0.86
	cassava4.1_007540m.g	TCA / org transformation.TCA.pyruvate DH.E1	pyruvate dehydrogenase complex E1 alpha subunit	0.48	1.00	0.34	1.07	-0.12	1.74	-0.31	1.07	-0.08	0.81
	cassava4.1_010116m.g	TCA / org transformation.TCA.pyruvate DH.E1	Transketolase family protein	0.66	1.23	0.48	1.30	0.14	0.83	-0.15	0.44	-0.05	0.75
	cassava4.1_010442m.g	TCA / org transformation.TCA.pyruvate DH.E1	Transketolase family protein	0.36	0.97	0.08	0.55	0.17	0.86	0.17	1.05	-0.11	0.70
	cassava4.1_004839m.g	TCA / org transformation.TCA.pyruvate DH.E2	Dihydrolipoamide acetyltransferase, long form protein	0.21	0.63	0.73	1.08	0.02	1.02	-0.01	0.96	0.03	0.95
	cassava4.1_004864m.g	TCA / org transformation.TCA.pyruvate DH.E2	Dihydrolipoamide acetyltransferase, long form protein	0.42	0.99	0.58	1.26	0.16	1.51	0.26	0.97	0.14	0.94
	cassava4.1_006906m.g	TCA / org transformation.TCA.pyruvate DH.E2	2-oxoacid dehydrogenases acyltransferase family protein	-0.20	0.55	0.10	0.52	0.18	1.10	-0.75	0.56	-0.09	0.63
	cassava4.1_004342m.g	TCA / org transformation.TCA.pyruvate DH.E3	lipoamide dehydrogenase 1	0.13	0.84	0.15	0.91	0.16	0.87	-0.53	0.94	0.24	1.48
	cassava4.1_005856m.g	TCA / org transformation.TCA.pyruvate DH.E3	mitochondrial lipoamide dehydrogenase 1	-0.84	-1.72	-0.49	-0.93	-0.25	-0.36	-0.50	-1.53	-0.70	-1.41
	cassava4.1_006853m.g	TCA / org transformation.TCA.CS	Citrate synthase family protein	0.33	0.84	0.07	0.78	0.02	0.98	-0.01	0.30	0.22	0.80
	cassava4.1_012522m.g	TCA / org transformation.TCA.CS	citrate synthase 5	1.05	1.55	0.51	0.85	0.11	1.21	-0.40	1.14	0.46	1.23
	cassava4.1_000903m.g	TCA / org transformation.TCA.aconitase	aconitase 3	0.69	1.54	0.62	1.52	-0.02	1.43	0.27	0.57	-0.13	0.60
	cassava4.1_000908m.g	TCA / org transformation.TCA.aconitase	aconitase 3	0.47	0.90	0.35	0.68	0.06	0.87	0.29	0.96	-0.03	0.47
	cassava4.1_001348m.g	TCA / org transformation.TCA.aconitase	aconitase 1	1.23	2.37	0.83	1.66	-0.06	1.18	0.33	2.29	0.70	2.14
	cassava4.1_005842m.g	TCA / org transformation.TCA.aconitase	isopropyl malate isomerase large subunit 1	-0.64	-0.63	0.03	0.06	0.04	0.17	-0.49	-0.95	-0.29	-0.61
	cassava4.1_005859m.g	TCA / org transformation.TCA.aconitase	isopropyl malate isomerase large subunit 1	-0.40	-1.15	-0.10	-0.48	-0.22	-0.01	-0.60	-1.77	-0.89	-1.61
	cassava4.1_033921m.g	TCA / org transformation.TCA.aconitase	aconitase 3	-0.36	1.59	-1.73	-0.89	-0.93	2.23	2.08	2.78	0.17	2.85

	cassava4.1_007405m.g	TCA / org transformation.TCA.IDH	Isocitrate/isopropylmalate dehydrogenase family protein	-0.24	0.17	0.13	0.63	0.32	1.11	-0.40	1.87	0.21	1.84
	cassava4.1_009195m.g	TCA / org transformation.TCA.IDH	cytosolic NADP+-dependent isocitrate dehydrogenase	1.58	2.52	0.97	2.08	0.18	2.63	-0.23	1.87	0.56	2.60
	cassava4.1_009952m.g	TCA / org transformation.TCA.IDH	isocitrate dehydrogenase III	0.68	0.43	0.47	0.68	0.05	0.83	-0.26	0.58	0.00	0.90
	cassava4.1_008387m.g	TCA / org transformation.TCA.succinyl-CoA ligase	ATP citrate lyase (ACL) family protein	0.25	0.56	0.21	0.85	-0.04	1.22	-0.17	0.41	-0.03	0.53
	cassava4.1_010959m.g	TCA / org transformation.TCA.succinyl-CoA ligase	Succinyl-CoA ligase, alpha subunit	0.41	0.85	0.19	0.89	0.14	1.11	-0.38	1.17	0.53	1.58
	cassava4.1_034079m.g	TCA / org transformation.TCA.succinyl-CoA ligase	Succinyl-CoA ligase, alpha subunit	0.20	0.67	-0.06	0.55	0.53	1.41	-0.70	1.42	-0.13	1.29
	cassava4.1_003519m.g	TCA / org transformation.TCA.succinate dehydrogenase	succinate dehydrogenase 1-1	-0.41	-0.23	-0.23	-0.02	-0.07	0.88	-0.04	-0.03	-0.19	0.21
	cassava4.1_013361m.g	TCA / org transformation.TCA.succinate dehydrogenase	succinate dehydrogenase 2-2	0.65	0.76	0.41	0.78	0.19	1.20	-0.22	1.24	0.23	0.86
	cassava4.1_034460m.g	TCA / org transformation.TCA.malate DH	malate dehydrogenase	0.57	0.77	0.02	0.46	0.08	1.44	-1.19	1.01	-0.62	0.76
	cassava4.1_013362m.g	TCA / org transformation.TCA.succinate dehydrogenase	succinate dehydrogenase 2-2	0.75	1.51	0.12	0.50	0.09	1.34	0.69	1.40	-0.38	0.73
	cassava4.1_011133m.g	TCA / org transformation.TCA.malate DH	Lactate/malate dehydrogenase family protein	-0.02	0.10	0.25	0.63	0.13	1.04	-0.36	0.30	-0.43	0.13
mitochondrial electron transport	cassava4.1_010822m.g	mitochondrial electron transport / ATP synthesis.alternative oxidase	alternative oxidase 2	-0.41	-0.90	-0.19	-0.58	-0.04	-0.40	0.08	-0.56	-0.49	-0.72
	cassava4.1_027944m.g	mitochondrial electron transport / ATP synthesis.cytochrome c	transmembrane protein G1P-related 1	-0.37	-0.03	0.13	0.25	0.30	1.39	-0.97	0.52	-0.94	-0.02
	cassava4.1_010184m.g	mitochondrial electron transport / ATP synthesis.NADH-DH.type II.mitochondrial	NAD(P)H dehydrogenase C1	-0.33	-1.00	-0.57	-0.62	0.08	-0.55	-0.39	-0.69	-0.34	-0.86
	cassava4.1_016672m.g	mitochondrial electron transport / ATP synthesis.NADH-DH.complex I	NADPH:quinone oxidoreductase	-0.21	-1.50	-0.44	-1.10	-0.13	-0.25	-0.20	-0.25	-0.46	-0.32
	cassava4.1_013530m.g	mitochondrial electron transport / ATP synthesis.cytochrome c reductase	Ubiquinol-cytochrome C reductase iron-sulfur subunit	-0.06	0.01	-0.05	0.25	0.04	0.93	-0.45	-0.60	-0.62	-0.21
	cassava4.1_017064m.g	mitochondrial electron transport / ATP synthesis.cytochrome c oxidase	cytochrome C oxidase 6B	-0.05	-0.15	-0.13	0.16	-0.02	0.90	-0.33	0.77	-0.01	0.56
	cassava4.1_002356m.g	mitochondrial electron transport / ATP synthesis.NADH-DH.localisation not clear	NADH-ubiquinone dehydrogenase, mitochondrial, putative	0.04	-0.08	0.12	0.12	0.09	0.86	-0.03	0.23	-0.20	0.03
	cassava4.1_012361m.g	mitochondrial electron transport / ATP synthesis.cytochrome c reductase	Ubiquinol-cytochrome C reductase iron-sulfur subunit	0.05	0.13	0.11	0.35	0.00	1.22	-0.02	0.22	-0.28	0.16
	cassava4.1_004343m.g	mitochondrial electron transport / ATP synthesis.NADH-DH.type II.external	NAD(P)H dehydrogenase B1	0.11	-0.03	0.17	-0.23	-0.01	-0.21	0.17	-0.31	-0.38	-0.77
	cassava4.1_013140m.g	mitochondrial electron transport / ATP synthesis.cytochrome c oxidase	unknow	0.11	0.08	0.06	-0.49	0.20	-1.11	-0.38	0.11	-0.32	-0.37
	cassava4.1_004630m.g	mitochondrial electron transport / ATP synthesis.F1-ATPase	ATP synthase alpha/beta family protein	0.13	0.32	0.16	0.52	0.10	1.03	-0.51	0.56	-0.03	0.68
	cassava4.1_016028m.g	mitochondrial electron transport / ATP synthesis.NADH-DH.localisation not clear	NADH-ubiquinone oxidoreductase 20 kDa subunit, mitochondrial	0.14	-0.10	0.08	0.33	0.03	1.14	-0.01	-0.09	-0.16	0.23
	cassava4.1_013120m.g	mitochondrial electron transport / ATP synthesis.NADH-DH.complex I	prohibitin 6	0.15	0.68	0.20	0.82	0.18	1.12	-0.69	1.61	-0.10	1.16
	cassava4.1_017764m.g	mitochondrial electron transport / ATP synthesis.cytochrome c oxidase	Rubredoxin-like superfamily protein	0.16	0.01	0.19	0.57	0.03	0.98	-0.20	0.22	0.18	0.85

	cassava4.1_017840m.g	mitochondrial electron transport / ATP synthesis.NADH-DH.localisation not clear	NADH-ubiquinone oxidoreductase-related	0.17	0.78	0.10	0.88	0.28	1.34	-0.25	1.51	-0.19	1.41
	cassava4.1_019324m.g	mitochondrial electron transport / ATP synthesis.F1-ATPase	Mitochondrial ATP synthase subunit G protein	0.19	0.85	0.46	0.91	0.15	1.41	-0.14	0.81	-0.10	0.51
	cassava4.1_014720m.g	mitochondrial electron transport / ATP synthesis.NADH-DH.complex I.carbonic anhydrase	gamma carbonic anhydrase like 1	0.19	0.22	0.15	0.42	-0.09	1.10	-0.23	1.19	0.25	1.20
	cassava4.1_004726m.g	mitochondrial electron transport / ATP synthesis.F1-ATPase	ATP synthase alpha/beta family protein	0.20	0.53	0.23	0.63	0.01	0.97	-0.07	0.38	-0.08	0.38
	cassava4.1_014473m.g	mitochondrial electron transport / ATP synthesis.NADH-DH.localisation not clear	NADH-ubiquinone oxidoreductase 24 kDa subunit, putative	0.23	0.28	0.22	0.58	0.22	0.92	-0.31	0.79	0.06	0.82
	cassava4.1_012332m.g	mitochondrial electron transport / ATP synthesis.NADH-DH.localisation not clear	NADH-ubiquinone oxidoreductase 24 kDa subunit, putative	0.24	0.52	0.28	0.55	-0.26	0.60	-0.45	0.59	0.05	0.89
	cassava4.1_020733m.g	mitochondrial electron transport / ATP synthesis.F1-ATPase	ATP synthase epsilon chain, mitochondrial	0.25	0.53	0.10	0.66	0.20	1.21	-0.24	1.19	0.09	1.24
	cassava4.1_019319m.g	mitochondrial electron transport / ATP synthesis.F1-ATPase	Mitochondrial ATP synthase subunit G protein	0.27	0.76	0.36	0.95	0.02	1.03	-0.43	0.29	0.00	0.39
	cassava4.1_016505m.g	mitochondrial electron transport / ATP synthesis.cytochrome c oxidase	electron transport SCO1/SenC family protein	0.28	1.11	0.02	0.54	0.00	0.79	-0.26	2.22	0.44	1.67
	cassava4.1_013823m.g	mitochondrial electron transport / ATP synthesis.NADH-DH.complex I.carbonic anhydrase	gamma carbonic anhydrase 1	0.29	0.52	0.40	0.55	-0.14	0.91	-0.31	0.76	-0.12	0.61
	cassava4.1_014599m.g	mitochondrial electron transport / ATP synthesis.electron transfer flavoprotein	electron transfer flavoprotein beta	0.29	0.87	-0.09	0.26	0.19	0.32	0.36	1.71	-0.04	0.80
	cassava4.1_017212m.g	mitochondrial electron transport / ATP synthesis.cytochrome c oxidase	cytochrome C oxidase 6B	0.29	0.88	0.28	1.03	-0.26	1.05	-1.02	0.76	0.47	1.75
	cassava4.1_019654m.g	mitochondrial electron transport / ATP synthesis.cytochrome c	cytochrome c-2	0.31	0.55	0.77	1.50	0.05	1.84	0.05	1.79	0.10	1.30
	cassava4.1_020694m.g	mitochondrial electron transport / ATP synthesis.cytochrome c reductase	Cytochrome b-c1 complex, subunit 8 protein	0.31	0.50	0.18	0.61	0.09	1.10	-0.46	0.68	-0.21	0.55
	cassava4.1_006409m.g	mitochondrial electron transport / ATP synthesis.F1-ATPase	ATPase, V1 complex, subunit B protein	0.32	0.86	0.45	1.01	0.19	0.75	-0.72	0.69	-0.14	0.78
	cassava4.1_017771m.g	mitochondrial electron transport / ATP synthesis.F1-ATPase	ATP synthase D chain, mitochondrial	0.33	0.91	0.31	0.77	0.10	1.06	-0.13	0.86	-0.26	0.57
	cassava4.1_006064m.g	mitochondrial electron transport / ATP synthesis.NADH-DH.type II.internal matrix	alternative NAD(P)H dehydrogenase 2	0.33	0.28	0.50	0.56	-0.06	0.02	0.19	0.54	0.57	1.26
	cassava4.1_019945m.g	mitochondrial electron transport / ATP synthesis.cytochrome c oxidase	cytochrome c oxidase-related	0.40	0.40	0.06	0.56	-0.03	1.29	-0.61	0.68	-0.22	0.87
	cassava4.1_013108m.g	mitochondrial electron transport / ATP synthesis.NADH-DH.complex I	prohibitin 1	0.41	0.83	0.20	0.68	0.17	1.04	-0.78	2.39	0.29	2.03
	cassava4.1_014824m.g	mitochondrial electron transport / ATP synthesis.uncoupling protein	plant uncoupling mitochondrial protein 1	0.43	1.05	0.10	0.94	0.43	1.26	-0.23	1.37	-0.08	1.07
	cassava4.1_020696m.g	mitochondrial electron transport / ATP synthesis.cytochrome c reductase	ubiquinol-cytochrome C reductase UQCRX/QCR9-like family protein	0.43	0.68	0.39	0.90	-0.06	0.81	-0.58	0.78	0.12	1.15

	cassava4.1_016521m.g	mitochondrial electron transport / ATP synthesis.F1-ATPase	ATPase, F1 complex, delta/epsilon subunit	0.45	0.75	0.25	0.67	0.17	1.50	-0.16	0.67	-0.24	0.50
	cassava4.1_019932m.g	mitochondrial electron transport / ATP synthesis.NADH-DH.localisation not clear	NADH-ubiquinone oxidoreductase B18 subunit, putative	0.47	0.59	0.20	0.76	0.10	1.16	-0.49	0.83	-0.06	1.14
	cassava4.1_013697m.g	mitochondrial electron transport / ATP synthesis.cytochrome c reductase	Ubiquinol-cytochrome C reductase iron-sulfur subunit	0.47	0.80	0.58	0.70	0.23	1.35	-0.70	0.79	-0.01	1.01
	cassava4.1_019624m.g	mitochondrial electron transport / ATP synthesis.cytochrome c	cytochrome c-2	0.49	0.70	0.64	1.30	-0.15	1.98	-0.98	0.33	-0.21	0.92
	cassava4.1_010295m.g	mitochondrial electron transport / ATP synthesis.cytochrome c oxidase	Surfeit locus 1 cytochrome c oxidase biogenesis protein	0.54	0.91	0.03	0.74	0.04	1.07	0.18	1.42	-0.39	1.13
	cassava4.1_005184m.g	mitochondrial electron transport / ATP synthesis.NADH-DH.type II.internal matrix	alternative NAD(P)H dehydrogenase 1	0.63	0.83	0.18	0.86	-0.04	1.31	-1.26	0.78	-0.56	1.20
	cassava4.1_010697m.g	mitochondrial electron transport / ATP synthesis.electron transfer flavoprotein	electron transfer flavoprotein alpha	0.68	1.39	0.07	0.42	0.04	1.04	-0.17	1.30	0.17	1.09
	cassava4.1_004741m.g	mitochondrial electron transport / ATP synthesis.NADH-DH.type II.external	NAD(P)H dehydrogenase B2	0.70	0.30	-0.18	-0.14	0.06	0.80	0.03	1.86	-0.76	-1.18
	cassava4.1_019695m.g	mitochondrial electron transport / ATP synthesis.NADH-DH.localisation not clear	NADH:ubiquinone oxidoreductase, 17.2kDa subunit	0.74	0.44	-0.29	1.05	0.09	1.30	0.33	1.39	1.09	2.14
	cassava4.1_009351m.g	transport.metabolite transporters at the mitochondrial membrane	ADP/ATP carrier 2	0.37	0.51	0.51	0.89	0.19	0.90	-0.93	0.04	-0.10	0.59
	cassava4.1_009354m.g	transport.metabolite transporters at the mitochondrial membrane	ADP/ATP carrier 2	0.10	0.40	0.84	1.56	0.07	1.81	-0.65	0.37	-0.12	1.03
	cassava4.1_011745m.g	transport.metabolite transporters at the mitochondrial membrane	uncoupling protein 5	0.38	-1.46	1.75	1.08	0.41	0.44	-2.26	0.12	0.62	-0.48
	cassava4.1_023265m.g	mitochondrial electron transport / ATP synthesis.electron transfer flavoprotein	electron-transfer flavoprotein:ubiquinone oxidoreductase	0.80	1.67	0.52	0.56	0.00	0.40	0.68	1.28	0.44	0.80
	cassava4.1_017914m.g	mitochondrial electron transport / ATP synthesis.cytochrome c oxidase	Rubredoxin-like superfamily protein	0.81	0.75	0.85	1.22	0.08	1.42	-0.01	1.34	0.57	1.60
	cassava4.1_014864m.g	mitochondrial electron transport / ATP synthesis.NADH-DH.localisation not clear	DERLIN-1	0.85	0.79	0.48	0.50	-0.10	-0.02	1.63	1.17	0.35	0.37
Flavonoid Biosynthetic	cassava4.1_004658m.g	4CL3; 4-coumarate-CoA ligase	4-coumarate:CoA ligase 3	0.27	1.03	1.04	3.06	-0.39	4.68	-0.15	0.91	-0.12	2.48
	cassava4.1_000031m.g	lipid metabolism.FA synthesis and FA elongation.Acetyl CoA Carboxylation.homomeric Enzyme	acetyl-CoA carboxylase 1 (ACC1)	0.82	2.10	0.61	2.16	0.23	2.20	1.30	1.60	0.43	1.85
	cassava4.1_000041m.g	lipid metabolism.FA synthesis and FA elongation.Acetyl CoA Carboxylation.homomeric Enzyme	acetyl-CoA carboxylase 1 (ACC1)	0.47	1.49	0.67	1.92	-0.11	2.21	0.92	0.34	-0.07	0.76
	cassava4.1_010685m.g	secondary metabolism.flavonoids.dihydroflavonols.dihydroflavonol 4-reductase	dihydroflavonol 4-reductase (DFR)	2.65	4.49	4.37	6.95	0.50	7.40	-2.31	6.64	4.10	9.30
	cassava4.1_010212m.g	hormone metabolism.ethylene.synthesis-degradation	flavanone 3-hydroxylase (F3H)	3.44	5.05	4.32	6.42	0.20	7.71	-1.61	5.35	2.87	7.69
	cassava4.1_011534m.g	hormone metabolism.ethylene.synthesis-degradation	flavonol synthase 1 (FLS1)	1.37	2.75	2.59	4.59	0.17	4.60	-1.49	3.55	1.09	5.63

	cassava4.1_010809m.g	hormone metabolism.ethylene.synthesis-degradation	leucoanthocyanidin dioxygenase (LDOX)	2.38	3.20	4.43	6.31	0.34	7.60	-3.74	5.40	3.36	8.59
	cassava4.1_008985m.g	RNA.regulation of transcription.MYB domain transcription factor family	myb domain protein 111 (MYB111)	0.98	0.80	1.32	0.68	-0.78	1.73	0.32	2.04	1.28	2.27
	cassava4.1_009295m.g	secondary metabolism.flavonoids.chalcones	TRANSPARENT TESTA 4 (TT4)	1.62	4.03	2.37	5.46	0.40	7.37	-1.77	3.38	0.61	5.50
	cassava4.1_009206m.g	secondary metabolism.flavonoids.chalcones	TRANSPARENT TESTA 4 (TT4)	2.51	4.35	3.43	5.94	-0.08	7.97	-3.30	2.01	1.86	6.09
	cassava4.1_009402m.g	secondary metabolism.flavonoids.chalcones	TRANSPARENT TESTA 4 (TT4)	3.06	4.46	5.26	7.21	0.46	7.99	-4.08	5.66	3.89	9.29
	cassava4.1_007396m.g	secondary metabolism.flavonoids.chalcones	TRANSPARENT TESTA 5 (TT5)	1.03	2.27	1.20	3.03	0.22	3.56	-0.77	1.59	0.38	3.56
	cassava4.1_005974m.g	misc.cytochrome P450	TRANSPARENT TESTA 7 (TT7);	1.93	2.73	4.94	6.92	0.33	7.79	-0.59	7.22	3.54	8.89
	cassava4.1_005668m.g	misc.cytochrome P450	TRANSPARENT TESTA 7 (TT7);	2.19	3.84	1.94	4.34	0.22	4.54	-1.83	1.75	0.96	4.59
cytochrome B5	cassava4.1_018938m.g	cytochrome	cytochrome B5 isoform E	1.27	2.19	0.34	0.76	0.08	1.14	0.37	-0.21	-0.33	0.27
	cassava4.1_018863m.g	cytochrome	cytochrome B5 isoform B	-0.15	0.04	0.09	0.78	-0.03	0.92	-0.45	-0.31	-0.59	-0.42
	cassava4.1_018955m.g	cytochrome	cytochrome B5 isoform B	0.61	1.02	0.44	1.10	0.29	0.98	-1.22	0.58	-0.31	1.12
	cassava4.1_018882m.g	cytochrome	cytochrome B5 isoform B	0.31	0.63	0.21	0.64	-0.28	1.38	-0.79	0.34	-0.53	0.57
	cassava4.1_018854m.g	cytochrome	cytochrome B5 isoform B	0.94	1.65	0.89	1.30	0.14	2.17	-1.38	0.52	-0.22	0.53
	cassava4.1_027275m.g	cytochrome	cytochrome B5 isoform D	2.85	4.14	4.58	6.77	0.61	8.35	-3.19	4.41	2.94	7.91

Supplemental Table S6. Genes annotated to starch and sucrose metabolism related genes. The extent of differential expression is measured in terms of log2 fold change of treatment vs. control.

Cassava ID	Mapman Category	Short Description	PG0	FG0	PG2	FG2	PG4	FG4	PG0	FG0	PG2	FG2
			120 day						45 day			
cassava4.1_005409m.g	major CHO metabolism.synthesis.starch.AGPase	glucose-1-phosphate adenylyltransferase	2.31	3.39	1.70	2.76	0.32	2.01	0.24	3.35	0.84	2.53
cassava4.1_005518m.g	major CHO metabolism.synthesis.starch.AGPase	glucose-1-phosphate adenylyltransferase	2.71	3.05	2.11	2.82	0.31	1.22	-0.77	4.41	1.46	3.81
cassava4.1_021267m.g	major CHO metabolism.synthesis.starch.AGPase	highly similar to (674) AT1G27680 | Symbols: APL2 | APL2 (ADPGLC-PPASE LARGE SUBUNIT); glucose-1-phosphate adenylyltransferase	0.03	0.53	0.90	1.59	-0.23	1.05	-0.36	1.92	-0.03	2.26
cassava4.1_005507m.g	major CHO metabolism.synthesis.starch.AGPase	highly similar to (855) AT5G19220 | Symbols: ADG2, APL1 | APL1 (ADP GLUCOSE PYROPHOSPHORYLASE LARGE SUBUNIT 1); glucose-1-phosphate adenylyltransferase	-0.91	-2.90	-0.19	-2.72	-0.02	-3.77	-0.30	-2.39	-0.62	-2.70
cassava4.1_002278m.g	major CHO metabolism.synthesis.starch.starch synthase	nearly identical (1009) AT3G01180 | Symbols: AtSS2 | AtSS2 (starch synthase 2); transferase, transferring glycosyl groups	-0.07	-1.64	0.03	-1.41	-0.12	-0.89	0.03	-1.26	-0.43	-1.66
cassava4.1_003884m.g	major CHO metabolism.synthesis.starch.starch synthase	highly similar to (894) AT1G32900 | Symbols: | starch synthase, putative	-0.97	-2.77	-0.01	-1.37	-0.05	-1.30	-0.69	-2.18	-0.75	-2.68
cassava4.1_004619m.g	major CHO metabolism.synthesis.starch.starch synthase	highly similar to (885) AT5G24300 | Symbols: SSI1, SSI, ATSS1 | SSI1 (SUPPRESSOR OF SALICYLIC ACID INSENSITIVITY 1); starch synthase/ transferase, transferring glycosyl groups	-0.28	-0.12	-0.35	-0.84	-0.13	-0.72	0.20	-0.33	-0.22	-0.59
cassava4.1_001595m.g	major CHO metabolism.synthesis.starch.starch branching	highly similar to (832) AT5G03650 | Symbols: SBE2.2 | SBE2.2 (starch branching enzyme 2.2); 1,4-alpha-glucan branching enzyme	0.29	0.88	0.69	0.97	0.14	1.17	-0.33	1.72	0.44	1.79
cassava4.1_001686m.g	major CHO metabolism.synthesis.starch.starch branching	nearly identical (1303) AT5G03650 | Symbols: SBE2.2 | SBE2.2 (starch branching enzyme 2.2); 1,4-alpha-glucan branching enzyme	0.54	0.71	0.84	1.05	0.17	0.11	0.42	1.71	0.26	1.08
cassava4.1_003773m.g	major CHO metabolism.synthesis.starch.starch branching	nearly identical (1128) AT5G03650 | Symbols: SBE2.2 | SBE2.2 (starch branching enzyme 2.2); 1,4-alpha-glucan branching enzyme	0.91	1.42	1.05	1.23	0.30	0.24	0.40	3.40	0.95	2.50
cassava4.1_001096m.g	major CHO metabolism.degradation.starch.starch phosphorylase	nearly identical (1369) AT3G29320 | Symbols: | glucan phosphorylase, putative	-0.51	-1.13	0.24	-0.55	-0.13	-1.71	-0.56	0.01	-0.19	0.37

cassava4.1_001626m.g	major CHO metabolism.degradation.starch.starch phosphorylase	nearly identical (1470) AT3G46970 | Symbols: ATPHS2, PHS2 | PHS2 (ALPHA-GLUCAN PHOSPHORYLASE 2); phosphorylase/ transferase, transferring glycosyl groups	1.46	2.17	1.48	2.15	0.27	1.10	0.01	3.01	0.71	2.66
cassava4.1_002466m.g	major CHO metabolism.degradation.starch.starch phosphorylase	highly similar to (778) AT3G29320 | Symbols: | glucan phosphorylase, putative	1.23	2.09	0.71	1.42	0.35	0.98	-0.86	2.88	0.77	2.93
cassava4.1_002614m.g	major CHO metabolism.degradation.starch.starch phosphorylase	highly similar to (764) AT3G29320 | Symbols: | glucan phosphorylase, putative	0.69	1.54	0.76	1.07	0.36	1.00	0.06	3.77	0.67	3.12
cassava4.1_004717m.g	major CHO metabolism.degradation.starch.starch phosphorylase	highly similar to (739) AT3G29320 | Symbols: | glucan phosphorylase, putative	0.97	1.94	1.17	1.54	0.30	1.02	-0.18	3.45	0.33	2.64
cassava4.1_000266m.g	major CHO metabolism.degradation.starch.glucan water dikinase	nearly identical (1917) AT1G10760 | Symbols: SEX1, SOP1, SOP, GWD1, GWD | SEX1 (STARCH EXCESS 1); alpha-glucan, water dikinase	0.44	1.31	0.79	1.82	0.08	1.53	0.26	1.29	0.82	1.72
cassava4.1_000497m.g	major CHO metabolism.degradation.starch.glucan water dikinase	nearly identical (1490) AT5G26570 | Symbols: PWD, OK1, ATGWD3 | ATGWD3; carbohydrate kinase/ catalytic/ phosphoglucan, water dikinase	0.24	0.69	0.15	-0.28	0.06	-0.54	0.51	0.86	0.18	-0.13
cassava4.1_006053m.g	major CHO metabolism.degradation.starch.starch cleavage	highly similar to (627) AT5G11720 | Symbols: | alpha-glucosidase 1 (AGLU1)	0.52	0.93	-0.20	0.26	0.32	1.93	1.18	2.60	1.44	2.90
cassava4.1_001362m.g	major CHO metabolism.degradation.starch.starch cleavage.alpha amylase	nearly identical (1236) AT1G69830 | Symbols: ATAMY3, AMY3 | AMY3 (ALPHA-AMYLASE-LIKE 3); alpha-amylase	0.70	1.27	-0.63	-0.31	-0.12	-0.95	0.60	0.15	-0.27	-0.47
cassava4.1_008754m.g	major CHO metabolism.degradation.starch.starch cleavage.alpha amylase	highly similar to (708) AT1G76130 | Symbols: ATAMY2, AMY2 | AMY2 (ALPHA-AMYLASE-LIKE 2); alpha-amylase/ calcium ion binding / catalytic/ cation binding	0.81	1.47	0.41	1.18	-0.08	1.47	-0.66	0.85	-0.57	0.50
cassava4.1_004345m.g	major CHO metabolism.degradation.starch.starch cleavage.beta amylase	highly similar to (876) AT3G23920 | Symbols: BAM1, BMY7, TR-BAMY | BAM1 (BETA-AMYLASE 1); beta-amylase	-0.12	-1.15	-0.12	-1.58	-0.09	-0.50	0.99	0.08	-0.42	-1.12
cassava4.1_005239m.g	major CHO metabolism.degradation.starch.starch cleavage.beta amylase	highly similar to (582) AT5G18670 | Symbols: BMY3, BAM9 | BMY3; beta-amylase/ catalytic/ cation binding	-0.53	-1.42	-0.50	-0.53	-0.01	-0.05	0.34	-1.18	-1.00	-1.80
cassava4.1_032401m.g	major CHO metabolism.degradation.starch.starch cleavage.beta amylase	moderately similar to (376) AT4G17090 | Symbols: CT-BMY, BAM3, BMY8 | CT-BMY (CHLOROPLAST BETA-AMYLASE);	1.26	1.94	2.31	3.67	0.49	1.89	1.48	3.47	0.04	2.71

		beta-amylase										
cassava4.1_034364m.g	major CHO metabolism.degradation.starch.starch cleavage.beta amylase	moderately similar to (332) AT4G17090 | Symbols: CT-BMY, BAM3, BMY8 | CT-BMY (CHLOROPLAST BETA-AMYLASE)	-0.66	-2.41	-0.36	-1.30	-0.07	-1.25	-0.65	-1.33	-0.59	-2.20
cassava4.1_008825m.g	transport.metabolite transporters at the envelope membrane	highly similar to (573) AT5G46110 | Symbols: APE2, TPT | APE2 (ACCLIMATION OF PHOTOSYNTHESIS TO ENVIRONMENT 2)	-0.64	-2.09	-0.28	-1.28	-0.06	-1.55	-0.38	-1.88	-0.61	-1.85
cassava4.1_000732m.g	major CHO metabolism.synthesis.sucrose.SPS	nearly identical (1669) AT5G20280 | Symbols: ATSPS1F | ATSPS1F (sucrose phosphate synthase 1F)	0.08	1.35	0.12	0.15	0.05	-0.06	0.44	-0.22	-0.05	-0.21
cassava4.1_000827m.g	major CHO metabolism.synthesis.sucrose.SPS	nearly identical (1521) AT4G10120 | Symbols: ATSPS4F | ATSPS4F; transferase, transferring glycosyl groups	-0.20	-0.52	0.00	-0.13	0.18	-1.18	0.45	0.94	0.62	0.72
cassava4.1_000839m.g	major CHO metabolism.synthesis.sucrose.SPS	nearly identical (1527) AT4G10120 | Symbols: ATSPS4F | ATSPS4F; transferase, transferring glycosyl groups	-0.84	-2.25	-0.43	-1.12	-0.26	-1.93	-0.34	-2.64	-0.11	-2.10
cassava4.1_024105m.g	major CHO metabolism.synthesis.sucrose.SPS	nearly identical (1583) AT1G04920 | Symbols: ATSPS3F | ATSPS3F (sucrose phosphate synthase 3F); sucrose-phosphate synthase/ transferase, transferring glycosyl groups	-0.20	-2.22	0.00	-1.85	-0.30	-3.28	0.41	-2.17	-0.35	-2.22
cassava4.1_008177m.g	major CHO metabolism.synthesis.sucrose.SPP	highly similar to (621) AT2G35840 | Symbols: | sucrose-phosphatase 1 (SPP1)	0.23	0.14	0.18	0.50	0.14	0.66	-0.08	0.89	-0.45	0.20
cassava4.1_008254m.g	major CHO metabolism.synthesis.sucrose.SPP	highly similar to (626) AT2G35840 | Symbols: | sucrose-phosphatase 1 (SPP1)	-0.29	-0.23	0.31	0.40	-0.05	-0.11	-0.23	-1.15	-0.54	-1.03
cassava4.1_004111m.g	transport.sugars.sucrose	highly similar to (620) AT1G22710 | Symbols: SUC2, SUT1, ATSUC2 | SUC2 (SUCROSE-PROTON SYMPORTER 2)	0.80	1.66	-0.18	-0.35	-0.13	0.77	-0.13	0.24	-0.30	-0.25
cassava4.1_006202m.g	transport.sugars.sucrose	highly similar to (628) AT1G09960 | Symbols: SUT4, ATSUT4, suc4 | SUT4 (SUCROSE TRANSPORTER 4)	0.71	0.94	0.09	0.80	0.02	0.78	0.09	0.76	-0.08	0.15
cassava4.1_006880m.g	transport.sugars.sucrose	highly similar to (609) AT1G09960 | Symbols: SUT4, ATSUT4, suc4 | SUT4 (SUCROSE TRANSPORTER 4)	-0.69	-1.23	-0.32	-0.29	0.12	0.41	0.33	0.59	0.30	0.16
cassava4.1_007460m.g	transport.sugars.sucrose	highly similar to (536) AT1G22710 | Symbols: SUC2, SUT1, ATSUC2 | SUC2 (SUCROSE-PROTON SYMPORTER 2)	0.55	0.93	-0.18	0.46	-0.18	1.64	0.29	0.83	-0.63	0.71
cassava4.1_021570m.g	transport.sugars	MSS1; carbohydrate transmembrane transporter/ hexose:hydrogen	1.87	2.84	0.08	1.09	0.74	2.50	0.26	2.17	1.74	2.78

		symporter/ high-affinity hydrogen:glucose symporter/ sugar:hydrogen symporter										
cassava4.1_002913m.g	major CHO metabolism.degradation.sucrose.invertases.neutral	highly similar to (980) AT3G06500 | Symbols: | beta-fructofuranosidase, putative / invertase, putative / saccharase, putative / beta-fructosidase, putative	0.44	0.51	1.09	1.68	0.28	1.44	0.67	0.66	-0.49	0.85
cassava4.1_005201m.g	major CHO metabolism.degradation.sucrose.invertases.neutral	nearly identical (1003) AT4G09510 | Symbols: CINV2 | beta-fructofuranosidase, putative / invertase, putative / saccharase, putative / beta-fructosidase, putative	0.69	1.01	0.89	1.23	-0.02	0.94	-0.10	1.05	0.47	1.82
cassava4.1_002971m.g	major CHO metabolism.degradation.sucrose.invertases.neutral	highly similar to (961) AT1G56560 | Symbols: | beta-fructofuranosidase, putative / invertase, putative / saccharase, putative / beta-fructosidase, putative	-0.41	-1.13	-0.10	-0.49	-0.03	-0.79	-0.01	-1.44	-0.50	-1.11
cassava4.1_003155m.g	major CHO metabolism.degradation.sucrose.invertases.neutral	highly similar to (903) AT5G22510 | Symbols: | beta-fructofuranosidase, putative / invertase, putative / saccharase, putative / beta-fructosidase, putative	-0.30	-0.71	0.03	-0.57	-0.05	-0.47	0.00	-1.52	-0.72	-1.61
cassava4.1_001840m.g	major CHO metabolism.degradation.sucrose.Susy	nearly identical (1488) AT4G02280 | Symbols: SUS3, ATSUS3 | SUS3 (sucrose synthase 3); UDP-glycosyltransferase/ sucrose synthase/ transferase, transferring glycosyl groups	0.96	1.95	0.15	0.45	0.22	0.77	1.60	3.03	0.72	1.89
cassava4.1_001867m.g	major CHO metabolism.degradation.sucrose.Susy	nearly identical (1395) AT3G43190 | Symbols: SUS4, ATSUS4 | SUS4; UDP-glycosyltransferase/ sucrose synthase/ transferase, transferring glycosyl groups	-0.81	-0.67	-0.86	-0.50	-0.37	-1.88	-0.67	-1.44	-0.81	-1.00
cassava4.1_001871m.g	major CHO metabolism.degradation.sucrose.Susy	nearly identical (1416) AT3G43190 | Symbols: SUS4, ATSUS4 | SUS4; UDP-glycosyltransferase/ sucrose synthase/ transferase, transferring glycosyl groups	-0.07	-0.19	0.38	0.58	-0.05	0.36	-1.10	0.50	0.96	1.16
cassava4.1_001874m.g	major CHO metabolism.degradation.sucrose.Susy	nearly identical (1167) AT1G73370 | Symbols: SUS6, ATSUS6 | SUS6 (SUCROSE SYNTHASE 6); UDP-glycosyltransferase/ sucrose synthase	2.07	3.16	0.75	0.93	0.48	1.67	1.90	2.75	0.58	2.21
cassava4.1_027790m.g	major CHO metabolism.degradation.sucrose.Susy	nearly identical (1178) AT1G73370 | Symbols: SUS6, ATSUS6 | SUS6 (SUCROSE SYNTHASE 6); UDP-glycosyltransferase/	0.80	2.04	0.47	1.06	-0.12	0.95	-0.50	-0.04	0.21	1.19

		sucrose synthase										
cassava4.1_005907m.g	major CHO metabolism.degradation.sucrose.hexokinase	highly similar to (627) AT1G50460 | Symbols: HKL1 | HKL1 (HEXOKINASE-LIKE 1); ATP binding / fructokinase/ glucokinase/ hexokinase	2.43	1.47	1.77	2.47	0.21	2.12	1.04	4.23	1.94	4.59
cassava4.1_005952m.g	major CHO metabolism.degradation.sucrose.hexokinase	highly similar to (627) AT1G50460 | Symbols: HKL1 | HKL1 (HEXOKINASE-LIKE 1); ATP binding / fructokinase/ glucokinase/ hexokinase	0.58	0.15	0.39	0.36	0.08	0.94	0.19	0.06	0.18	0.86
cassava4.1_006138m.g	major CHO metabolism.degradation.sucrose.hexokinase	highly similar to (754) AT4G29130 | Symbols: ATHXK1, GIN2, HXK1 | HXK1 (HEXOKINASE 1); ATP binding / fructokinase/ glucokinase/ hexokinase	0.77	1.27	0.28	0.59	0.21	0.62	0.29	1.94	0.46	1.14

Supplemental Table S7. Genes annotated to transcription factor and light signalling. The extent of differential expression is measured in terms of log2 fold change of treatment vs. control.

Cluster	Cassava ID	Mapman Category	Short Description	PG0	FG0	PG2	FG2	PG4	FG4	PG0	FG0	PG2	FG2
				120 day						45 day			
C1	cassava4.1_013886m.g	RNA.regulation of transcription.AP2/EREBP, APETALA2/Ethylene-responsive element binding protein family	Integrase-type DNA-binding superfamily protein	1.04	0.54	-0.39	-1.43	0.12	-0.22	-0.45	0.62	-0.22	0.69
	cassava4.1_013880m.g	RNA.regulation of transcription.AP2/EREBP, APETALA2/Ethylene-responsive element binding protein family	ethylene-responsive element binding protein	1.24	0.68	0.59	-0.05	0.16	0.37	0.45	1.92	0.78	2.08
	cassava4.1_010743m.g	RNA.regulation of transcription.bHLH,Basic Helix-Loop-Helix family	basic helix-loop-helix (bHLH) DNA-binding superfamily protein	1.33	1.46	0.21	0.20	0.16	0.15	0.78	1.45	1.01	1.80
	cassava4.1_009529m.g	RNA.regulation of transcription.AP2/EREBP, APETALA2/Ethylene-responsive element binding protein family	related to AP2 12	0.25	0.28	-0.43	-0.29	0.26	0.22	-0.20	1.09	0.37	1.16
	cassava4.1_011381m.g	RNA.regulation of transcription.SET-domain transcriptional regulator family	SET domain protein 20	-0.03	0.07	-0.55	0.38	0.12	-0.01	-1.28	1.00	0.43	1.69

	cassava4.1_014267m.g	RNA.regulation of transcription.AP2/EREBP, APETALA2/Ethylene-responsive element binding protein family	ethylene-responsive element binding protein	0.25	2.40	-1.99	-0.64	0.53	0.80	0.97	3.06	1.66	1.79
	cassava4.1_013174m.g	RNA.regulation of transcription.HSF,Heat-shock transcription factor family	heat shock factor 4	1.33	2.49	-0.19	0.94	0.26	2.99	2.04	3.38	1.09	3.09
	cassava4.1_009652m.g	RNA.regulation of transcription.AP2/EREBP, APETALA2/Ethylene-responsive element binding protein family	related to AP2 12	0.26	0.47	0.06	0.20	0.20	0.79	0.08	0.62	-0.28	0.39
	cassava4.1_034346m.g	RNA.regulation of transcription.GeBP like	DNA-binding storekeeper protein-related transcriptional regulator	0.45	0.77	-0.05	0.17	0.32	0.75	-0.38	1.04	-0.10	0.62
	cassava4.1_002532m.g	RNA.regulation of transcription.bHLH,Basic Helix-Loop-Helix family	Serine/threonine-protein kinase WNK (With No Lysine)-related	0.98	0.92	0.45	0.57	0.07	0.80	0.50	0.97	0.26	0.84
	cassava4.1_006085m.g	RNA.regulation of transcription.C2C2(Zn) CO-like, Constans-like zinc finger family	B-box type zinc finger protein with CCT domain	1.23	1.05	0.60	0.67	-0.06	0.88	0.15	2.05	-0.18	1.07
	cassava4.1_011035m.g	RNA.regulation of transcription.Aux/IAA family	phytochrome-associated protein 1	1.96	2.16	0.35	0.73	0.19	1.84	-0.48	4.07	0.76	2.43
	cassava4.1_002962m.g	RNA.regulation of transcription.SET-domain transcriptional regulator family	SU(VAR)3-9 homolog 1	0.15	0.28	-0.05	0.01	-0.12	0.13	0.06	0.83	0.02	0.47
	cassava4.1_013977m.g	RNA.regulation of transcription.bHLH,Basic Helix-Loop-Helix family	BIG PETAL P	-0.14	-0.11	-0.35	-0.13	0.09	1.43	-0.18	0.71	0.70	1.16
	cassava4.1_025964m.g	RNA.regulation of transcription.bHLH,Basic Helix-Loop-Helix family	basic helix-loop-helix (bHLH) DNA-binding superfamily protein	0.96	-0.36	-0.08	0.68	0.29	3.39	-0.54	1.55	0.80	3.09
	cassava4.1_012328m.g	RNA.regulation of transcription.G2-like transcription factor family, GARP	Homeodomain-like superfamily protein	-0.21	-0.09	0.02	0.47	-0.08	1.18	-0.07	0.52	-0.18	0.62
	cassava4.1_006932m.g	RNA.regulation of transcription.bHLH,Basic Helix-Loop-Helix family	NACL-inducible gene 1	0.63	-1.07	0.03	0.64	0.35	1.54	-1.76	0.69	-0.25	0.46
	cassava4.1_012221m.g	RNA.regulation of transcription.C2C2(Zn) DOF zinc finger family	Dof-type zinc finger DNA-binding family protein	0.97	0.51	1.56	1.20	0.36	1.48	-0.13	1.34	0.73	1.04
	cassava4.1_010727m.g	RNA.regulation of transcription.C2C2(Zn) GATA transcription factor family	Plant-specific GATA-type zinc finger transcription factor family protein	1.14	0.46	0.69	1.23	0.14	1.04	-0.53	0.96	0.10	1.04
	cassava4.1_018669m.g	RNA.regulation of transcription.C2C2(Zn) GATA transcription factor family	GATA transcription factor 15	0.61	0.57	0.56	0.80	0.32	0.97	-0.39	0.96	-0.25	0.96
	cassava4.1_027980m.g	RNA.regulation of transcription.C2C2(Zn) CO-like, Constans-like zinc finger family	CONSTANS-like 9	0.34	0.55	0.83	1.42	0.16	1.18	0.36	1.57	0.12	0.94

	cassava4.1_005340m.g	RNA.regulation of transcription.bHLH,Basic Helix-Loop-Helix family	basic helix-loop-helix (bHLH) DNA-binding superfamily protein	0.65	-0.19	1.38	1.40	-0.05	1.44	0.27	2.74	-0.19	0.97
	cassava4.1_016130m.g	RNA.regulation of transcription.MADS box transcription factor family	AGAMOUS-like 20	-0.19	-0.61	0.40	0.58	-0.06	0.62	-0.33	1.31	-0.19	0.78
	cassava4.1_008985m.g	RNA.regulation of transcription.MYB domain transcription factor family	myb domain protein 111	0.98	0.80	1.32	0.68	-0.78	1.73	0.32	2.04	1.28	2.27
	cassava4.1_008697m.g	RNA.regulation of transcription.ABI3/VP1-related B3-domain-containing transcription factor family	AP2/B3-like transcriptional factor family protein	0.78	0.82	0.65	1.07	0.09	1.51	0.73	2.37	0.72	2.13
	cassava4.1_013572m.g	RNA.regulation of transcription.Bromodomain proteins	DNA-binding bromodomain-containing protein	0.03	0.21	0.10	0.81	-0.26	0.63	0.03	1.23	0.05	1.05
	cassava4.1_022303m.g	RNA.regulation of transcription.bHLH,Basic Helix-Loop-Helix family	basic helix-loop-helix (bHLH) DNA-binding superfamily protein	1.45	1.47	1.87	2.15	0.36	2.42	0.80	3.42	1.96	3.91
	cassava4.1_002844m.g	RNA.regulation of transcription.bHLH,Basic Helix-Loop-Helix family	basic helix-loop-helix (bHLH) DNA-binding superfamily protein	0.65	1.68	1.38	3.24	0.39	3.03	0.13	3.37	0.96	5.32
	cassava4.1_019365m.g	RNA.regulation of transcription.Chromatin Remodeling Factors	unknow	0.71	1.08	0.52	1.33	0.20	1.64	0.05	2.30	0.70	2.59
	cassava4.1_028721m.g	RNA.regulation of transcription.MYB domain transcription factor family	myb domain protein 5	-0.24	-1.02	1.66	1.82	0.13	1.86	-1.03	4.27	1.09	5.32
	cassava4.1_010068m.g	RNA.regulation of transcription.AP2/EREBP, APETALA2/Ethylene-responsive element binding protein family	AP2/B3 transcription factor family protein	1.09	0.17	1.83	2.19	-0.07	1.74	-0.11	2.00	1.96	2.30
	cassava4.1_010829m.g	RNA.regulation of transcription.bHLH,Basic Helix-Loop-Helix family	basic helix-loop-helix (bHLH) DNA-binding superfamily protein	0.56	0.29	1.19	1.69	0.39	1.91	-0.56	1.27	0.81	2.96
	cassava4.1_016663m.g	RNA.regulation of transcription.bHLH,Basic Helix-Loop-Helix family	basic helix-loop-helix (bHLH) DNA-binding superfamily protein	0.13	-0.36	0.54	0.86	0.04	1.27	-0.21	0.68	0.42	1.40
	cassava4.1_034118m.g	RNA.regulation of transcription.C2C2(Zn) DOF zinc finger family	Dof-type zinc finger DNA-binding family protein	0.53	0.70	1.15	1.98	-0.09	1.79	-0.20	1.61	0.82	2.16
	cassava4.1_010892m.g	RNA.regulation of transcription.Aux/IAA family	phytochrome-associated protein 1	1.65	2.05	1.57	3.34	0.49	3.80	-0.35	3.15	1.06	3.80
	cassava4.1_012643m.g	RNA.regulation of transcription.MYB domain transcription factor family	myb domain protein 5	3.75	5.04	4.53	6.13	0.24	6.73	-1.99	6.15	2.00	7.93
	cassava4.1_004650m.g	RNA.regulation of transcription.GRAS transcription factor family	GRAS family transcription factor	1.88	1.13	2.42	3.08	0.75	1.79	-0.05	5.28	1.56	3.65
	cassava4.1_000786m.g	RNA.regulation of transcription.CCAAT box binding factor family, DR1	CCAAT-binding factor	0.19	0.68	0.26	0.61	-0.11	0.59	-0.86	1.75	0.23	1.28
	cassava4.1_014347m.g	RNA.regulation of transcription.HB,Homeobox transcription factor family	WUSCHEL related homeobox 13	0.29	0.60	0.19	0.35	-0.10	0.18	-0.36	1.30	0.54	1.01
	cassava4.1_012390m.g	RNA.regulation of transcription.AP2/EREBP,	ethylene-responsive element binding	1.26	1.35	0.87	1.50	0.17	0.20	0.02	1.80	0.82	1.56

		APETALA2/Ethylene-responsive element binding protein family	protein										
	cassava4.1_015101m.g	RNA.regulation of transcription.MYB domain transcription factor family	myb domain protein 5	5.68	4.89	2.96	4.06	0.50	3.91	-0.60	6.02	3.05	8.07
	cassava4.1_014074m.g	RNA.regulation of transcription.MYB domain transcription factor family	myb domain protein 4	4.71	5.65	4.06	6.04	0.52	5.36	0.22	8.73	4.14	8.64
	cassava4.1_016153m.g	RNA.regulation of transcription.MYB-related transcription factor family	Duplicated homeodomain-like superfamily protein	2.27	2.00	2.34	3.50	0.34	2.12	0.19	3.68	2.22	4.19
	cassava4.1_029459m.g	RNA.regulation of transcription.MYB domain transcription factor family	myb domain protein 3	-0.27	1.19	-0.13	1.21	-0.03	0.88	0.01	0.95	0.38	1.47
	cassava4.1_014632m.g	RNA.regulation of transcription.AP2/EREBP, APETALA2/Ethylene-responsive element binding protein family	related to AP2 6l	2.16	3.57	1.15	2.83	0.03	3.83	1.75	4.17	2.02	4.79
	cassava4.1_024069m.g	RNA.regulation of transcription.Chromatin Remodeling Factors	chromatin remodeling 31	1.61	1.88	0.53	1.95	-0.48	1.13	0.72	2.51	0.87	2.83
	cassava4.1_020908m.g	RNA.regulation of transcription.MYB domain transcription factor family	myb domain protein 5	0.63	2.04	0.83	2.64	0.12	1.39	0.34	3.58	0.87	6.10
	cassava4.1_011594m.g	RNA.regulation of transcription.MYB domain transcription factor family	myb domain protein 5	0.41	1.12	0.02	1.65	0.25	0.82	-0.02	1.62	-0.38	3.26
	cassava4.1_015395m.g	RNA.regulation of transcription.MYB domain transcription factor family	myb domain protein 4	1.44	2.43	1.28	3.10	-0.43	2.32	-0.73	2.46	2.08	6.80
	cassava4.1_016832m.g	RNA.regulation of transcription.AS2,Lateral Organ Boundaries Gene Family	LOB domain-containing protein 1	0.52	2.53	0.60	2.78	-0.02	2.25	-1.22	1.55	1.35	2.41
	cassava4.1_012667m.g	RNA.regulation of transcription.AS2,Lateral Organ Boundaries Gene Family	LOB domain-containing protein 41	1.05	3.60	3.24	5.84	0.72	5.36	-0.99	3.75	2.25	3.02
	cassava4.1_010104m.g	RNA.regulation of transcription.bHLH,Basic Helix-Loop-Helix family	basic helix-loop-helix (bHLH) DNA-binding superfamily protein	0.82	2.09	0.41	1.18	-0.36	1.59	0.30	1.19	0.27	1.62
	cassava4.1_000036m.g	RNA.regulation of transcription.Chromatin Remodeling Factors	transcription regulatory protein SNF2, putative	1.91	3.64	1.21	2.82	0.37	4.18	0.66	2.52	1.17	3.10
	cassava4.1_014014m.g	RNA.regulation of transcription.HB,Homeobox transcription factor family	homeobox protein 16	0.90	1.36	-0.04	1.24	-0.14	2.49	-0.40	1.50	0.33	1.69
	cassava4.1_005519m.g	RNA.regulation of transcription.C2H2 zinc finger family	C2H2-like zinc finger protein	0.45	1.33	0.13	0.94	0.12	1.37	-0.12	0.79	0.22	0.54
	cassava4.1_028193m.g	RNA.regulation of transcription.MYB domain transcription factor family	MYB-like 102	0.47	1.21	0.04	0.90	0.20	1.97	-0.01	1.24	0.02	0.31
	cassava4.1_012780m.g	RNA.regulation of transcription.bZIP transcription factor family	basic leucine zipper 9	1.16	1.75	0.59	1.18	-0.17	1.38	0.55	0.90	0.13	0.71
	cassava4.1_028489m.g	RNA.regulation of transcription.G2-like transcription factor family, GARP	Homeodomain-like superfamily protein	1.12	2.02	0.85	1.76	-0.08	2.05	0.09	1.07	-0.28	0.68
	cassava4.1_008256m.g	RNA.regulation of transcription.TCP transcription factor family	TCP family transcription factor	0.46	0.90	0.21	0.58	-0.05	0.72	-0.43	0.35	-0.04	0.14
	cassava4.1_012542m.g	RNA.regulation of transcription.MYB-related transcription factor family	Duplicated homeodomain-like	2.02	1.44	1.13	1.30	0.21	0.76	-0.48	0.95	0.66	1.86

			superfamily protein										
	cassava4.1_011111m.g	RNA.regulation of transcription.MYB domain transcription factor family	myb domain protein 73	1.99	1.73	1.91	2.88	0.12	0.83	-0.61	1.94	0.74	1.93
	cassava4.1_008733m.g	RNA.regulation of transcription.PHOR1	plant U-box 29	2.05	1.96	3.42	4.42	0.48	1.49	-0.54	3.22	2.59	3.29
	cassava4.1_014105m.g	RNA.regulation of transcription.C2C2(Zn) GATA transcription factor family	GATA transcription factor 1	-0.03	0.10	0.13	0.19	-0.03	0.01	-1.36	-0.28	-0.08	-0.09
	cassava4.1_033577m.g	RNA.regulation of transcription.C2H2 zinc finger family	zinc finger (C2H2 type) family protein	0.02	0.27	0.26	0.29	-0.12	-0.04	-1.20	0.09	-0.28	0.24
	cassava4.1_034350m.g	RNA.regulation of transcription.MYB domain transcription factor family	myb domain protein r1	1.39	1.46	1.73	1.87	0.37	0.13	-1.02	0.77	1.51	0.95
	cassava4.1_011082m.g	RNA.regulation of transcription.TCP transcription factor family	TCP family transcription factor 4	0.93	0.42	0.77	1.11	0.23	0.38	-1.04	-0.43	0.41	0.70
	cassava4.1_014713m.g	RNA.regulation of transcription.C2H2 zinc finger family	C2H2 and C2HC zinc fingers superfamily protein	1.15	1.82	2.69	3.45	0.38	1.40	1.99	3.23	1.28	0.89
	cassava4.1_003495m.g	RNA.regulation of transcription.GRAS transcription factor family	SCARECROW-like 8	0.65	0.01	0.43	0.12	0.03	0.57	0.60	1.44	-0.24	-0.18
	cassava4.1_019535m.g	RNA.regulation of transcription.General Transcription, TBP-binding protein	YEATS family protein	0.00	0.79	0.32	0.50	0.18	0.97	0.60	1.73	0.06	0.60
	cassava4.1_008339m.g	RNA.regulation of transcription.bZIP transcription factor family	G-box binding factor 3	0.99	0.99	0.17	0.27	0.05	1.45	1.71	3.83	0.30	1.14
	cassava4.1_014981m.g	RNA.regulation of transcription.HB,Homeobox transcription factor family	homeobox 7	1.32	1.89	0.61	1.78	0.38	3.33	2.84	5.06	0.73	2.33
	cassava4.1_007413m.g	RNA.regulation of transcription.C2H2 zinc finger family	C2H2 and C2HC zinc fingers superfamily protein	0.35	0.63	-0.03	-0.11	0.04	-0.03	0.85	1.57	0.31	0.71
	cassava4.1_009488m.g	RNA.regulation of transcription.C3H zinc finger family	CCCH-type zinc finger family protein	0.81	0.51	-0.39	-1.01	-0.05	0.10	1.34	2.25	-0.47	-0.25
	cassava4.1_009450m.g	RNA.regulation of transcription.C3H zinc finger family	CCCH-type zinc finger family protein	0.15	-0.84	-1.01	-2.10	-0.11	-0.04	1.06	1.40	-0.03	-0.51
	cassava4.1_012382m.g	RNA.regulation of transcription.CCAAT box binding factor family, HAP2	nuclear factor Y, subunit A9	-0.01	-0.68	-0.16	-0.96	0.05	0.84	2.53	3.26	-0.27	0.14
	cassava4.1_004896m.g	RNA.regulation of transcription.CPP(Zn),CPP1-related transcription factor family	TESMIN/TSO1-like CXC 2	0.38	-0.01	0.30	-0.25	-0.44	-0.19	1.69	1.24	0.43	0.31
	cassava4.1_009750m.g	RNA.regulation of transcription.HSF,Heat-shock transcription factor family	heat shock transcription factor A2	1.73	1.70	1.42	1.23	-0.11	-1.22	3.62	3.26	1.25	1.21
	cassava4.1_004528m.g	RNA.regulation of transcription.bHLH,Basic Helix-Loop-Helix family	basic helix-loop-helix (bHLH) DNA-binding superfamily protein	-0.31	-0.69	0.13	0.30	-0.05	0.21	0.44	1.01	0.17	0.27
	cassava4.1_014707m.g	RNA.regulation of transcription.C2H2 zinc finger family	zinc-finger protein 2	0.42	-0.04	1.20	0.28	0.48	0.19	0.80	2.20	1.10	1.15
	cassava4.1_014662m.g	RNA.regulation of transcription.C2H2 zinc finger family	salt tolerance zinc finger	0.67	-0.31	1.47	0.33	0.21	-0.08	0.25	2.08	0.82	0.84
	cassava4.1_017982m.g	RNA.regulation of transcription.C2H2 zinc finger family	C2H2-type zinc finger family protein	0.22	-1.16	1.78	1.60	0.30	0.56	0.65	2.86	1.10	0.24
	cassava4.1_014607m.g	RNA.regulation of transcription.C2H2 zinc finger family	salt tolerance zinc finger	0.68	-0.11	1.73	0.92	0.30	-0.06	0.21	2.00	1.19	-0.07

	cassava4.1_002960m.g	RNA.regulation of transcription.ARF, Auxin Response Factor family	auxin response factor 16	0.76	-0.59	0.20	-0.55	-0.11	-0.01	0.37	0.91	0.34	0.44
	cassava4.1_006005m.g	RNA.regulation of transcription.AP2/EREBP, APETALA2/Ethylene-responsive element binding protein family	related to AP2.7	0.90	-0.53	0.23	0.01	-0.11	0.09	0.66	1.52	0.15	1.18
	cassava4.1_007184m.g	RNA.regulation of transcription.bZIP transcription factor family	Basic-leucine zipper (bZIP) transcription factor family protein	0.24	-0.54	-0.11	-0.14	-0.22	-0.16	0.39	1.38	0.56	0.73
	cassava4.1_023048m.g	RNA.regulation of transcription.bHLH,Basic Helix-Loop-Helix family	basic helix-loop-helix (bHLH) DNA-binding superfamily protein	-0.01	-0.70	-0.09	-0.84	0.02	-0.19	0.14	1.31	0.00	-0.03
	cassava4.1_004069m.g	RNA.regulation of transcription.Trihelix, Triple-Helix transcription factor family	Duplicated homeodomain-like superfamily protein	-0.55	-2.66	-0.43	-2.80	-0.24	-0.77	0.46	1.65	0.22	0.01
C2	cassava4.1_015506m.g	RNA.regulation of transcription.bHLH,Basic Helix-Loop-Helix family	basic helix-loop-helix (bHLH) DNA-binding superfamily protein	-0.18	-0.21	-0.11	0.14	-0.31	-1.68	-0.42	-0.11	0.58	0.29
	cassava4.1_004906m.g	RNA.regulation of transcription.G2-like transcription factor family, GARP	RNA-binding (RRM/RBD/RNP motifs) family protein	0.50	0.81	0.38	0.43	-0.09	-0.75	0.69	0.36	0.88	1.39
	cassava4.1_033275m.g	RNA.regulation of transcription.LUG	LEUNIG_homolog	1.03	0.50	0.88	1.08	-0.49	-0.51	0.38	0.77	0.89	1.83
	cassava4.1_023141m.g	RNA.regulation of transcription.MYB domain transcription factor family	myb domain protein 73	1.55	1.27	1.62	2.34	0.39	1.15	0.92	1.34	1.96	2.53
	cassava4.1_000393m.g	RNA.regulation of transcription.Chromatin Remodeling Factors	SNF2 domain-containing protein / helicase domain-containing protein / zinc finger protein-related	0.21	0.50	0.32	0.87	-0.04	0.12	0.40	0.10	0.96	0.44
	cassava4.1_004902m.g	RNA.regulation of transcription.C2H2 zinc finger family	C2H2-like zinc finger protein	-0.03	0.52	0.15	0.77	-0.24	-0.42	-0.43	-1.48	0.47	0.41
	cassava4.1_006556m.g	RNA.regulation of transcription.G2-like transcription factor family, GARP	myb-like HTH transcriptional regulator family protein	0.44	0.76	0.87	1.24	0.08	-0.14	0.58	-0.50	0.56	0.62
	cassava4.1_000117m.g	RNA.regulation of transcription.Chromatin Remodeling Factors	chromatin remodeling 5	-0.01	0.52	0.22	0.39	-0.50	-0.77	0.79	-0.70	0.29	-0.04
	cassava4.1_000114m.g	RNA.regulation of transcription.Chromatin Remodeling Factors	chromatin remodeling 5	0.05	0.53	0.25	0.42	-0.26	-0.47	0.77	-0.91	0.20	0.08
	cassava4.1_000396m.g	RNA.regulation of transcription.C2H2 zinc finger family	zinc finger protein-related	-0.35	-0.37	0.06	0.08	-0.33	-0.89	0.24	-0.82	0.08	-0.37
	cassava4.1_000779m.g	RNA.regulation of transcription.Chromatin Remodeling Factors	DNA/RNA helicase protein	-0.55	-0.36	0.04	-0.47	-0.42	-1.49	0.43	-1.52	-0.01	-0.79
	cassava4.1_001653m.g	RNA.regulation of transcription.HB,Homeobox transcription factor family	Homeobox-leucine zipper family protein / lipid-binding START domain-containing protein	0.38	0.27	0.36	0.25	0.00	-0.17	0.86	-0.41	0.46	0.04

	cassava4.1_006759m.g	RNA.regulation of transcription.HB,Homeobox transcription factor family	BEL1-like homeodomain 6	0.14	0.68	0.07	0.64	-0.36	-0.73	0.46	-1.53	-0.11	-0.82
	cassava4.1_002618m.g	RNA.regulation of transcription.Nucleosome/chromatin assembly factor group	DDT domain-containing protein	-0.13	0.43	0.35	0.57	-0.21	-0.32	0.39	-1.09	0.23	-0.55
	cassava4.1_012115m.g	RNA.regulation of transcription.HSF,Heat-shock transcription factor family	heat shock transcription factor A6B	2.65	2.78	1.87	1.31	-1.25	0.57	2.54	0.90	0.83	1.02
	cassava4.1_002701m.g	RNA.regulation of transcription.Chromatin Remodeling Factors	SNF2 domain-containing protein / helicase domain-containing protein	0.55	1.18	-0.05	-0.11	-0.01	0.41	0.32	0.35	0.07	0.22
	cassava4.1_026051m.g	RNA.regulation of transcription.MADS box transcription factor family	AGAMOUS-like 19	0.39	0.89	-0.58	-0.62	-0.01	-0.06	0.30	0.08	0.03	-0.06
	cassava4.1_011766m.g	RNA.regulation of transcription.MYB domain transcription factor family	myb domain protein 31	0.93	1.77	-0.26	0.42	0.33	1.14	0.60	1.17	0.16	0.59
	cassava4.1_015207m.g	RNA.regulation of transcription.AP2/EREBP, APETALA2/Ethylene-responsive element binding protein family	Integrase-type DNA-binding superfamily protein	0.16	1.50	0.07	0.53	0.01	0.74	0.34	0.37	-0.14	0.04
	cassava4.1_005556m.g	RNA.regulation of transcription.C2H2 zinc finger family	C2H2-like zinc finger protein	0.73	0.94	0.52	0.65	0.23	0.60	0.55	0.38	0.18	0.54
	cassava4.1_011212m.g	RNA.regulation of transcription.HB,Homeobox transcription factor family	KNOTTED1-like homeobox gene 3	0.56	1.11	0.10	0.34	-0.09	0.20	0.28	0.14	0.03	0.20
	cassava4.1_008671m.g	RNA.regulation of transcription.bZIP transcription factor family	Basic-leucine zipper (bZIP) transcription factor family protein	0.36	1.00	0.02	0.10	-0.02	0.28	0.61	0.45	-0.06	-0.07
	cassava4.1_000870m.g	RNA.regulation of transcription.SET-domain transcriptional regulator family	SET domain protein 16	0.45	0.93	0.05	0.44	0.11	-0.09	0.50	0.42	0.06	-0.18
	cassava4.1_015051m.g	RNA.regulation of transcription.HB,Homeobox transcription factor family	homeobox 12	1.88	3.54	1.26	1.85	0.39	1.13	1.48	3.25	0.41	0.80
	cassava4.1_009563m.g	RNA.regulation of transcription.HB,Homeobox transcription factor family	homeobox from Arabidopsis thaliana	1.20	2.07	0.70	1.42	0.09	1.49	0.28	3.14	0.68	1.19
	cassava4.1_010339m.g	RNA.regulation of transcription.MYB domain transcription factor family	MYB-like 102	1.89	3.41	1.09	2.51	0.28	2.22	0.52	3.88	-0.22	0.54
	cassava4.1_005470m.g	RNA.regulation of transcription.C2H2 zinc finger family	C2H2 and C2HC zinc fingers superfamily protein	1.00	1.61	0.91	1.28	0.18	1.65	0.65	2.01	0.33	0.93
	cassava4.1_012335m.g	RNA.regulation of transcription.MYB domain transcription factor family	myb domain protein 62	2.38	4.45	1.50	3.31	-0.18	3.75	1.91	5.55	-0.27	2.18
	cassava4.1_010945m.g	RNA.regulation of transcription.MYB domain transcription factor family	myb domain protein 73	2.06	1.80	1.31	1.60	0.23	0.49	-0.14	1.97	0.36	0.41
	cassava4.1_014990m.g	RNA.regulation of transcription.C2C2(Zn) DOF zinc finger family	DOF zinc finger protein 1	1.45	2.99	0.63	1.59	-0.16	1.25	0.33	1.88	1.12	1.41
	cassava4.1_032831m.g	RNA.regulation of transcription.MYB domain transcription factor family	myb domain protein 6	0.51	2.05	0.35	1.22	-0.77	0.64	-0.48	1.27	-0.02	0.95
	cassava4.1_034188m.g	RNA.regulation of transcription.TCP transcription factor family	TCP family transcription factor	0.96	1.55	0.30	1.22	0.13	0.59	-0.22	1.49	0.42	1.17
	cassava4.1_019256m.g	RNA.regulation of transcription.bZIP transcription factor family	TGACG motif-binding factor 4	0.94	1.82	0.63	0.77	0.00	0.56	-0.39	0.23	0.42	0.46
	cassava4.1_004334m.g	RNA.regulation of transcription.Trihelix, Triple-Helix transcription factor	Protein of unknown function	0.62	1.42	0.07	0.54	-0.10	0.57	-0.18	0.36	0.22	0.38

		family	(DUF1421)										
	cassava4.1_000610m.g	RNA.regulation of transcription.ARF, Auxin Response Factor family	auxin response factor 19	0.36	1.03	0.10	0.19	-0.03	0.01	0.24	0.17	0.37	0.34
	cassava4.1_000578m.g	RNA.regulation of transcription.MYB domain transcription factor family	DIRP ;Myb-like DNA-binding domain	0.43	1.70	0.36	0.85	-0.43	-0.05	0.68	0.53	0.40	0.66
	cassava4.1_004089m.g	RNA.regulation of transcription.Trihelix, Triple-Helix transcription factor family	Duplicated homeodomain-like superfamily protein	1.07	2.83	-0.60	0.73	0.12	-0.79	-0.37	0.06	-0.29	0.41
	cassava4.1_004015m.g	RNA.regulation of transcription.bHLH,Basic Helix-Loop-Helix family	basic helix-loop-helix (bHLH) DNA-binding superfamily protein	2.66	3.26	0.61	1.06	0.14	1.05	0.37	1.60	1.03	2.29
	cassava4.1_006968m.g	RNA.regulation of transcription.GRAS transcription factor family	scarecrow-like 3	2.37	2.84	0.43	0.34	0.32	0.88	0.72	2.84	0.91	2.15
	cassava4.1_014027m.g	RNA.regulation of transcription.MYB-related transcription factor family	myb-like transcription factor family protein	1.23	1.69	0.12	0.35	0.38	0.74	-0.09	1.43	0.40	1.28
	cassava4.1_032811m.g	RNA.regulation of transcription.GRAS transcription factor family	GRAS family transcription factor	1.13	1.49	0.07	0.04	0.07	-0.36	0.42	0.79	0.21	0.93
	cassava4.1_006275m.g	RNA.regulation of transcription.Trihelix, Triple-Helix transcription factor family	Duplicated homeodomain-like superfamily protein	0.95	1.69	0.57	0.80	0.10	0.22	0.52	1.05	0.47	1.15
C3	cassava4.1_008554m.g	RNA.regulation of transcription.C2C2(Zn) CO-like, Constans-like zinc finger family	CONSTANS-like 9	-0.53	-0.50	-0.21	-0.14	-0.33	-0.28	0.91	0.11	0.52	0.40
	cassava4.1_000223m.g	RNA.regulation of transcription.Chromatin Remodeling Factors	chromatin remodeling factor CHD3 (PICKLE)	-0.12	0.19	0.17	0.19	-0.12	-0.23	0.93	-0.04	0.25	0.32
	cassava4.1_011448m.g	RNA.regulation of transcription.C2H2 zinc finger family	C2H2-type zinc finger family protein	-2.19	-1.42	-0.78	-2.07	-0.03	-2.63	0.02	-0.31	-0.90	-1.25
	cassava4.1_006018m.g	RNA.regulation of transcription.C2H2 zinc finger family	ZPR1 zinc-finger domain protein	-0.82	-0.71	-1.08	-1.42	-0.17	-0.32	0.12	0.18	-0.08	-0.23
	cassava4.1_015591m.g	RNA.regulation of transcription.bHLH,Basic Helix-Loop-Helix family	basic helix-loop-helix (bHLH) DNA-binding superfamily protein	-1.13	-2.37	-1.56	-2.53	-0.23	-2.24	0.30	0.14	1.79	-0.03
	cassava4.1_007445m.g	RNA.regulation of transcription.MYB domain transcription factor family	myb domain protein 55	-2.58	-3.51	-3.26	-2.87	-1.09	-2.66	-0.99	-2.40	-0.79	-1.87
	cassava4.1_002885m.g	RNA.regulation of transcription.ARF, Auxin Response Factor family	auxin response factor 9	-0.69	-1.65	-0.29	-1.56	-0.26	-0.79	0.32	-1.29	0.04	-0.64
	cassava4.1_011429m.g	RNA.regulation of transcription.MYB-related transcription factor family	myb-like transcription factor family protein	-0.98	-1.98	-0.87	-2.41	-0.08	-1.72	0.48	-1.18	-0.14	-0.95
	cassava4.1_034105m.g	RNA.regulation of transcription.PWWP domain protein	ataxia-telangiectasia mutated	-0.42	-0.58	-0.51	-1.02	-0.20	-0.53	0.21	-0.51	0.26	-0.28
	cassava4.1_014358m.g	RNA.regulation of transcription.C2H2 zinc finger family	zinc finger protein 4	-0.45	-1.76	-0.65	-1.47	0.06	-0.53	-0.29	-0.64	-0.44	-0.58
	cassava4.1_015213m.g	RNA.regulation of transcription.C2H2 zinc finger family	C2H2 and C2HC zinc fingers	-0.50	-2.98	-0.62	-2.28	0.05	-1.24	-0.31	-1.39	-0.57	-1.14

			superfamily protein										
	cassava4.1_004886m.g	RNA.regulation of transcription.bHLH,Basic Helix-Loop-Helix family	basic helix-loop-helix (bHLH) DNA-binding superfamily protein	-0.28	-0.92	-0.07	-0.34	0.16	-0.21	0.27	-0.18	0.15	-0.26
	cassava4.1_012303m.g	RNA.regulation of transcription.HSF,Heat-shock transcription factor family	heat shock transcription factor B2A	-0.26	-1.23	-0.43	-0.73	0.08	-0.50	0.32	-0.36	0.02	-0.43
	cassava4.1_014521m.g	RNA.regulation of transcription.Alfin-like	alfin-like 5	-0.25	-0.85	-0.29	-0.84	-0.13	-0.04	0.04	-0.04	-0.13	-0.21
	cassava4.1_009182m.g	RNA.regulation of transcription.SET-domain transcriptional regulator family	ASH1-related protein 2	-0.85	-2.95	-0.83	-2.24	0.22	-0.97	0.70	-0.13	-0.03	-0.18
	cassava4.1_002718m.g	RNA.regulation of transcription.GRAS transcription factor family	GRAS family transcription factor	0.07	-0.34	0.29	0.29	0.34	0.93	0.54	0.36	1.11	0.81
	cassava4.1_004194m.g	RNA.regulation of transcription.C2H2 zinc finger family	indeterminate(ID)-domain 5	0.41	-0.36	0.25	-0.29	0.23	-0.12	-0.11	0.08	1.26	1.54
	cassava4.1_013453m.g	RNA.regulation of transcription.bHLH,Basic Helix-Loop-Helix family	basic helix-loop-helix (bHLH) DNA-binding superfamily protein	-0.66	-2.11	0.06	-0.47	0.31	-0.08	-0.13	-0.43	1.37	1.49
	cassava4.1_016167m.g	RNA.regulation of transcription.AP2/EREBP, APETALA2/Ethylene-responsive element binding protein family	Integrase-type DNA-binding superfamily protein	-1.59	-3.12	-0.61	-2.97	-0.46	-2.00	-1.47	-0.57	0.65	0.52
	cassava4.1_009138m.g	RNA.regulation of transcription.MYB domain transcription factor family	myb domain protein 106	-1.56	-2.53	-0.55	-1.96	-0.09	-1.25	-1.02	-0.92	0.02	-0.51
	cassava4.1_026975m.g	RNA.regulation of transcription.ARR	response regulator 3	-3.37	-4.11	-0.21	-1.41	0.20	-0.01	-0.31	-0.61	-1.32	-1.85
	cassava4.1_029813m.g	RNA.regulation of transcription.AS2,Lateral Organ Boundaries Gene Family	Lateral organ boundaries (LOB) domain family protein	-1.85	-2.18	-1.67	-1.25	-0.33	-1.13	-0.61	-0.62	-1.10	-1.07
	cassava4.1_017720m.g	RNA.regulation of transcription.bZIP transcription factor family	Basic-leucine zipper (bZIP) transcription factor family protein	-0.50	-1.56	-0.23	-0.91	0.19	0.48	-0.16	0.07	-0.13	-0.73
	cassava4.1_011296m.g	RNA.regulation of transcription.C2C2(Zn) GATA transcription factor family	cytokinin-responsive gata factor 1	-0.30	-2.33	-0.46	-1.07	0.08	-0.06	-0.72	-0.32	-0.34	-0.73
	cassava4.1_013034m.g	RNA.regulation of transcription.HB,Homeobox transcription factor family	Homeobox-leucine zipper protein 4 (HB-4) / HD-ZIP protein	-1.37	-3.55	-1.26	-1.98	0.36	0.05	-1.21	-0.96	-0.52	-0.97
	cassava4.1_015797m.g	RNA.regulation of transcription.ARR	response regulator 5	-1.00	-5.80	-1.25	-1.93	-0.63	-0.40	-0.90	-1.43	-2.42	-3.46
	cassava4.1_018617m.g	RNA.regulation of transcription.C2C2(Zn) GATA transcription factor family	GATA transcription factor 15	-0.49	-1.81	0.26	-0.47	0.05	0.27	-0.47	-0.15	-0.52	-0.57
	cassava4.1_012449m.g	RNA.regulation of transcription.MYB-related transcription factor family	myb-like transcription factor family protein	-0.25	-2.26	0.42	0.24	0.08	0.57	-0.20	-0.41	-0.07	-0.69

	cassava4.1_030713m.g	RNA.regulation of transcription.C2C2(Zn) DOF zinc finger family	cycling DOF factor 2	-0.47	-1.65	-0.20	-0.18	0.10	1.15	0.12	-0.71	-0.32	-0.74
	cassava4.1_002464m.g	RNA.regulation of transcription.C3H zinc finger family	CCCH-type zinc finger protein with ARM repeat domain	-0.11	-0.95	-0.07	-0.40	-0.03	0.43	0.50	-0.56	-0.04	-0.27
	cassava4.1_012272m.g	RNA.regulation of transcription.MYB domain transcription factor family	myb domain protein 20	-1.57	-2.54	-0.55	-1.33	0.15	0.13	0.22	-1.35	-0.18	-1.36
	cassava4.1_016517m.g	RNA.regulation of transcription.bHLH,Basic Helix-Loop-Helix family	basic helix-loop-helix (bHLH) DNA-binding superfamily protein	-0.78	-2.75	-0.06	-1.22	0.12	-0.08	-0.58	-1.43	-0.46	-0.93
	cassava4.1_015258m.g	RNA.regulation of transcription.C2C2(Zn) CO-like, Constans-like zinc finger family	B-box zinc finger family protein	-0.48	-1.94	-0.41	-0.90	0.01	-0.34	-0.19	-1.01	-0.46	-0.99
	cassava4.1_006065m.g	RNA.regulation of transcription.C2C2(Zn) DOF zinc finger family	cycling DOF factor 2	-0.23	-1.34	-0.18	-0.59	0.13	0.23	0.01	-0.56	-0.18	-0.68
	cassava4.1_014772m.g	RNA.regulation of transcription.AS2,Lateral Organ Boundaries Gene Family	LOB domain-containing protein 37	-1.11	-4.49	-0.55	-0.46	0.01	0.42	-1.14	-2.56	-0.80	-1.52
	cassava4.1_002279m.g	RNA.regulation of transcription.HB,Homeobox transcription factor family	Homeobox-leucine zipper family protein / lipid-binding START domain-containing protein	-0.48	-1.55	-0.25	-0.51	0.12	0.47	-0.19	-1.10	-0.06	-0.48
	cassava4.1_006069m.g	RNA.regulation of transcription.AP2/EREBP, APETALA2/Ethylene-responsive element binding protein family	related to AP2.7	-0.44	-2.33	0.26	0.16	0.26	0.05	-0.31	-0.51	-0.21	-0.03
	cassava4.1_016873m.g	RNA.regulation of transcription.Aux/IAA family	AUX/IAA transcriptional regulator family protein	-1.07	-4.42	-0.77	-1.64	-0.36	-1.15	-1.14	-1.94	-1.09	-1.15
	cassava4.1_009290m.g	RNA.regulation of transcription.bHLH,Basic Helix-Loop-Helix family	cryptochrome-interacting basic-helix-loop-helix 1	-2.28	-6.15	-1.06	-1.58	-0.65	-1.25	-0.73	-3.07	-0.40	-1.82
	cassava4.1_014346m.g	RNA.regulation of transcription.C2C2(Zn) CO-like, Constans-like zinc finger family	B-box type zinc finger protein with CCT domain	-1.32	-3.91	-1.35	-0.79	-0.19	-0.10	-0.18	-1.60	-0.18	-1.16
	cassava4.1_031505m.g	RNA.regulation of transcription.TCP transcription factor family	TEOSINTE BRANCHED 1, cycloidea and PCF transcription factor 3	-1.16	-2.97	-1.12	-0.84	-0.29	-0.52	-0.85	-1.31	-0.19	-0.90
	cassava4.1_001154m.g	RNA.regulation of transcription.ARF, Auxin Response Factor family	auxin response factor 6	0.00	0.11	0.02	-0.29	-0.07	-0.84	-0.66	-0.25	-0.01	-0.43
	cassava4.1_025153m.g	RNA.regulation of transcription.C2H2 zinc finger family	C2H2-like zinc finger protein	-0.47	-0.50	-0.10	-0.71	-0.16	-1.06	-1.23	-1.50	-0.17	-1.42
	cassava4.1_005370m.g	RNA.regulation of transcription.C2H2 zinc finger family	indeterminate(ID)-domain 7	0.06	-0.01	-0.27	-0.60	-0.27	-0.84	-0.21	-0.93	-0.16	-0.40
	cassava4.1_030248m.g	RNA.regulation of transcription.AP2/EREBP,	AP2/B3 transcription factor family	0.35	0.67	-0.02	-0.19	-0.20	-1.86	1.32	-0.54	0.50	-0.31

		APETALA2/Ethylene-responsive element binding protein family	protein										
	cassava4.1_014911m.g	RNA.regulation of transcription.MYB domain transcription factor family	myb domain protein 48	-0.09	0.28	-0.63	-0.98	-0.17	-1.20	0.33	-0.98	-0.12	-0.82
	cassava4.1_011733m.g	RNA.regulation of transcription.C2C2(Zn) DOF zinc finger family	OBF binding protein 4	0.02	-0.64	-0.58	-1.73	-0.27	-2.01	-0.11	0.03	0.24	0.02
	cassava4.1_029328m.g	RNA.regulation of transcription.AP2/EREBP, APETALA2/Ethylene-responsive element binding protein family	cooperatively regulated by ethylene and jasmonate 1	0.64	0.14	0.01	-0.54	-0.09	-1.33	0.19	0.30	0.15	-0.09
	cassava4.1_004716m.g	RNA.regulation of transcription.ARF, Auxin Response Factor family	auxin response factor 10	0.17	-0.19	-0.22	-0.48	-0.10	-1.46	-0.19	0.02	-0.21	-0.83
	cassava4.1_013954m.g	RNA.regulation of transcription.HB,Homeobox transcription factor family	Homeobox-leucine zipper protein family	0.15	0.31	-0.67	-1.10	-0.62	-1.84	0.02	-0.25	-0.33	-1.15
	cassava4.1_001312m.g	RNA.regulation of transcription.Argonaute	Argonaute family protein	-0.03	-0.19	-0.04	-0.41	-0.06	-0.91	0.00	-0.30	-0.02	-0.45
	cassava4.1_005732m.g	RNA.regulation of transcription.SET-domain transcriptional regulator family	Rubisco methyltransferase family protein	0.09	0.01	-0.24	-1.08	-0.04	-1.62	-0.55	-0.57	-0.18	-0.78
	cassava4.1_013010m.g	RNA.regulation of transcription.MYB-related transcription factor family	Duplicated homeodomain-like superfamily protein	-0.23	-0.83	0.08	-0.19	0.12	-0.88	-0.61	-0.15	-0.50	-0.93
	cassava4.1_016343m.g	RNA.regulation of transcription.AP2/EREBP, APETALA2/Ethylene-responsive element binding protein family	Integrase-type DNA-binding superfamily protein	-0.45	-2.42	1.38	-0.45	0.43	-1.26	-2.63	-0.26	0.65	-1.69
	cassava4.1_003646m.g	RNA.regulation of transcription.bHLH,Basic Helix-Loop-Helix family	basic helix-loop-helix (bHLH) DNA-binding superfamily protein	-0.52	-1.44	-0.01	-0.57	-0.08	-1.24	-0.99	-0.86	0.15	-0.63
	cassava4.1_009755m.g	RNA.regulation of transcription.Aux/IAA family	indole-3-acetic acid inducible 9	-0.83	-1.91	-0.42	-1.24	-0.29	-0.88	-1.34	-1.69	-0.13	-1.23
	cassava4.1_014818m.g	RNA.regulation of transcription.HB,Homeobox transcription factor family	Homeobox-leucine zipper protein family	-1.09	-1.62	-0.30	-1.07	-0.14	-1.09	-1.31	-1.37	-0.20	-1.01
	cassava4.1_032473m.g	RNA.regulation of transcription.AP2/EREBP, APETALA2/Ethylene-responsive element binding protein family	ethylene responsive element binding factor 6	-1.31	-3.51	0.88	-2.74	0.21	-2.16	-2.02	-0.50	0.20	-3.31
	cassava4.1_013214m.g	RNA.regulation of transcription.AP2/EREBP, APETALA2/Ethylene-responsive element binding protein family	Integrase-type DNA-binding superfamily protein	-0.89	-3.46	1.03	-1.42	0.38	-1.65	-0.30	-0.07	0.90	-2.24
	cassava4.1_009548m.g	RNA.regulation of transcription.GRAS transcription factor family	GRAS family transcription factor	0.70	-1.97	2.36	1.30	0.91	-0.36	0.75	1.56	1.43	-0.04
	cassava4.1_004517m.g	RNA.regulation of transcription.TCP transcription factor family	TEOSINTE BRANCHED 1, cycloidea and PCF transcription factor 3	-0.13	-1.28	-0.32	-0.55	-0.16	-0.73	-0.48	-0.28	-0.16	-0.50
	cassava4.1_008459m.g	RNA.regulation of transcription.bZIP transcription factor family	G-box binding factor 3	-0.32	-1.18	-0.03	-0.48	-0.39	-0.40	0.30	0.16	-0.25	-0.81

	cassava4.1_005881m.g	RNA.regulation of transcription.bHLH,Basic Helix-Loop-Helix family	phytochrome interacting factor 3-like 1	-0.83	-2.29	-0.35	-1.32	-0.09	-0.62	0.29	-1.15	-0.74	-2.88
	cassava4.1_001542m.g	RNA.regulation of transcription.ARF, Auxin Response Factor family	auxin response factor 2	-0.14	-0.81	-0.25	-0.79	-0.10	-0.77	0.58	-0.39	-0.07	-1.55
	cassava4.1_007247m.g	RNA.regulation of transcription.E2F/DP transcription factor family	E2F transcription factor 1	-0.65	-1.20	-0.16	-1.04	-0.32	-0.89	0.81	-1.08	-0.41	-1.52
	cassava4.1_021075m.g	RNA.regulation of transcription.AP2/EREBP, APETALA2/Ethylene-responsive element binding protein family	unknow	0.12	-1.24	-0.63	-2.37	0.06	-2.48	1.50	-0.24	-0.07	-1.16
	cassava4.1_010627m.g	RNA.regulation of transcription.CCAAT box binding factor family, HAP2	nuclear factor Y, subunit A1	0.35	-0.27	-0.50	-1.60	0.09	-0.51	0.59	0.13	-0.48	-0.72
	cassava4.1_024442m.g	RNA.regulation of transcription.bHLH,Basic Helix-Loop-Helix family	basic helix-loop-helix (bHLH) DNA-binding superfamily protein	0.08	-1.96	-0.83	-1.58	-0.23	-1.18	0.31	-0.63	-0.94	-1.93
	cassava4.1_011611m.g	RNA.regulation of transcription.C2C2(Zn) DOF zinc finger family	OBF binding protein 4	-0.23	-1.06	-0.49	-1.33	-0.08	-0.49	-0.29	-0.35	-0.69	-1.18
	cassava4.1_010201m.g	RNA.regulation of transcription.HSF,Heat-shock transcription factor family	heat shock transcription factor C1	-0.07	-1.40	-0.31	-1.40	0.14	-1.10	0.02	0.24	-0.30	-0.95
	cassava4.1_015454m.g	RNA.regulation of transcription.MADS box transcription factor family	K-box region and MADS-box transcription factor family protein	0.26	0.31	0.36	0.06	-0.07	0.29	1.02	0.36	-0.24	-0.63
	cassava4.1_008877m.g	RNA.regulation of transcription.bHLH,Basic Helix-Loop-Helix family	basic helix-loop-helix (bHLH) DNA-binding superfamily protein	0.05	-0.16	0.02	-0.66	0.00	-0.86	1.07	-0.79	-0.19	-1.17
	cassava4.1_009601m.g	RNA.regulation of transcription.General Transcription	RNA polymerase III RPC4	-0.08	0.10	-0.33	-0.48	0.01	-0.52	0.56	-0.67	-0.48	-0.94
	cassava4.1_013975m.g	RNA.regulation of transcription.MADS box transcription factor family	K-box region and MADS-box transcription factor family protein	-0.05	0.08	-0.12	-0.34	-0.07	-0.49	1.20	-0.15	-0.13	-1.24
	cassava4.1_002453m.g	RNA.regulation of transcription.HB,Homeobox transcription factor family	homeodomain GLABROUS 2	-0.29	-0.03	-0.28	-0.26	0.04	-0.20	1.13	-0.33	0.18	-0.64
	cassava4.1_001456m.g	RNA.regulation of transcription.PHD finger transcription factor	Acyl-CoA N-acyltransferase with RING/FYVE/PHD-type zinc finger domain	-0.18	-0.16	-0.35	-0.52	-0.15	-0.34	0.86	-0.01	0.04	-0.42
	cassava4.1_015815m.g	RNA.regulation of transcription.ARR	response regulator 9	-1.09	-1.38	-0.64	-0.80	0.05	-1.60	-0.39	-1.56	-0.56	-2.69
	cassava4.1_017624m.g	RNA.regulation of transcription.Aux/IAA family	AUX/IAA transcriptional regulator family protein	-0.21	-1.00	-0.27	-0.65	-0.07	-1.19	0.06	-1.25	-0.94	-2.47
	cassava4.1_003856m.g	RNA.regulation of transcription.bHLH,Basic Helix-Loop-Helix family	basic helix-loop-helix (bHLH) DNA-binding superfamily protein	0.56	0.00	0.66	0.75	0.36	-0.55	-0.06	-0.81	0.11	-1.52
	cassava4.1_010425m.g	RNA.regulation of transcription.G2-like transcription factor family, GARP	Homeodomain-like superfamily protein	0.16	-0.57	0.20	-0.58	-0.09	-1.55	-0.25	-1.13	-0.46	-1.52

	cassava4.1_015098m.g	RNA.regulation of transcription.bHLH,Basic Helix-Loop-Helix family	basic helix-loop-helix (bHLH) DNA-binding superfamily protein	0.14	-0.19	-0.07	-0.27	-0.11	-0.49	-0.24	-0.97	-0.16	-0.90
	cassava4.1_022485m.g	RNA.regulation of transcription.Chromatin Remodeling Factors	SWITCH/sucrose nonfermenting 3C	-0.09	-0.08	-0.09	-0.14	-0.05	-0.19	-0.35	-1.47	-0.17	-0.83
	cassava4.1_000922m.g	RNA.regulation of transcription.NIN-like bZIP-related family	NIN like protein 7	0.09	0.24	-0.33	-0.08	0.04	-0.22	-0.03	-1.73	-0.06	-0.97
	cassava4.1_008682m.g	RNA.regulation of transcription.bHLH,Basic Helix-Loop-Helix family	basic helix-loop-helix (bHLH) DNA-binding superfamily protein	-0.37	-0.61	-0.30	-0.09	-0.20	-0.07	-0.09	-1.46	-0.48	-0.94
	cassava4.1_012977m.g	RNA.regulation of transcription.G2-like transcription factor family, GARP	phosphate starvation response 1	-0.52	-1.26	-0.32	-0.47	-0.05	-0.34	-0.60	-2.53	-0.28	-1.35
	cassava4.1_030151m.g	RNA.regulation of transcription.G2-like transcription factor family, GARP	Homeodomain-like superfamily protein	-0.45	-0.91	-0.39	-0.70	-0.32	-0.26	-0.61	-1.42	-0.30	-0.96
	cassava4.1_010526m.g	RNA.regulation of transcription.AP2/EREBP, APETALA2/Ethylene-responsive element binding protein family	related to AP2 4	-0.22	-0.45	-0.27	-0.41	0.04	0.32	-0.66	-2.31	-0.10	-1.48
	cassava4.1_001772m.g	RNA.regulation of transcription.HB,Homeobox transcription factor family	Homeobox-leucine zipper family protein / lipid-binding START domain-containing protein	-0.10	-0.45	-0.23	-0.27	-0.19	0.17	-0.11	-1.36	-0.12	-0.70
	cassava4.1_002969m.g	RNA.regulation of transcription.ARF, Auxin Response Factor family	auxin response factor 1	-0.26	-0.38	-0.10	-0.45	0.11	-0.20	-0.05	-0.90	-0.36	-0.59
	cassava4.1_004983m.g	RNA.regulation of transcription.C2H2 zinc finger family	indeterminate(ID)-domain 7	-0.17	-0.44	-0.08	-0.55	0.06	0.01	0.11	-0.93	-0.19	-0.83
	cassava4.1_014024m.g	RNA.regulation of transcription.CCAAT box binding factor family, HAP5	nuclear factor Y, subunit C2	-0.33	-0.94	-0.32	-0.76	0.00	-0.23	-0.13	-1.73	-0.38	-1.64
	cassava4.1_004443m.g	RNA.regulation of transcription.C2H2 zinc finger family	indeterminate(ID)-domain 5	0.22	-0.63	0.08	-0.62	0.04	-0.10	0.02	-1.49	0.21	-0.55
	cassava4.1_002166m.g	RNA.regulation of transcription.HB,Homeobox transcription factor family	homeodomain GLABROUS 2	-0.12	-0.41	-0.40	-0.48	-0.17	-0.28	-0.14	-0.90	-0.21	-0.66
	cassava4.1_023381m.g	RNA.regulation of transcription.Nucleosome/chromatin assembly factor group	HMG (high mobility group) box protein with ARID/BRIGHT DNA-binding domain	0.11	-0.40	-0.23	-0.57	0.02	-0.11	-0.28	-1.80	-0.03	-0.99
	cassava4.1_019292m.g	RNA.regulation of transcription.Aux/IAA family	unknow	-0.34	-1.61	-0.71	-0.73	-0.30	-0.88	-0.97	-2.47	-0.99	-2.15
	cassava4.1_005159m.g	RNA.regulation of transcription.bHLH,Basic Helix-Loop-Helix family	basic helix-loop-helix (bHLH) DNA-binding superfamily protein	-0.31	-1.77	0.09	-0.20	0.32	-1.21	-0.09	-2.45	0.03	-3.53
	cassava4.1_006801m.g	RNA.regulation of transcription.C2C2(Zn) DOF zinc finger family	cycling DOF factor 3	-0.50	-1.54	-0.46	-0.89	-0.31	-1.03	-0.89	-2.04	-0.52	-2.23
	cassava4.1_007759m.g	RNA.regulation of transcription.C2H2 zinc finger family	zinc finger protein 1	-0.44	-1.18	-0.13	-0.48	-0.01	-0.45	-0.19	-1.57	-0.39	-1.27
	cassava4.1_007787m.g	RNA.regulation of transcription.C2H2 zinc finger family	zinc finger protein 1	-0.38	-1.30	-0.26	-0.45	-0.12	-0.69	-0.09	-1.75	-0.28	-1.63

	cassava4.1_013797m.g	RNA.regulation of transcription.bZIP transcription factor family	Basic-leucine zipper (bZIP) transcription factor family protein	-0.42	-0.83	-0.21	-0.67	-0.03	-0.66	-0.28	-1.69	-0.41	-1.54
	cassava4.1_006206m.g	RNA.regulation of transcription.C2H2 zinc finger family	indeterminate(ID)-domain 5	-0.10	-0.77	0.27	-0.68	-0.01	-0.44	-0.11	-1.63	-0.13	-1.23
	cassava4.1_011554m.g	RNA.regulation of transcription.bHLH,Basic Helix-Loop-Helix family	basic helix-loop-helix (bHLH) DNA-binding superfamily protein	-0.36	-0.99	0.09	-0.17	0.20	0.09	-0.62	-1.32	-0.42	-1.54
	cassava4.1_009052m.g	RNA.regulation of transcription.G2-like transcription factor family, GARP	Homeodomain-like superfamily protein	-0.16	-1.60	0.30	-0.89	0.01	-0.34	-0.85	-2.04	-0.79	-2.26
	cassava4.1_007443m.g	RNA.regulation of transcription.PHOR1	ARM repeat superfamily protein	-0.73	-0.99	-0.15	-0.35	0.02	-0.47	-1.11	-2.06	-0.32	-2.11
	cassava4.1_002303m.g	RNA.regulation of transcription.GRAS transcription factor family	GRAS family transcription factor	-0.08	-0.45	-0.33	-1.01	-0.40	-0.21	0.29	-1.37	-0.41	-1.68
	cassava4.1_034237m.g	RNA.regulation of transcription.TCP transcription factor family	TEOSINTE BRANCHED 1, cycloidea, PCF (TCP)-domain family protein 20	-0.81	-0.77	-0.85	-0.74	-0.05	-0.48	-0.04	-1.93	-0.71	-2.47
	cassava4.1_004904m.g	RNA.regulation of transcription.General Transcription	tetratricopeptide repeat (TPR)-containing protein	0.18	0.47	-0.38	-0.74	0.39	-0.23	0.25	-0.68	-0.44	-1.38
	cassava4.1_008876m.g	RNA.regulation of transcription.C2H2 zinc finger family	C2H2-like zinc finger protein	0.17	-0.07	-0.22	-0.86	0.18	-0.45	-0.85	-1.19	-0.52	-1.73
	cassava4.1_013986m.g	RNA.regulation of transcription.HB,Homeobox transcription factor family	Homeobox-leucine zipper protein family	-0.44	0.03	-0.21	-0.57	0.08	-0.80	-0.48	-1.39	-0.59	-1.90
	cassava4.1_011931m.g	RNA.regulation of transcription.MYB-related transcription factor family	Homeodomain-like superfamily protein	-0.06	-0.15	-0.16	-0.79	-0.02	-0.50	-0.17	-1.25	-0.65	-1.45
	cassava4.1_010816m.g	RNA.regulation of transcription.TCP transcription factor family	plastid transcription factor 1	0.14	-0.19	-0.02	-0.31	0.17	0.00	-0.08	-0.69	-0.68	-0.99
	cassava4.1_011084m.g	RNA.regulation of transcription.G2-like transcription factor family, GARP	Homeodomain-like superfamily protein	-0.83	-1.26	-0.53	-0.44	0.58	-0.93	0.42	-1.81	-0.79	-1.31
	cassava4.1_009038m.g	RNA.regulation of transcription.G2-like transcription factor family, GARP	myb-like transcription factor family protein	-0.92	-1.20	0.26	0.51	-0.01	0.07	0.14	-2.11	-0.22	-1.18
	cassava4.1_013116m.g	RNA.regulation of transcription.MYB-related transcription factor family	Homeodomain-like/winged-helix DNA-binding family protein	-0.53	-0.64	0.17	0.02	0.06	-0.36	0.14	-1.03	-0.18	-0.72
	cassava4.1_012931m.g	RNA.regulation of transcription.MYB domain transcription factor family	myb domain protein 7	-1.03	-0.84	-0.12	-0.55	-0.30	-0.67	0.06	-1.37	-0.39	-1.51
	cassava4.1_006787m.g	RNA.regulation of transcription.Bromodomain proteins	bromodomain 4	0.04	-0.31	0.31	0.06	-0.07	-0.30	0.35	-1.42	-0.25	-1.50

	cassava4.1_031076m.g	RNA.regulation of transcription.SET-domain transcriptional regulator family	SET domain protein 14	-0.30	-0.50	0.05	0.04	-0.01	-0.27	0.47	-1.24	-0.16	-1.05
	cassava4.1_030224m.g	RNA.regulation of transcription.Trihelix, Triple-Helix transcription factor family	Duplicated homeodomain-like superfamily protein	-0.39	-0.04	-0.06	-0.39	0.19	-0.43	0.08	-1.08	-0.12	-0.54
	cassava4.1_031977m.g	RNA.regulation of transcription.C2C2(Zn) YABBY family	plant-specific transcription factor YABBY family protein	-0.69	-0.86	-0.04	-0.25	-0.10	-1.33	-0.06	-1.56	-0.41	-1.23
	cassava4.1_002216m.g	RNA.regulation of transcription.ARF, Auxin Response Factor family	auxin response factor 2	-0.50	-0.39	-0.26	-0.77	-0.24	-1.28	-0.21	-1.29	-0.22	-0.96
	cassava4.1_000076m.g	RNA.regulation of transcription.Chromatin Remodeling Factors	DNA binding;ATP binding;nucleic acid binding;binding;helicases;ATP binding;DNA binding;helicases	0.05	0.16	0.18	0.07	-0.06	-0.57	0.33	-1.08	0.13	-0.29
	cassava4.1_025037m.g	RNA.regulation of transcription.Trihelix, Triple-Helix transcription factor family	Duplicated homeodomain-like superfamily protein	-0.30	-0.19	0.06	-0.33	-0.18	-0.93	-0.03	-1.16	-0.26	-0.68
	cassava4.1_007837m.g	RNA.regulation of transcription.bZIP transcription factor family	bZIP transcription factor family protein	-0.24	-1.30	-0.23	-0.31	-0.33	-0.97	-0.08	-2.39	-0.36	-1.23
	cassava4.1_000316m.g	RNA.regulation of transcription.Chromatin Remodeling Factors	SNF2 domain-containing protein / helicase domain-containing protein / F-box family protein	-0.18	-0.72	0.29	0.12	0.19	-0.41	0.24	-1.41	0.11	-0.41
	cassava4.1_007149m.g	RNA.regulation of transcription.G2-like transcription factor family, GARP	Homeodomain-like superfamily protein	-0.41	-0.70	-0.16	-0.48	-0.18	-0.70	-0.03	-1.42	-0.08	-0.89
	cassava4.1_023603m.g	RNA.regulation of transcription.HB,Homeobox transcription factor family	BEL1-like homeodomain 2	-0.16	-0.63	0.03	-0.32	-0.01	-0.49	0.12	-1.29	-0.08	-0.66
	cassava4.1_000754m.g	RNA.regulation of transcription.C3H zinc finger family	zinc knuckle (CCHC-type) family protein	-0.48	-0.88	-0.30	-0.54	-0.22	-1.17	0.33	-1.70	-0.15	-1.33
	cassava4.1_033475m.g	RNA.regulation of transcription.MADS box transcription factor family	unclear	-0.53	-1.41	-0.32	-1.01	-0.37	-2.18	0.03	-3.26	-0.12	-2.34
	cassava4.1_001790m.g	RNA.regulation of transcription.HB,Homeobox transcription factor family	BEL1-like homeodomain 2	0.05	-0.14	-0.06	-0.10	0.01	-0.25	0.20	-1.11	-0.13	-0.34
	cassava4.1_001576m.g	RNA.regulation of transcription.ABI3/VP1-related B3-domain-containing transcription factor family	high-level expression of sugar-inducible gene 2	0.11	0.06	0.19	0.40	0.09	-0.27	0.37	-1.16	0.13	-0.57
	cassava4.1_000609m.g	RNA.regulation of transcription.ARF, Auxin Response Factor family	auxin response factor 19	-0.99	-1.31	-0.39	-0.59	-0.35	-0.97	-0.20	-3.01	-0.27	-1.50
	cassava4.1_003781m.g	RNA.regulation of transcription.Trihelix, Triple-Helix transcription factor family	Duplicated homeodomain-like superfamily protein	-0.48	-0.32	-0.04	-0.10	-0.04	-0.62	0.06	-1.55	0.03	-0.85

	cassava4.1_011813m.g	RNA.regulation of transcription.bZIP transcription factor family	bZIP transcription factor family protein	-0.35	-0.46	-0.41	-1.96	0.24	-0.99	-0.14	-1.83	-0.46	-1.23
	cassava4.1_011886m.g	RNA.regulation of transcription.C2C2(Zn) GATA transcription factor family	GATA transcription factor 5	-0.20	-0.81	-0.41	-1.29	-0.04	-1.50	-0.58	-1.84	-0.62	-1.35
	cassava4.1_013480m.g	RNA.regulation of transcription.HB,Homeobox transcription factor family	homeobox 1	-0.15	-2.57	-0.64	-2.87	0.18	-2.42	-0.89	-3.14	-0.12	-1.41
	cassava4.1_013746m.g	RNA.regulation of transcription.MYB-related transcription factor family	myb-like transcription factor family protein	-0.34	-2.43	-1.31	-3.13	-0.08	-3.04	-1.15	-3.96	-0.20	-2.33
	cassava4.1_024212m.g	RNA.regulation of transcription.TCP transcription factor family	TEOSINTE BRANCHED, cycloidea and PCF (TCP) 14	0.22	-0.24	-0.86	-1.60	-0.23	-0.80	-0.57	-1.07	-0.59	-1.21
	cassava4.1_017926m.g	RNA.regulation of transcription.Nucleosome/chromatin assembly factor group	high mobility group B3	0.06	-0.77	-0.54	-1.18	-0.02	-1.04	-0.45	-0.55	-0.73	-1.90
	cassava4.1_013973m.g	RNA.regulation of transcription.MYB-related transcription factor family	myb-like transcription factor family protein	0.22	-0.33	-0.13	-0.72	-0.02	-0.37	-0.31	-0.34	-0.55	-1.28
	cassava4.1_018525m.g	RNA.regulation of transcription.Nucleosome/chromatin assembly factor group	high mobility group B3	0.06	-0.38	-0.41	-0.76	0.02	-0.46	-0.33	-0.24	-0.82	-1.68
	cassava4.1_008584m.g	RNA.regulation of transcription.General Transcription	Transcription factor IIA, alpha/beta subunit	-0.07	-0.43	-0.40	-0.65	-0.01	-0.67	-0.07	-0.53	-0.28	-1.11
	cassava4.1_012505m.g	RNA.regulation of transcription.MYB domain transcription factor family	myb domain protein 31	-0.12	-1.01	-0.13	-1.27	0.23	-1.24	-0.33	-0.97	-0.58	-1.86
	cassava4.1_014431m.g	RNA.regulation of transcription.Aux/IAA family	indole-3-acetic acid inducible 11	0.11	-0.47	-0.70	-1.74	-0.29	-1.21	-0.21	-1.20	-0.79	-1.89
	cassava4.1_033803m.g	RNA.regulation of transcription.AP2/EREBP, APETALA2/Ethylene-responsive element binding protein family	cooperatively regulated by ethylene and jasmonate 1	-0.08	-0.13	-0.26	-0.92	0.08	-0.95	0.17	-0.77	-0.31	-1.40
	cassava4.1_012485m.g	RNA.regulation of transcription.TCP transcription factor family	TEOSINTE BRANCHED 1, cycloidea, PCF (TCP)-domain family protein 20	-0.11	-0.34	-0.24	-1.12	-0.20	-0.97	-0.19	-0.98	-0.28	-1.27
	cassava4.1_015642m.g	RNA.regulation of transcription.bHLH,Basic Helix-Loop-Helix family	basic helix-loop-helix (bHLH) DNA-binding superfamily protein	-0.92	-2.17	-1.58	-1.87	-0.16	-1.61	-1.12	-1.17	0.31	-1.34
	cassava4.1_006867m.g	RNA.regulation of transcription.bHLH,Basic Helix-Loop-Helix family	basic helix-loop-helix (bHLH) DNA-binding superfamily protein	-0.51	-1.06	-0.48	-0.91	0.19	-0.66	-0.62	-0.51	-0.15	-0.73
	cassava4.1_008593m.g	RNA.regulation of transcription.C2C2(Zn) CO-like, Constans-like zinc	chloroplast import apparatus 2	-0.62	-0.88	-0.77	-1.64	-0.08	-1.37	-0.79	-0.60	0.01	-0.77

		finger family											
	cassava4.1_013177m.g	RNA.regulation of transcription.HB,Homeobox transcription factor family	homeobox protein 2	-0.61	-1.53	-0.29	-1.93	0.04	-1.28	-0.54	-0.64	0.11	-1.03
	cassava4.1_001995m.g	RNA.regulation of transcription.ARF, Auxin Response Factor family	auxin response factor 4	-0.21	-0.65	0.17	-0.97	0.21	-1.49	-0.50	-1.29	0.44	-0.45
	cassava4.1_012160m.g	RNA.regulation of transcription.G2-like transcription factor family, GARP	Homeodomain-like superfamily protein	-0.68	-1.44	-0.39	-1.50	0.06	-1.47	-0.83	-1.35	0.03	-0.48
	cassava4.1_016852m.g	RNA.regulation of transcription.C2H2 zinc finger family	zinc finger protein 7	-0.74	-1.87	-0.54	-2.09	0.05	-2.80	-0.81	-1.08	-0.38	-1.61
	cassava4.1_032952m.g	RNA.regulation of transcription.bZIP transcription factor family	TraB family protein	-0.83	-1.60	-0.23	-1.02	0.02	-2.72	-0.25	-1.86	-0.15	-1.63
	cassava4.1_002286m.g	RNA.regulation of transcription.HSF,Heat-shock transcription factor family	Nucleic acid-binding, OB-fold-like protein	-0.61	-0.97	-0.12	-1.12	-0.05	-1.71	-0.33	-1.08	-0.22	-0.76
	cassava4.1_014928m.g	RNA.regulation of transcription.Aux/IAA family	indole-3-acetic acid inducible 14	-0.29	-1.29	-0.49	-1.09	-0.30	-1.28	-1.02	-1.13	-0.68	-1.48
	cassava4.1_028782m.g	RNA.regulation of transcription.AP2/EREBP, APETALA2/Ethylene-responsive element binding protein family	Integrase-type DNA-binding superfamily protein	-0.27	-2.16	-0.92	-1.97	-0.01	-0.60	-1.01	-1.84	-0.64	-2.53
	cassava4.1_014217m.g	RNA.regulation of transcription.C2C2(Zn) DOF zinc finger family	Protein of unknown function (DUF688)	-0.01	-1.88	-0.02	-1.42	0.20	-0.63	-0.67	-1.18	-0.42	-1.34
	cassava4.1_003741m.g	RNA.regulation of transcription.GRAS transcription factor family	RGA-like 2	-0.47	-1.75	-0.53	-1.19	-0.12	-0.90	-0.73	-1.27	-0.62	-0.96
	cassava4.1_028288m.g	RNA.regulation of transcription.C2C2(Zn) CO-like, Constans-like zinc finger family	B-box type zinc finger protein with CCT domain	-1.81	-5.01	0.08	-2.08	-0.21	-3.08	0.00	-2.48	-0.42	-2.39
	cassava4.1_025708m.g	RNA.regulation of transcription.bHLH,Basic Helix-Loop-Helix family	phytochrome interacting factor 3-like 5	-0.57	-1.96	-0.32	-0.63	-0.10	-1.44	0.02	-1.25	-0.41	-1.26
	cassava4.1_010785m.g	RNA.regulation of transcription.C2H2 zinc finger family	C2H2-type zinc finger family protein	-1.38	-3.58	-0.94	-1.36	-0.67	-2.99	-1.19	-2.19	-1.19	-2.26
	cassava4.1_028748m.g	RNA.regulation of transcription.HSF,Heat-shock transcription factor family	heat shock transcription factor A3	-0.38	-1.50	0.03	-0.45	0.10	-0.99	-0.19	-0.71	-0.30	-1.35
	cassava4.1_015068m.g	RNA.regulation of transcription.C2C2(Zn) CO-like, Constans-like zinc finger family	salt tolerance homologue	-0.77	-2.59	-0.32	-1.45	0.09	-1.01	0.08	-1.11	-0.62	-1.65
	cassava4.1_010378m.g	RNA.regulation of transcription.C2C2(Zn) CO-like, Constans-like zinc finger family	CONSTANS-like 5	-0.21	-1.53	-0.24	-1.26	-0.10	-0.91	0.02	-0.95	-0.38	-1.00
	cassava4.1_011192m.g	RNA.regulation of transcription.C2C2(Zn) CO-like, Constans-like zinc finger family	CONSTANS-like 4	0.00	-0.88	-0.25	-0.84	0.11	-0.34	-0.01	-0.51	-0.39	-0.99
	cassava4.1_013873m.g	RNA.regulation of transcription.C2C2(Zn) CO-like, Constans-like zinc finger family	B-box type zinc finger family protein	-0.52	-2.47	-0.20	-1.85	0.02	-1.30	-0.45	-1.45	-0.94	-2.55

	cassava4.1_011725m.g	RNA.regulation of transcription.C2C2(Zn) CO-like, Constans-like zinc finger family	CONSTANS-like 4	0.10	-1.16	-0.20	-1.16	0.10	-0.78	0.07	-0.98	-0.49	-1.40
	cassava4.1_010032m.g	RNA.regulation of transcription.C3H zinc finger family	CCCH-type zinc finger family protein	-0.37	-2.42	-0.01	-2.45	0.04	-1.45	0.21	-1.88	-0.61	-2.66
	cassava4.1_014900m.g	RNA.regulation of transcription.Aux/IAA family	indole-3-acetic acid 7	-0.91	-2.87	-1.64	-2.60	-0.59	-2.93	-0.96	-2.40	-1.16	-2.32
	cassava4.1_034451m.g	RNA.regulation of transcription.C2H2 zinc finger family	zinc finger (C2H2 type) family protein	-0.93	-2.03	-1.16	-2.23	-0.25	-1.95	-1.12	-1.65	-1.03	-2.04
	cassava4.1_013130m.g	RNA.regulation of transcription.C2C2(Zn) GATA transcription factor family	ZIM-like 1	-0.45	-1.74	-0.50	-1.19	0.00	-1.20	0.11	-1.70	-0.42	-1.75
	cassava4.1_016451m.g	RNA.regulation of transcription.C2C2(Zn) YABBY family	Plant-specific transcription factor YABBY family protein	-0.63	-1.73	-0.57	-1.48	-0.09	-1.03	0.09	-1.53	-0.65	-2.16
	cassava4.1_031586m.g	RNA.regulation of transcription.C2H2 zinc finger family	C2H2-type zinc finger family protein	-0.48	-2.11	-1.14	-1.60	-0.25	-1.75	-0.10	-1.51	-0.88	-2.12
	cassava4.1_015577m.g	RNA.regulation of transcription.Aux/IAA family	indole-3-acetic acid inducible 14	-0.85	-2.00	-0.53	-1.85	-0.15	-2.10	-0.18	-1.97	-0.77	-2.63
	cassava4.1_027196m.g	RNA.regulation of transcription.MYB domain transcription factor family	myb domain protein 60	-0.92	-2.02	-0.75	-1.44	-0.13	-1.80	-0.43	-1.31	-0.53	-2.21
	cassava4.1_013503m.g	RNA.regulation of transcription.MYB-related transcription factor family	Duplicated homeodomain-like superfamily protein	-0.19	-0.71	-0.33	-0.85	-0.06	-1.06	0.08	-0.60	-0.29	-0.97
	cassava4.1_013487m.g	RNA.regulation of transcription.bHLH,Basic Helix-Loop-Helix family	basic helix-loop-helix (bHLH) DNA-binding superfamily protein	-0.94	-1.24	-0.23	-1.14	-0.04	-0.88	-0.33	-0.95	-0.10	-0.95
	cassava4.1_007846m.g	RNA.regulation of transcription.MYB domain transcription factor family	myb domain protein 61	-1.82	-2.61	-0.91	-3.13	-0.08	-2.22	-1.43	-2.74	-0.71	-2.38
	cassava4.1_005852m.g	RNA.regulation of transcription.AP2/EREBP, APETALA2/Ethylene-responsive element binding protein family	Integrase-type DNA-binding superfamily protein	-0.92	-1.59	-0.55	-1.11	0.10	-1.60	-0.22	-2.35	0.06	-1.20
	cassava4.1_010095m.g	RNA.regulation of transcription.Aux/IAA family	indole-3-acetic acid inducible 9	-1.00	-1.45	-0.74	-1.34	-0.21	-1.75	-0.47	-1.66	-0.36	-0.98
	cassava4.1_034383m.g	RNA.regulation of transcription.Bromodomain proteins	DNA-binding bromodomain-containing protein	-0.76	-2.23	-0.22	-2.33	-0.72	-3.36	0.56	-3.12	-0.25	-2.62
	cassava4.1_028769m.g	RNA.regulation of transcription.FHA transcription factor	Forkhead-associated (FHA) domain-containing protein	-0.47	-1.16	-0.33	-1.36	-0.21	-1.33	0.00	-1.59	-0.22	-0.95
	cassava4.1_002408m.g	RNA.regulation of transcription.ARF, Auxin Response Factor family	auxin response factor 9	-0.38	-1.17	-0.36	-1.14	-0.19	-0.91	-0.01	-1.53	0.17	-0.80
	cassava4.1_001928m.g	RNA.regulation of transcription.HB,Homeobox transcription factor family	homeobox-7	-0.39	-1.21	-0.18	-1.13	-0.20	-0.99	-0.05	-1.33	0.01	-0.77
	cassava4.1_027046m.g	RNA.regulation of transcription.bHLH,Basic Helix-Loop-Helix family	basic helix-loop-helix (bHLH) DNA-binding superfamily protein	-1.47	-4.61	-0.78	-2.63	-0.04	-3.48	-0.24	-4.02	-0.10	-2.56

	cassava4.1_025106m.g	RNA.regulation of transcription.C3H zinc finger family	zinc knuckle (CCHC-type) family protein	-0.91	-3.15	-0.35	-2.06	-0.16	-2.83	-0.59	-2.43	-0.40	-1.82
	cassava4.1_028677m.g	RNA.regulation of transcription.GRAS transcription factor family	GRAS family transcription factor family protein	-0.92	-4.06	-0.68	-2.72	-0.14	-3.84	0.17	-3.18	-0.26	-2.63
	cassava4.1_025707m.g	RNA.regulation of transcription.MADS box transcription factor family	AGAMOUS-like 42	-1.20	-3.68	-0.26	-2.54	-0.35	-3.68	-0.01	-2.70	-0.33	-2.92
	cassava4.1_031585m.g	RNA.regulation of transcription.C2C2(Zn) GATA transcription factor family	cytokinin-responsive gata factor 1	-1.15	-5.84	-0.60	-4.98	-0.05	-5.30	-0.80	-4.06	-0.70	-4.53
	cassava4.1_007384m.g	RNA.regulation of transcription.G2-like transcription factor family, GARP	GBF's pro-rich region-interacting factor 1	-0.26	-2.08	-0.20	-1.61	0.03	-1.65	0.03	-1.60	-0.30	-1.27
	cassava4.1_020375m.g	RNA.regulation of transcription.MYB-related transcription factor family	RAD-like 1	-1.79	-6.20	-1.81	-5.94	-0.28	-6.22	-0.16	-5.13	-0.27	-4.93
	cassava4.1_000118m.g	RNA.regulation of transcription.HB,Homeobox transcription factor family	homeobox-1	-0.72	-2.63	-0.45	-2.33	-0.11	-2.89	-0.33	-2.62	-0.63	-2.43
	cassava4.1_007418m.g	RNA.regulation of transcription.bHLH,Basic Helix-Loop-Helix family	basic helix-loop-helix (bHLH) DNA-binding superfamily protein	-0.89	-2.52	-0.66	-2.38	-0.10	-2.56	-0.15	-3.00	-0.46	-2.65
	cassava4.1_022856m.g	RNA.regulation of transcription.MYB domain transcription factor family	myb-like HTH transcriptional regulator family protein	-1.15	-5.04	0.01	-3.57	0.06	-4.15	0.11	-4.96	-0.52	-4.66
	cassava4.1_012587m.g	RNA.regulation of transcription.C2C2(Zn) GATA transcription factor family	cytokinin-responsive gata factor 1	-0.59	-2.05	-0.26	-1.70	0.02	-1.43	-0.37	-1.74	-0.40	-1.55
	cassava4.1_012217m.g	RNA.regulation of transcription.MYB-related transcription factor family	Homeodomain-like superfamily protein	-0.64	-2.31	-0.32	-2.14	-0.03	-1.85	-0.18	-2.51	-0.43	-1.91
	cassava4.1_001979m.g	RNA.regulation of transcription.ARF, Auxin Response Factor family	auxin response factor 4	-0.98	-1.81	-0.77	-1.86	-0.20	-1.37	-0.91	-1.85	-0.79	-1.87
	cassava4.1_012613m.g	RNA.regulation of transcription.bHLH,Basic Helix-Loop-Helix family	basic helix-loop-helix (bHLH) DNA-binding superfamily protein	-1.02	-3.46	-0.75	-3.24	0.31	-1.78	-0.92	-3.73	-1.12	-3.56
	cassava4.1_017143m.g	RNA.regulation of transcription.C2C2(Zn) YABBY family	plant-specific transcription factor YABBY family protein	-0.99	-1.71	-0.67	-1.18	-0.06	-1.08	-0.56	-1.73	-0.60	-1.41
	cassava4.1_004019m.g	RNA.regulation of transcription.B3 transcription factor family	AP2/B3-like transcriptional factor family protein	-0.38	-0.96	0.04	-0.43	0.16	-0.44	0.30	-0.64	-0.01	-0.79
	cassava4.1_017177m.g	RNA.regulation of transcription.C2C2(Zn) YABBY family	plant-specific transcription factor YABBY family protein	-0.80	-1.78	-0.29	-1.04	-0.20	-1.03	-0.27	-1.42	-0.41	-1.65

	cassava4.1_010237m.g	RNA.regulation of transcription.C2H2 zinc finger family	C2H2-like zinc finger protein	-1.30	-3.05	-0.87	-1.92	0.19	-1.85	-0.03	-2.16	-0.54	-2.53
	cassava4.1_011476m.g	RNA.regulation of transcription.MYB-related transcription factor family	Homeodomain-like superfamily protein	-0.59	-2.06	-0.32	-0.89	-0.11	-1.24	-0.18	-1.77	-0.35	-1.65
	cassava4.1_007456m.g	RNA.regulation of transcription.bZIP transcription factor family	bZIP transcription factor family protein	-1.61	-2.68	-0.62	-2.51	0.05	-1.60	0.25	-3.64	0.06	-2.80
	cassava4.1_017602m.g	RNA.regulation of transcription.C2C2(Zn) CO-like, Constans-like zinc finger family	B-box type zinc finger family protein	-0.64	-2.79	-0.65	-1.82	-0.12	-1.15	0.04	-2.09	-0.41	-1.69
	cassava4.1_031269m.g	RNA.regulation of transcription.C2H2 zinc finger family	C2H2-type zinc finger family protein	-1.81	-6.11	-1.50	-3.90	-0.51	-3.28	-0.63	-4.55	-0.70	-4.55
	cassava4.1_020357m.g	RNA.regulation of transcription.MYB-related transcription factor family	RAD-like 6	-1.70	-3.91	-1.17	-2.80	-0.11	-2.10	-0.03	-2.91	-0.51	-2.43
	cassava4.1_027289m.g	RNA.regulation of transcription.NIN-like bZIP-related family	Plant regulator RWP-RK family protein	-0.84	-2.07	-0.56	-1.66	-0.10	-1.49	0.17	-2.09	-0.11	-1.56
	cassava4.1_030452m.g	RNA.regulation of transcription.C2C2(Zn) DOF zinc finger family	cycling DOF factor 2	-1.54	-3.97	-1.72	-2.83	-0.41	-4.02	-1.36	-5.65	-0.77	-4.45
	cassava4.1_006216m.g	RNA.regulation of transcription.MYB-related transcription factor family	Homeodomain-like superfamily protein	-1.05	-2.42	-1.03	-2.27	-0.51	-2.89	-0.23	-3.55	-0.46	-2.82
	cassava4.1_002980m.g	RNA.regulation of transcription.ARF, Auxin Response Factor family	auxin response factor 16	-1.15	-2.31	-0.70	-1.62	0.20	-2.20	-0.46	-3.21	-0.74	-3.09
	cassava4.1_034399m.g	RNA.regulation of transcription.bHLH,Basic Helix-Loop-Helix family	basic helix-loop-helix (bHLH) DNA-binding superfamily protein	-0.24	-1.87	-0.12	-1.67	0.03	-2.02	-0.20	-2.56	-0.57	-2.72
	cassava4.1_009094m.g	RNA.regulation of transcription.TCP transcription factor family	TEOSINTE BRANCHED, cycloidea and PCF (TCP) 14	-0.65	-2.69	-0.77	-2.23	0.03	-2.21	-0.77	-3.23	-0.58	-3.32
	cassava4.1_014849m.g	RNA.regulation of transcription.Aux/IAA family	indoleacetic acid-induced protein 16	-0.66	-1.99	-0.46	-1.76	-0.13	-1.65	-0.79	-2.01	-0.72	-3.23
	cassava4.1_007172m.g	RNA.regulation of transcription.C2C2(Zn) DOF zinc finger family	cycling DOF factor 3	-0.46	-3.27	-1.12	-3.09	-0.11	-2.50	-0.66	-3.22	-0.54	-4.11
	cassava4.1_031883m.g	RNA.regulation of transcription.Nucleosome/chromatin assembly factor group	HMG (high mobility group) box protein with ARID/BRIGHT DNA-binding domain	-1.58	-5.82	-2.26	-6.28	-0.86	-4.21	-0.87	-5.83	-0.98	-8.71
	cassava4.1_034343m.g	RNA.regulation of transcription.CCAAT box binding factor family, HAP5	nuclear factor Y, subunit C4	-0.26	-1.94	-0.12	-2.18	-0.25	-1.36	-0.19	-3.18	-0.68	-3.80
	cassava4.1_033968m.g	RNA.regulation of transcription.GRAS transcription factor family	GRAS family transcription factor family protein	-0.28	-1.23	-0.29	-1.09	-0.13	-0.86	-0.25	-1.57	-0.56	-1.68
	cassava4.1_024318m.g	RNA.regulation of transcription.TCP transcription factor family	TEOSINTE BRANCHED, cycloidea and PCF (TCP) 14	-0.38	-1.41	-0.52	-1.95	-0.02	-1.40	-0.77	-2.26	-0.86	-2.27

	cassava4.1_014022m.g	RNA.regulation of transcription.bZIP transcription factor family	Basic-leucine zipper (bZIP) transcription factor family protein	-0.43	-1.36	0.05	-0.54	-0.08	-0.15	-0.36	-1.20	-0.54	-1.14
	cassava4.1_012706m.g	RNA.regulation of transcription.Aux/IAA family	indole-3-acetic acid inducible 11	-0.38	-1.58	-0.23	-0.83	-0.27	-0.57	-0.12	-1.23	-0.75	-1.39
	cassava4.1_006027m.g	RNA.regulation of transcription.bHLH,Basic Helix-Loop-Helix family	basic helix-loop-helix (bHLH) DNA-binding superfamily protein	-0.39	-1.15	-0.22	-0.85	-0.11	-0.30	-0.13	-1.01	-0.45	-1.06
	cassava4.1_012686m.g	RNA.regulation of transcription.C2C2(Zn) YABBY family	Plant-specific transcription factor YABBY family protein	-1.23	-2.60	-0.65	-1.82	-0.31	-1.37	-0.51	-2.54	-0.99	-2.46
	cassava4.1_013899m.g	RNA.regulation of transcription.bHLH,Basic Helix-Loop-Helix family	basic helix-loop-helix (bHLH) DNA-binding superfamily protein	-0.98	-1.66	-0.34	-0.61	-0.21	-0.39	-0.01	-1.80	-0.26	-1.12
	cassava4.1_014204m.g	RNA.regulation of transcription.C2C2(Zn) GATA transcription factor family	GATA transcription factor 1	-0.60	-1.19	-0.27	-0.58	0.08	-0.34	-0.24	-1.15	-0.37	-0.86
	cassava4.1_007232m.g	RNA.regulation of transcription.Chromatin Remodeling Factors	switch subunit 3	-0.86	-1.43	-0.02	-0.70	0.06	-0.08	-0.30	-1.28	-0.21	-1.02
	cassava4.1_008380m.g	RNA.regulation of transcription.bHLH,Basic Helix-Loop-Helix family	basic helix-loop-helix (bHLH) DNA-binding superfamily protein	-0.69	-2.29	-0.13	-1.41	-0.31	-0.87	-0.01	-2.53	-0.09	-1.45
	cassava4.1_015296m.g	RNA.regulation of transcription.AP2/EREBP, APETALA2/Ethylene-responsive element binding protein family	Integrase-type DNA-binding superfamily protein	-0.31	-1.26	0.24	-0.64	0.03	-0.26	0.06	-1.63	-0.16	-1.16
	cassava4.1_012965m.g	RNA.regulation of transcription.C2C2(Zn) CO-like, Constans-like zinc finger family	B-box type zinc finger family protein	-0.50	-2.76	-0.30	-1.82	-0.51	-0.83	-0.31	-2.85	-0.51	-2.77
	cassava4.1_009861m.g	RNA.regulation of transcription.bHLH,Basic Helix-Loop-Helix family	basic helix-loop-helix (bHLH) DNA-binding superfamily protein	-0.89	-2.88	-0.79	-1.80	0.12	-0.28	-0.36	-3.19	-0.63	-2.03
	cassava4.1_009139m.g	RNA.regulation of transcription.E2F/DP transcription factor family	DP-E2F-like 1	-0.63	-1.37	-0.35	-1.03	-0.01	-0.54	-0.40	-1.66	-0.35	-1.06
	cassava4.1_026662m.g	RNA.regulation of transcription.G2-like transcription factor family, GARP	GBF's pro-rich region-interacting factor 1	-0.71	-2.74	-0.71	-1.71	-0.16	-0.85	-0.60	-2.44	-0.40	-1.44
	cassava4.1_015138m.g	RNA.regulation of transcription.bHLH,Basic Helix-Loop-Helix family	basic helix-loop-helix (bHLH) DNA-binding superfamily protein	-0.76	-2.37	-0.34	-0.82	-0.11	-0.81	-0.83	-1.73	-0.39	-1.14
	cassava4.1_007940m.g	RNA.regulation of transcription.C2C2(Zn) CO-like, Constans-like zinc finger family	B-box type zinc finger protein with CCT domain	-1.63	-5.53	-0.87	-2.94	0.09	-2.17	-0.86	-3.55	-0.57	-2.89
	cassava4.1_010137m.g	RNA.regulation of transcription.C2C2(Zn) CO-like, Constans-like zinc	CONSTANS-like 5	-1.03	-2.99	-0.76	-1.75	-0.13	-1.49	-0.68	-1.98	-0.48	-1.75

		finger family											
	cassava4.1_016105m.g	RNA.regulation of transcription.C2C2(Zn) YABBY family	Plant-specific transcription factor YABBY family protein	-1.73	-4.33	-0.86	-2.72	-0.31	-1.84	-0.71	-2.88	-0.77	-2.61
	cassava4.1_016118m.g	RNA.regulation of transcription.C2C2(Zn) YABBY family	Plant-specific transcription factor YABBY family protein	-1.43	-4.13	-0.54	-2.02	-0.04	-1.52	-0.93	-2.58	-0.78	-2.06
	cassava4.1_007858m.g	RNA.regulation of transcription.bHLH,Basic Helix-Loop-Helix family	cryptochrome-interacting basic-helix-loop-helix 1	-1.35	-3.98	-0.19	-1.80	-0.63	-1.15	-0.84	-2.62	-0.64	-2.58
	cassava4.1_007389m.g	RNA.regulation of transcription.HB,Homeobox transcription factor family	homeobox from Arabidopsis thaliana	-0.83	-3.35	0.36	-0.35	0.29	-0.57	0.12	-2.08	-0.16	-1.88
	cassava4.1_005961m.g	RNA.regulation of transcription.AP2/EREBP, APETALA2/Ethylene-responsive element binding protein family	Integrase-type DNA-binding superfamily protein	-1.00	-2.11	-0.45	-1.64	-0.04	-1.18	-1.12	-2.57	-1.07	-1.85
	cassava4.1_016924m.g	RNA.regulation of transcription.AP2/EREBP, APETALA2/Ethylene-responsive element binding protein family	DREB and EAR motif protein 2	-0.63	-1.15	-0.26	-0.80	0.37	-0.38	-0.48	-1.32	-0.58	-1.02
	cassava4.1_006460m.g	RNA.regulation of transcription.SET-domain transcriptional regulator family	Arabinanase/levansucrase/invertase	-1.19	-2.08	-0.30	-0.95	0.04	-1.20	-1.04	-2.27	-0.57	-1.66
	cassava4.1_016111m.g	RNA.regulation of transcription.C2C2(Zn) CO-like, Constans-like zinc finger family	B-box type zinc finger family protein	-0.47	-1.95	-0.58	-0.82	-0.08	-1.43	-0.65	-2.04	-0.32	-1.25
	cassava4.1_006618m.g	RNA.regulation of transcription.TCP transcription factor family	TEOSINTE BRANCHED 1, cycloidea and PCF transcription factor 2	-0.29	-0.85	-0.34	-0.36	0.10	-0.45	-0.37	-1.25	-0.08	-0.51
	cassava4.1_034131m.g	RNA.regulation of transcription.GRAS transcription factor family	GRAS family transcription factor	-1.37	-2.71	-1.33	-2.11	0.16	-0.14	-0.72	-2.30	-0.33	-1.66
	cassava4.1_001764m.g	RNA.regulation of transcription.HB,Homeobox transcription factor family	Homeobox-leucine zipper family protein / lipid-binding START domain-containing protein	-0.34	-1.01	-0.52	-1.12	0.03	-0.10	-0.05	-1.12	0.11	-0.52
	cassava4.1_004978m.g	RNA.regulation of transcription.CPP(Zn),CPP1-related transcription factor family	Tesmin/TSO1-like CXC domain-containing protein	-0.52	-1.16	-0.25	-0.53	0.08	-0.37	0.48	-0.99	-0.05	-0.53
	cassava4.1_015543m.g	RNA.regulation of transcription.MADS box transcription factor family	K-box region and MADS-box transcription factor family protein	-0.55	-0.87	-0.48	-0.59	-0.23	-0.35	-0.03	-1.05	-0.14	-0.52
	cassava4.1_032318m.g	RNA.regulation of transcription.MYB-related transcription factor family	Homeodomain-like superfamily protein	-0.44	-0.96	-0.85	-0.74	-0.22	0.21	-0.16	-1.12	0.01	-1.16

	cassava4.1_011576m.g	RNA.regulation of transcription.CCAAT box binding factor family, HAP2	nuclear factor Y, subunit A3	-0.69	-0.87	-1.14	-1.94	-0.05	-0.48	0.11	-1.40	-0.58	-1.51
	cassava4.1_011567m.g	RNA.regulation of transcription.HB,Homeobox transcription factor family	homeobox 1	-0.42	-1.25	-0.96	-2.26	0.19	-0.39	0.26	-1.95	-0.81	-2.88
	cassava4.1_032429m.g	RNA.regulation of transcription.bHLH,Basic Helix-Loop-Helix family	basic helix-loop-helix (bHLH) DNA-binding superfamily protein	-0.43	-0.63	-0.20	-1.12	-0.06	-0.21	0.27	-1.11	-0.15	-1.09
	cassava4.1_024743m.g	RNA.regulation of transcription.TCP transcription factor family	TCP family transcription factor	-0.89	-3.11	-1.14	-3.48	-0.05	-0.28	-0.18	-3.83	-0.85	-3.63
	cassava4.1_006715m.g	RNA.regulation of transcription.GRAS transcription factor family	scarecrow-like 3	-0.20	-0.74	-0.41	-1.16	0.02	-0.01	0.47	-0.71	0.19	-0.61
	cassava4.1_013408m.g	RNA.regulation of transcription.Trihelix, Triple-Helix transcription factor family	sequence-specific DNA binding transcription factors	-0.03	-0.69	-0.47	-0.94	0.11	-0.21	0.22	-0.43	-0.16	-0.64
C4	cassava4.1_007151m.g	RNA.regulation of transcription.AP2/EREBP, APETALA2/Ethylene-responsive element binding protein family	Integrase-type DNA-binding superfamily protein	1.10	0.22	0.63	1.01	-0.13	0.49	-0.05	-0.45	0.16	0.09
	cassava4.1_004047m.g	RNA.regulation of transcription.bZIP transcription factor family	Basic-leucine zipper (bZIP) transcription factor family protein	0.10	0.15	1.01	1.32	0.23	0.37	-0.47	-0.25	0.41	0.27
	cassava4.1_000920m.g	RNA.regulation of transcription.Argonaute	Argonaute family protein	0.43	0.67	0.68	1.48	0.29	0.71	0.19	0.46	0.32	0.50
	cassava4.1_000998m.g	RNA.regulation of transcription.JUMONJI family	transferases, transferring glycosyl groups	0.19	-0.11	0.59	1.73	0.01	0.38	0.23	-0.41	0.10	0.12
	cassava4.1_001443m.g	RNA.regulation of transcription.JUMONJI family	Transcription factor jumonji (jmj) family protein / zinc finger (C5HC2 type) family protein	-0.50	-0.55	0.08	0.94	-0.05	-0.14	-0.83	-2.01	-0.21	-1.18
	cassava4.1_015610m.g	RNA.regulation of transcription.MYB domain transcription factor family	myb domain protein 4	0.00	0.91	-0.08	1.48	0.23	1.17	-0.42	-0.64	0.32	-0.30
	cassava4.1_021785m.g	RNA.regulation of transcription.AP2/EREBP, APETALA2/Ethylene-responsive element binding protein family	ARIA-interacting double AP2 domain protein	0.26	1.35	0.00	0.78	-0.08	0.20	0.08	-0.47	0.23	-0.14
	cassava4.1_001357m.g	RNA.regulation of transcription.ARF, Auxin Response Factor family	auxin response factor 6	0.20	1.02	-0.09	-0.12	-0.19	-0.57	-0.42	-0.64	-0.13	-0.78
	cassava4.1_032607m.g	RNA.regulation of transcription.bZIP transcription factor family	bZIP transcription factor family protein	0.33	1.23	-0.16	-0.02	0.25	-0.78	-0.60	-1.45	-0.23	-0.88
	cassava4.1_012438m.g	RNA.regulation of transcription.AP2/EREBP, APETALA2/Ethylene-responsive element binding protein family	Integrase-type DNA-binding superfamily protein	0.87	1.83	0.12	0.06	0.08	0.36	-1.88	-1.92	-0.52	-1.42
	cassava4.1_009136m.g	RNA.regulation of transcription.C2C2(Zn) GATA transcription factor family	GATA transcription factor 26	0.27	0.49	0.10	0.11	-0.17	-0.18	-0.18	-0.95	-0.27	-0.53
	cassava4.1_012777m.g	RNA.regulation of transcription.HB,Homeobox transcription factor family	homeobox 1	0.71	0.85	-0.17	0.15	0.06	0.22	-0.11	-0.65	-0.13	-0.45

	cassava4.1_010485m.g	RNA.regulation of transcription.G2-like transcription factor family, GARP	Homeodomain-like superfamily protein	0.57	1.24	0.43	0.69	0.21	1.14	0.06	-0.42	-0.19	-0.65
	cassava4.1_015664m.g	RNA.regulation of transcription.SNF7	SNF7 family protein	0.74	1.04	0.43	0.97	0.52	0.65	0.13	0.46	-0.04	0.01
	cassava4.1_002672m.g	RNA.regulation of transcription.C3H zinc finger family	zinc finger (CCCH-type) family protein	1.27	0.89	0.58	0.65	0.27	0.81	0.44	1.07	-0.34	-0.46
	cassava4.1_002724m.g	RNA.regulation of transcription.C3H zinc finger family	zinc finger (CCCH-type) family protein	0.98	0.73	0.62	0.73	0.28	0.85	0.16	0.81	-0.19	-0.12
	cassava4.1_016728m.g	RNA.regulation of transcription.Aux/IAA family	indoleacetic acid-induced protein 16	0.70	0.33	0.73	1.00	0.43	1.41	0.27	0.44	-0.43	-0.37
	cassava4.1_012634m.g	RNA.regulation of transcription.HB,Homeobox transcription factor family	homeobox protein 6	1.26	1.10	0.64	1.20	0.25	2.19	0.78	1.53	-0.19	0.51
	cassava4.1_013436m.g	RNA.regulation of transcription.C2C2(Zn) GATA transcription factor family	GATA-type zinc finger protein with TIFY domain	-0.14	-0.60	-0.23	-0.26	0.06	0.39	-0.73	-0.79	-0.50	-1.51
	cassava4.1_032088m.g	RNA.regulation of transcription.TCP transcription factor family	TCP family transcription factor	0.06	-0.32	-0.45	-0.11	-0.04	1.66	0.12	-0.70	-0.58	-1.39
	cassava4.1_027174m.g	RNA.regulation of transcription.bHLH,Basic Helix-Loop-Helix family	basic helix-loop-helix (bHLH) DNA-binding superfamily protein	-1.54	-1.72	-0.27	-0.27	-1.59	-0.82	-1.38	-2.53	-0.13	-0.39
	cassava4.1_009849m.g	RNA.regulation of transcription.bHLH,Basic Helix-Loop-Helix family	basic helix-loop-helix (bHLH) DNA-binding superfamily protein	-0.55	-1.03	-0.34	-0.16	-0.09	-0.20	-0.10	-1.36	0.13	0.12
	cassava4.1_025309m.g	RNA.regulation of transcription.MADS box transcription factor family	AGAMOUS-like 30	-1.49	-1.66	-0.52	-0.30	-0.33	-1.25	-0.74	-2.71	-0.45	-0.90
	cassava4.1_009158m.g	RNA.regulation of transcription.bHLH,Basic Helix-Loop-Helix family	basic helix-loop-helix (bHLH) DNA-binding superfamily protein	-0.87	-0.74	-0.11	-0.16	-0.17	-0.14	-0.93	-2.68	0.33	-0.22
	cassava4.1_002081m.g	RNA.regulation of transcription.Trihelix, Triple-Helix transcription factor family	GT-2-like 1	-0.12	-0.10	-0.06	0.25	-0.03	-0.11	-0.11	-1.47	0.21	0.07
	cassava4.1_010157m.g	RNA.regulation of transcription.AP2/EREBP, APETALA2/Ethylene-responsive element binding protein family	AP2/B3 transcription factor family protein	0.79	-1.70	1.67	1.62	0.38	1.59	-0.26	0.59	1.53	1.00
	cassava4.1_004685m.g	RNA.regulation of transcription.bHLH,Basic Helix-Loop-Helix family	basic helix-loop-helix (bHLH) DNA-binding superfamily protein	-0.64	-1.64	1.11	1.41	0.26	0.59	-0.47	-0.65	0.39	0.47
	cassava4.1_002257m.g	RNA.regulation of transcription.CPP(Zn),CPP1-related transcription factor family	TESMIN/TSO1-like CXC 2	-2.50	-3.77	0.01	-0.72	-0.38	-1.39	-1.16	-2.58	-0.27	-0.56
	cassava4.1_015402m.g	RNA.regulation of transcription.AP2/EREBP,	Integrase-type DNA-binding	0.84	-2.58	2.35	2.59	0.38	0.89	-1.13	-0.25	2.08	-1.29

		APETALA2/Ethylene-responsive element binding protein family	superfamily protein										
	cassava4.1_003107m.g	RNA.regulation of transcription.C3H zinc finger family	zinc finger (CCCH-type) family protein	0.22	-1.28	1.84	0.87	0.69	-0.08	-0.21	0.11	0.84	-0.83
	cassava4.1_007562m.g	RNA.regulation of transcription.PHOR1	ARM repeat superfamily protein	-0.04	-1.34	2.03	1.52	0.87	0.27	0.28	-0.21	2.07	-1.14
	cassava4.1_002125m.g	RNA.regulation of transcription.GRAS transcription factor family	SCARECROW-like 14	0.06	-0.74	1.91	1.54	0.61	0.66	-0.42	-1.34	0.53	-2.13
	cassava4.1_034207m.g	RNA.regulation of transcription.PHOR1	CYS, MET, PRO, and GLY protein 2	-0.51	-1.40	0.79	0.45	0.21	0.01	-0.97	-1.06	-0.57	-1.56
	cassava4.1_005028m.g	RNA.regulation of transcription.GRAS transcription factor family	SCARECROW-like 13	-0.05	-0.99	0.49	0.02	0.26	-0.29	0.06	-0.56	-0.22	-0.63
	cassava4.1_011430m.g	RNA.regulation of transcription.C2C2(Zn) GATA transcription factor family	GATA transcription factor 5	-0.12	-1.46	1.43	0.30	0.38	-0.60	-0.33	-1.35	-0.18	-1.81
	cassava4.1_002197m.g	RNA.regulation of transcription.GRAS transcription factor family	SCARECROW-like 14	-0.09	-0.81	0.64	0.29	0.19	-0.19	-0.24	-0.59	-0.03	-1.19
	cassava4.1_003039m.g	RNA.regulation of transcription.C3H zinc finger family	zinc finger (CCCH-type) family protein	-0.33	-2.24	1.50	0.21	0.54	-0.30	-0.59	-1.83	0.27	-2.04
	cassava4.1_005031m.g	RNA.regulation of transcription.GRAS transcription factor family	SCARECROW-like 13	-0.21	-1.30	0.73	0.06	0.41	-0.19	-0.06	-1.12	-0.14	-1.22
	cassava4.1_023451m.g	RNA.regulation of transcription.Trihelix, Triple-Helix transcription factor family	Alcohol dehydrogenase transcription factor Myb/SANT-like family protein	-0.27	-1.17	0.58	-0.31	0.30	-0.04	-0.73	-0.90	-0.14	-0.70
	cassava4.1_028155m.g	RNA.regulation of transcription.GRAS transcription factor family	GRAS family transcription factor	-0.04	-1.03	-0.26	-0.69	-0.16	-0.43	-2.04	-1.32	-1.00	-0.82
	cassava4.1_022726m.g	RNA.regulation of transcription.AP2/EREBP, APETALA2/Ethylene-responsive element binding protein family	Integrase-type DNA-binding superfamily protein	-0.31	-0.50	-0.62	-0.66	0.58	-0.08	-1.30	-1.45	0.01	-0.42
	cassava4.1_015251m.g	RNA.regulation of transcription.C2H2 zinc finger family	C2H2 and C2HC zinc fingers superfamily protein	-0.73	-0.92	-0.62	-1.35	-0.09	-0.61	-1.16	-1.80	-0.42	-0.57
	cassava4.1_025356m.g	RNA.regulation of transcription.C2C2(Zn) GATA transcription factor family	Unknow	-0.72	-1.38	-0.62	-0.78	0.18	0.97	-1.88	-2.88	0.21	-0.65
	cassava4.1_013285m.g	RNA.regulation of transcription.HB,Homeobox transcription factor family	Homeobox-leucine zipper protein family	-0.59	-1.07	-0.61	-1.19	-0.28	-0.27	-1.37	-1.26	-0.39	-0.74
	cassava4.1_027347m.g	RNA.regulation of transcription.TCP transcription factor family	TEOSINTE BRANCHED, cycloidea and PCF (TCP) 14	-1.69	-1.82	-0.69	-1.10	0.12	-0.47	-1.71	-1.81	-1.13	-1.62
	cassava4.1_014770m.g	RNA.regulation of transcription.AS2,Lateral Organ Boundaries Gene Family	LOB domain-containing protein 37	-1.12	-1.94	-0.62	0.33	-0.19	1.33	-0.96	-2.03	-0.72	-0.60

	cassava4.1_011546m.g	RNA.regulation of transcription.Trihelix, Triple-Helix transcription factor family	sequence-specific DNA binding transcription factors	-0.12	-0.40	-0.12	0.36	0.18	0.71	-0.48	-1.06	-0.46	-0.05
Signalling.light	cassava4.1_000531m.g	signalling.light	phytochrome B	0.57	1.36	0.70	2.04	0.12	2.45	-0.42	0.57	0.03	1.47
	cassava4.1_000570m.g	signalling.light	phytochrome C	-0.16	-0.13	-0.25	-0.36	-0.03	-0.32	-0.01	-0.90	0.02	-0.34
	cassava4.1_007276m.g	signalling.light	photolyase/blue-light receptor 2	-0.02	0.66	0.91	2.56	0.06	3.38	-0.62	1.67	-0.02	2.32
	cassava4.1_007222m.g	signalling.light	photolyase/blue-light receptor 2	-0.58	-1.23	-0.37	-0.17	-0.22	0.62	-0.36	-0.48	-0.24	-0.15
	cassava4.1_030861m.g	signalling.light	phytochrome kinase substrate 1	-0.69	-1.03	-0.92	-1.87	0.10	-1.69	-0.68	-2.01	-0.69	-3.10
	cassava4.1_003609m.g	signalling.light	Phototropic-responsive NPH3 family protein	-1.20	-1.48	-0.56	-0.39	0.01	-0.49	-0.28	-2.47	0.20	-0.79
	cassava4.1_003684m.g	signalling.light	Phototropic-responsive NPH3 family protein	-0.75	-2.01	-0.68	-1.09	0.00	-0.24	-0.37	-1.34	-0.15	-1.00
	cassava4.1_032028m.g	signalling.light	Phototropic-responsive NPH3 family protein	-1.81	-4.05	-1.07	-3.01	-0.57	-3.08	-0.73	-5.29	-1.08	-4.15
	cassava4.1_004505m.g	signalling.light	Phototropic-responsive NPH3 family protein	-0.33	-0.46	-0.38	-0.23	-0.18	-0.87	0.01	-1.57	-0.22	-1.18
	cassava4.1_016715m.g	signalling.light	Chlorophyll A-B binding family protein	-0.66	-3.07	0.12	-1.35	-0.09	-0.59	1.96	2.01	0.37	0.85
	cassava4.1_003032m.g	signalling.light	cryptochrome 1	-0.47	-1.14	-0.44	-0.88	-0.09	-0.78	-0.07	-1.03	-0.42	-1.40
	cassava4.1_002935m.g	signalling.light	cryptochrome 1	-0.40	-1.10	-0.38	-1.07	-0.07	-0.81	-0.06	-1.45	-0.42	-1.49
	cassava4.1_023566m.g	signalling.light	PAS/LOV protein B	0.16	-0.26	0.25	0.01	-0.18	-0.47	-0.44	-1.18	-0.29	-0.93
	cassava4.1_002824m.g	signalling.light	FAR1-related sequence 10	-0.48	-0.60	-0.37	-0.36	0.02	-0.84	-0.23	-1.31	-0.07	-0.84
	cassava4.1_027812m.g	signalling.light	Phototropic-responsive NPH3 family protein	-1.75	-3.86	-1.69	-1.16	-0.06	-4.04	-1.29	-3.04	-0.85	-3.13
	cassava4.1_008267m.g	signalling.light	Phototropic-responsive NPH3 family protein	-0.83	-1.62	-0.01	-0.48	0.08	-1.36	-1.25	0.68	-0.24	0.93
	cassava4.1_004180m.g	signalling.light	Phototropic-responsive NPH3 family protein	-0.91	0.30	-0.91	-1.22	-0.10	-1.90	-1.08	-1.76	-0.10	-1.14
	cassava4.1_005641m.g	signalling.light	Phototropic-responsive NPH3 family	-0.91	-1.08	-0.35	-0.88	-0.12	-0.98	-0.92	-1.34	-0.53	-1.24

			protein										
	cassava4.1_003719m.g	signalling.light	Phototropic-responsive NPH3 family protein	0.17	-0.19	0.36	1.33	0.27	2.16	-0.61	0.68	0.59	2.44
	cassava4.1_025564m.g	signalling.light	Phototropic-responsive NPH3 family protein	-0.02	-1.07	0.42	-0.29	-0.10	-0.61	-1.04	-0.67	-0.38	-0.95
	cassava4.1_005067m.g	signalling.light	Phototropic-responsive NPH3 family protein	-0.22	-1.03	0.32	0.41	0.41	0.82	-0.38	0.84	0.08	1.19
	cassava4.1_004452m.g	signalling.light	Phototropic-responsive NPH3 family protein	-0.65	-1.83	-0.37	-1.08	-0.12	-1.19	-0.46	-1.78	-0.49	-1.74
	cassava4.1_000918m.g	signalling.light	SPA (suppressor of phyA-105) protein family	-1.29	-3.25	-0.44	-2.06	-0.11	-2.54	-0.43	-3.24	-0.30	-2.80
	cassava4.1_033918m.g	signalling.light	SPA1-related 3	-0.76	-2.26	-0.40	-1.87	-0.07	-2.19	0.14	-2.55	-0.16	-1.88
	cassava4.1_001319m.g	signalling.light	SPA1-related 3	-0.37	-0.99	-0.16	-0.28	-0.04	-0.97	0.03	-1.69	-0.24	-1.22
	cassava4.1_017713m.g	signalling.light	CCR-like	-0.22	-2.80	-0.02	-5.12	0.08	-5.10	-0.04	-2.66	-0.28	-3.40

Supplemental Table S8. Genes annotated to redox pathway. The extent of differential expression is measured in terms of log2 fold change of treatment vs. control.

Cassava ID	Mapman Category	Short Description	PG0	FG0	PG2	FG2	PG4	FG4	PG0	FG0	PG2	FG2
			120 day						45 day			
cassava4.1_024509m.g	redox.ascorbate and glutathione.ascorbate	ascorbate peroxidase 2	-5.35	-6.59	-4.49	-7.32	-0.90	-2.93	1.30	-0.30	-0.07	-1.95
cassava4.1_027646m.g	redox.ascorbate and glutathione.ascorbate	ascorbate peroxidase 4	-1.36	-2.69	-0.52	-3.09	-0.10	-4.20	-0.48	-2.85	-0.61	-2.03
cassava4.1_006367m.g	redox.ascorbate and glutathione.ascorbate	monodehydroascorbate reductase 6	-1.22	-1.63	-0.41	-0.87	-0.10	-0.83	-0.18	-1.95	-0.37	-1.44

cassava4.1_022665m.g	redox.ascorbate and glutathione.ascorbate	ascorbate peroxidase 4	-1.16	-2.91	-0.18	-2.38	-0.01	-3.35	-0.78	-3.22	-0.72	-2.18
cassava4.1_016900m.g	redox.ascorbate and glutathione.ascorbate	thylakoidal ascorbate peroxidase	-1.04	-2.89	-0.74	-2.35	0.03	-2.11	-0.46	-2.44	-0.73	-2.45
cassava4.1_015741m.g	redox.ascorbate and glutathione.ascorbate	stromal ascorbate peroxidase	-0.88	-2.68	-0.45	-2.35	-0.10	-2.17	-0.49	-2.77	-0.86	-2.66
cassava4.1_005802m.g	redox.ascorbate and glutathione.ascorbate	monodehydroascorbate reductase 4	-0.54	-1.66	-0.33	-1.38	0.07	-1.15	-0.15	-1.69	-0.41	-1.80
cassava4.1_013773m.g	redox.ascorbate and glutathione.ascorbate	dehydroascorbate reductase 1	-0.41	-1.13	-0.25	-1.02	-0.06	-0.84	-0.47	-1.07	-0.54	-1.34
cassava4.1_013184m.g	redox.ascorbate and glutathione.ascorbate	ascorbate peroxidase 3	-0.33	-0.68	-0.24	-0.52	0.01	-0.74	-0.21	-1.13	-0.47	-1.18
cassava4.1_016102m.g	redox.ascorbate and glutathione.ascorbate	dehydroascorbate reductase 2	-0.13	-0.29	-0.27	-0.96	0.05	-0.36	0.03	-0.15	-0.66	-1.04
cassava4.1_013461m.g	redox.ascorbate and glutathione.ascorbate	ascorbate peroxidase 1	0.95	0.72	0.59	0.96	0.18	1.28	-0.06	0.69	0.00	0.84
cassava4.1_014423m.g	redox.ascorbate and glutathione.ascorbate	ascorbate peroxidase 1	0.61	0.15	0.32	0.13	0.04	1.26	0.87	0.23	-0.14	-0.03
cassava4.1_009867m.g	redox.ascorbate and glutathione.ascorbate	stromal ascorbate peroxidase	1.59	1.68	0.86	1.55	0.10	1.59	-0.18	1.55	0.46	1.94
cassava4.1_006694m.g	redox.ascorbate and glutathione.ascorbate	monodehydroascorbate reductase 1	0.73	1.12	0.70	1.13	0.08	0.70	-0.14	1.53	0.11	0.97
cassava4.1_029735m.g	redox.ascorbate and glutathione.glutathione	glutamate-cysteine ligase	-0.93	-1.50	-0.67	-0.52	-0.67	-2.36	-0.58	-2.78	-0.08	-1.17
cassava4.1_006560m.g	redox.ascorbate and glutathione.glutathione	glutathione synthetase 2	-0.78	-0.59	-0.70	-0.69	0.12	-0.21	-0.45	-2.31	-0.39	-1.18
cassava4.1_015084m.g	redox.ascorbate and glutathione.glutathione	glutathione peroxidase 1	-0.10	-0.95	-0.25	-1.60	0.04	-1.86	0.58	-1.05	-0.17	-1.41

cassava4.1_012839m.g	redox.ascorbate and glutathione.glutathione	glutathione-disulfide reductase	0.08	0.54	-0.06	0.22	-0.08	0.59	-0.05	1.07	0.62	0.94
cassava4.1_017755m.g	redox.ascorbate and glutathione.glutathione	glutathione peroxidase 6	1.14	1.53	0.50	0.49	0.22	1.14	0.55	1.50	-0.12	0.43
cassava4.1_030777m.g	redox.dismutases and catalases	catalase 2	-0.56	-2.94	-0.09	-1.99	-0.15	-2.45	0.02	-1.67	-0.74	-3.05
cassava4.1_018294m.g	redox.dismutases and catalases	copper/zinc superoxide dismutase 1	0.61	0.50	0.04	0.00	0.02	-0.20	0.96	0.36	0.34	0.19
cassava4.1_018049m.g	redox.dismutases and catalases	copper/zinc superoxide dismutase 3	-0.68	-1.32	-0.55	-0.96	0.14	-0.52	-0.11	-0.52	-0.43	-0.67
cassava4.1_006297m.g	redox.dismutases and catalases	catalase 2	-0.39	-2.44	-0.40	-2.08	-0.04	-1.87	-0.16	-1.66	-0.82	-2.61
cassava4.1_024232m.g	redox.glutaredoxins	Thioredoxin superfamily protein	-1.19	-2.99	0.03	-3.20	-0.97	-2.34	-0.15	-5.26	-1.16	-2.78
cassava4.1_008955m.g	redox.glutaredoxins	Glutaredoxin family protein	-0.82	-1.35	-0.13	-0.70	-0.33	-0.62	-0.43	-1.35	-0.43	-0.70
cassava4.1_033785m.g	redox.glutaredoxins	Thioredoxin superfamily protein	-0.69	-0.30	-0.37	0.27	-0.54	-0.59	1.40	-0.86	-0.59	0.06
cassava4.1_014034m.g	redox.glutaredoxins	Glutaredoxin family protein	-0.62	-0.85	-0.55	-0.88	-0.07	-0.16	0.03	-0.66	-0.16	-0.57
cassava4.1_034311m.g	redox.glutaredoxins	Glutaredoxin family protein	-0.62	-2.36	-0.29	-1.46	0.02	-0.67	-0.07	-1.32	-0.49	-1.80
cassava4.1_024311m.g	redox.glutaredoxins	Glutaredoxin family protein	-0.51	-1.64	-0.51	-0.74	0.07	-0.44	-0.48	-1.33	0.01	0.00
cassava4.1_018918m.g	redox.glutaredoxins	Thioredoxin superfamily protein	-0.43	-0.96	-1.38	-1.65	-0.02	-1.11	-1.14	-2.36	-0.61	-2.08
cassava4.1_019956m.g	redox.glutaredoxins	Thioredoxin superfamily protein	-0.11	-1.65	-0.50	-1.87	0.04	-1.84	-0.33	-1.08	-0.46	-1.58
cassava4.1_019954m.g	redox.glutaredoxins	Thioredoxin superfamily protein	0.59	0.35	-0.26	-1.05	0.00	-1.60	-0.38	-0.84	-1.02	-2.56
cassava4.1_002749m.g	redox.glutaredoxins	glutaredoxin-related	0.01	1.09	0.23	0.99	0.13	0.58	-0.23	-0.01	-0.53	-0.64
cassava4.1_018797m.g	redox.glutaredoxins	Glutaredoxin family protein	0.03	-0.06	0.39	0.77	-0.06	1.04	-0.26	-0.02	-0.25	0.44
cassava4.1_019777m.g	redox.glutaredoxins	Glutaredoxin family protein	0.58	1.35	0.58	0.68	0.18	0.19	0.18	1.36	-0.01	0.84
cassava4.1_018177m.g	redox.glutaredoxins	Thioredoxin superfamily protein	1.07	-0.59	1.52	1.73	0.36	0.47	-0.85	0.17	0.79	-0.26
cassava4.1_016021m.g	redox.peroxiredoxin	Thioredoxin superfamily protein	-1.44	-2.73	-0.72	-2.58	-0.23	-2.89	-1.03	-2.49	-1.05	-2.52
cassava4.1_016588m.g	redox.peroxiredoxin	peroxiredoxin IIF	-0.08	-0.12	-0.14	0.04	0.07	1.03	0.11	1.12	-0.18	0.91
cassava4.1_015438m.g	redox.peroxiredoxin	Thioredoxin superfamily protein	-0.98	-2.91	-0.38	-1.40	-0.04	-0.69	-0.74	-1.94	-0.98	-1.98
cassava4.1_013279m.g	redox.thioredoxin	atypical CYS HIS rich thioredoxin 4	-1.27	-2.10	-0.69	-2.64	-0.30	-3.70	-0.03	-4.18	-0.67	-3.58
cassava4.1_005499m.g	redox.thioredoxin	NADPH-dependent thioredoxin reductase C	-0.98	-1.69	-0.24	-1.13	-0.10	-1.05	-0.61	-2.13	-0.45	-1.51
cassava4.1_027763m.g	redox.thioredoxin	ferredoxin/thioredoxin reductase subunit A (variable subunit) 2	-0.93	-2.14	-1.24	-3.01	0.22	-1.31	-0.04	-2.04	-0.80	-2.16

cassava4.1_017096m.g	redox.thioredoxin	Thioredoxin superfamily protein	-0.92	-3.73	-0.64	-4.13	-0.01	-4.36	-0.11	-2.53	-0.98	-3.80
cassava4.1_015744m.g	redox.thioredoxin	ferredoxin/thioredoxin reductase subunit A (variable subunit) 2	-0.69	-1.82	-0.60	-1.49	-0.10	-1.03	-0.17	-1.53	-0.53	-1.19
cassava4.1_021142m.g	redox.thioredoxin	ferredoxin thioredoxin reductase catalytic beta chain family protein	-0.67	-1.89	-0.18	-1.98	0.10	-1.70	-0.36	-1.49	-0.93	-2.37
cassava4.1_018403m.g	redox.thioredoxin	ferredoxin thioredoxin reductase catalytic beta chain family protein	-0.63	-1.37	-0.41	-1.25	-0.06	-0.69	-0.43	-1.10	-0.51	-0.95
cassava4.1_015817m.g	redox.thioredoxin	Thioredoxin superfamily protein	-0.62	-2.30	-0.56	-1.61	-0.17	-1.09	-0.28	-1.79	-0.60	-1.49
cassava4.1_012905m.g	redox.thioredoxin	atypical CYS HIS rich thioredoxin 4	-0.56	-1.55	-0.37	-2.49	-0.14	-2.88	0.38	-2.78	-0.73	-3.14
cassava4.1_017711m.g	redox.thioredoxin	Rubredoxin-like superfamily protein	-0.49	-1.69	-0.31	-1.04	-0.03	-0.80	-0.22	-0.71	-0.42	-0.95
cassava4.1_011786m.g	redox.thioredoxin	APR-like 4	-0.48	-1.04	0.18	0.11	0.15	0.07	-0.29	-0.57	-0.53	-0.78
cassava4.1_016798m.g	redox.thioredoxin	WCRKC thioredoxin 1	-0.46	-1.05	-0.30	-2.42	0.06	-2.89	0.01	-1.13	-0.43	-1.90
cassava4.1_012801m.g	redox.thioredoxin	chloroplastic drought-induced stress protein of 32 kD	-0.42	-2.36	-0.27	-2.49	0.13	-1.85	-0.57	-1.02	-0.76	-1.95
cassava4.1_017734m.g	redox.thioredoxin	thioredoxin Y2	-0.33	-0.32	-0.31	-0.74	0.08	-0.88	-0.43	-0.84	-0.77	-1.44
cassava4.1_015481m.g	redox.thioredoxin	atypical CYS HIS rich thioredoxin 2	-0.32	-0.86	-0.32	-0.60	-0.10	-0.42	-0.21	-0.06	-0.32	-0.30
cassava4.1_016825m.g	redox.thioredoxin	thioredoxin F2	-0.23	-1.12	-0.13	-1.26	0.02	-1.36	-0.31	-1.31	-0.61	-1.82
cassava4.1_008355m.g	redox.thioredoxin	PDI-like 1-1	0.07	0.32	0.80	1.01	0.01	0.85	-0.25	0.58	0.13	1.33
cassava4.1_006109m.g	redox.thioredoxin	PDI-like 1-1	0.18	0.49	0.64	1.24	0.40	1.27	0.65	1.76	0.85	2.54
cassava4.1_019244m.g	redox.thioredoxin	C-terminal cysteine residue is changed to a serine 1	0.23	-0.34	-0.69	-1.66	-0.19	-1.74	0.15	0.21	-0.56	-0.85
cassava4.1_007986m.g	redox.thioredoxin	PDI-like 2-2	0.30	0.54	0.62	1.01	-0.10	1.06	-0.51	0.39	0.31	1.58
cassava4.1_016073m.g	redox.thioredoxin	thioredoxin domain-containing protein 9 homolog	0.39	0.97	0.45	0.83	-0.07	-0.09	-0.60	1.29	0.06	0.83
cassava4.1_015299m.g	redox.thioredoxin	atypical CYS HIS rich thioredoxin 2	0.46	-0.66	0.63	0.51	-0.01	0.48	0.24	1.15	-0.20	0.82
cassava4.1_010463m.g	redox.thioredoxin	thioredoxin family protein	0.51	1.22	1.43	1.78	-0.11	1.57	-0.30	1.09	0.75	2.78
cassava4.1_018507m.g	redox.thioredoxin	thioredoxin H-type 1	0.97	1.28	0.35	0.77	0.11	0.95	0.74	1.30	-0.85	-0.09
cassava4.1_017131m.g	redox.thioredoxin	thioredoxin M-type 4	1.05	1.27	0.23	0.14	0.11	0.30	0.20	0.55	-0.11	0.29
cassava4.1_018594m.g	redox.thioredoxin	thioredoxin H-type 7	1.05	1.83	2.31	3.90	-0.03	3.12	-2.60	2.04	0.43	3.38
cassava4.1_016044m.g	misc.glutathione S transferases	glutathione S-transferase F11	-6.30	-2.95	-1.90	-5.44	-0.70	-3.41	-3.37	-4.08	-1.25	-4.67

cassava4.1_015784m.g	misc.glutathione S transferases	glutathione S-transferase TAU 25	-1.37	-4.34	-0.92	-5.09	0.01	-4.70	-0.37	-2.78	-0.82	-3.50
cassava4.1_014463m.g	misc.glutathione S transferases	glutathione S-transferase F11	-1.09	-2.73	-1.11	-2.87	-0.10	-1.62	-1.16	-1.59	-0.60	-1.72
cassava4.1_012409m.g	misc.glutathione S transferases	Glutathione S-transferase family protein	-0.69	-1.22	-0.09	-0.88	-0.16	-1.09	-0.72	-1.60	-0.86	-1.42
cassava4.1_015854m.g	misc.glutathione S transferases	glutathione S-transferase TAU 19	-0.66	-1.50	-1.58	-2.29	-0.49	0.04	0.37	1.00	0.11	0.84
cassava4.1_015612m.g	misc.glutathione S transferases	glutathione S-transferase tau 7	-0.46	-2.78	-0.04	-2.85	0.17	-2.70	-0.86	-2.27	-0.60	-2.63
cassava4.1_014626m.g	misc.glutathione S transferases	glutathione S-transferase THETA 2	-0.43	-0.49	-0.23	-0.49	0.00	-0.93	0.08	-0.04	0.00	-0.33
cassava4.1_015872m.g	misc.glutathione S transferases	glutathione S-transferase TAU 19	-0.23	-1.57	-0.14	-0.95	-0.07	-0.40	-0.29	-0.66	-0.72	-1.37
cassava4.1_015406m.g	misc.glutathione S transferases	Glutathione S-transferase family protein	-0.11	0.68	-0.12	0.38	-0.21	2.00	-0.06	1.38	0.64	2.35
cassava4.1_018515m.g	misc.glutathione S transferases	microsomal glutathione s-transferase, putative	-0.08	0.19	-0.59	-0.62	0.16	0.39	0.05	1.35	0.09	0.74
cassava4.1_015709m.g	misc.glutathione S transferases	glutathione S-transferase tau 7	0.13	-0.60	0.17	-0.62	-0.18	-0.97	0.29	-1.07	-0.44	-1.61
cassava4.1_008745m.g	misc.glutathione S transferases	Glutathione S-transferase family protein	0.81	1.09	0.07	0.20	-0.05	0.14	-0.17	1.21	0.40	0.79
cassava4.1_015861m.g	misc.glutathione S transferases	glutathione S-transferase TAU 8	1.00	1.69	1.13	1.53	-0.04	0.50	1.29	1.23	-2.08	-0.47
cassava4.1_015761m.g	misc.glutathione S transferases	glutathione S-transferase TAU 25	1.85	0.12	0.73	0.16	0.24	-0.54	0.90	0.03	-1.26	-2.41
cassava4.1_016175m.g	misc.glutathione S transferases	glutathione S-transferase PHI 9	0.83	1.16	0.70	1.10	0.16	1.81	0.84	2.28	0.49	1.97
cassava4.1_007092m.g	misc.glutathione S transferases	glutathione S-transferase tau 7	1.32	2.15	0.30	0.09	-0.68	1.25	0.02	1.89	0.96	2.51
